# Cancers and COVID-19 Risk: A Mendelian Randomization Study

**DOI:** 10.3390/cancers14092086

**Published:** 2022-04-22

**Authors:** Zengbin Li, Yudong Wei, Guixian Zhu, Mengjie Wang, Lei Zhang

**Affiliations:** 1 China-Australia Joint Research Centre for Infectious Diseases, School of Public Health, Xi’an Jiaotong University Health Science Centre, Xi’an 710061, China; zengbinli@stu.xjtu.edu.cn (Z.L.); weiyudong@stu.xjtu.edu.cn (Y.W.); xianxianshell@stu.xjtu.edu.cn (G.Z.); mjwang0211@stu.xjtu.edu.cn (M.W.); 2 Melbourne Sexual Health Centre, Alfred Health, Melbourne, VIC 3053, Australia; 3 Central Clinical School, Faculty of Medicine, Nursing and Health Sciences, Monash University, Melbourne, VIC 3800, Australia; 4 Department of Epidemiology and Biostatistics, College of Public Health, Zhengzhou University, Zhengzhou 450001, China

**Keywords:** cancer, COVID-19, SARS-CoV-2, causal association, Mendelian randomization

## Abstract

**Simple Summary:**

During the COVID-19 pandemic, cancer patients are regarded as a highly vulnerable population. Given the unavoidable bias and unmeasured confounders in observational studies, the causal effects of cancers on COVID-19 outcomes are largely unknown. In the study, we tried to evaluate the causal effects of cancers on COVID-19 outcomes using the Mendelian randomization (MR) approach. No strong evidence was observed to support a causal role of cancer in COVID-19 development. Previous observational correlations between cancers and COVID-19 outcomes were likely confounded. Large and well-conducted epidemiological studies are required to determine whether cancers causally contribute to increased risk of COVID-19.

**Abstract:**

Observational studies have shown increased COVID-19 risk among cancer patients, but the causality has not been proven yet. Mendelian randomization analysis can use the genetic variants, independently of confounders, to obtain causal estimates which are considerably less confounded. We aimed to investigate the causal associations of cancers with COVID-19 outcomes using the MR analysis. The inverse-variance weighted (IVW) method was employed as the primary analysis. Sensitivity analyses and multivariable MR analyses were conducted. Notably, IVW analysis of univariable MR revealed that overall cancer and twelve site-specific cancers had no causal association with COVID-19 severity, hospitalization or susceptibility. The corresponding *p*-values for the casual associations were all statistically insignificant: overall cancer (*p* = 0.34; *p* = 0.42; *p* = 0.69), lung cancer (*p* = 0.60; *p* = 0.37; *p* = 0.96), breast cancer (*p* = 0.43; *p* = 0.74; *p* = 0.43), endometrial cancer (*p* = 0.79; *p* = 0.24; *p* = 0.83), prostate cancer (*p* = 0.54; *p* = 0.17; *p* = 0.58), thyroid cancer (*p* = 0.70; *p* = 0.80; *p* = 0.28), ovarian cancer (*p* = 0.62; *p* = 0.96; *p* = 0.93), melanoma (*p* = 0.79; *p* = 0.45; *p* = 0.82), small bowel cancer (*p* = 0.09; *p* = 0.08; *p* = 0.19), colorectal cancer (*p* = 0.85; *p* = 0.79; *p* = 0.30), oropharyngeal cancer (*p* = 0.31; not applicable, NA; *p* = 0.80), lymphoma (*p* = 0.51; NA; *p* = 0.37) and cervical cancer (*p* = 0.25; *p* = 0.32; *p* = 0.68). Sensitivity analyses and multivariable MR analyses yielded similar results. In conclusion, cancers might have no causal effect on increasing COVID-19 risk. Further large-scale population studies are needed to validate our findings.

## 1. Introduction

Coronavirus disease 2019 (COVID-19) is a global pandemic caused by the severe acute respiratory syndrome coronavirus 2 (SARS-CoV-2) [1]. As of April 2022, the cumulative cases and deaths of COVID-19 have reached over 500 million and 6 million, respectively [2]. Notably, COVID-19 individuals are mostly presented with mild and moderate infection, but can progress rapidly from asymptomatic to acute respiratory distress syndrome, multiple organ dysfunction syndrome and even death [3,4]. Therefore, identifying the potential risk factors for COVID-19 will be of significant value for public health and health policy.

Cancer patients are a vulnerable population during the COVID-19 pandemic [5,6]. Cancer represents a severe public health problem and is the second leading cause of death worldwide [7]. The Global Cancer Observatory estimated 19.3 million new cancer diagnoses and roughly 10.0 million cancer-associated deaths globally in 2020 [8]. Previous studies suggested that cancer patients showed higher prevalence, severe illness incidence, and mortality rate of COVID-19 compared with the non-cancer population [9,10,11]. However, a prospective cohort of 0.5 million people indicated that confounders—including socioeconomic status, age, and ethnicity—might interfere with the associations between COVID-19 and risk factors [12]. It was unclear whether the positive correlations between cancers and COVID-19 outcomes resulted from confounders or biases [13]. Furthermore, associations are correlative only; they do not imply causality.

Mendelian randomization, an epidemiological method, has been widely applied to assess the potential causal association between exposure and outcome [14,15]. According to Mendel’s law, genetic variants are randomly allocated at meiosis [16]. MR analysis, using genetic variants as instrumental variables (IVs), can minimize the influence of confounders or reverse causations [14]. Given the limitations of current research, we tried to evaluate the potential impact and the causal associations of cancers with COVID-19 outcomes using the MR method.

## 2. Materials and Methods

### 2.1. Study Design

**Figure 1:**
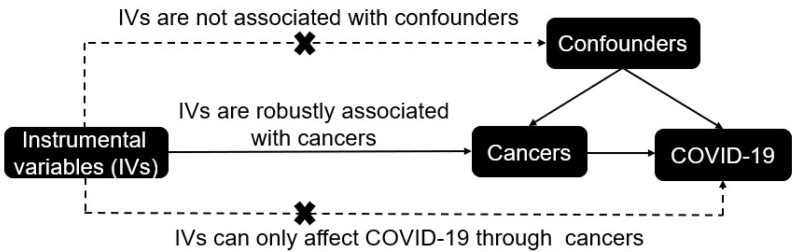
The overall design of the Mendelian randomization study.

Figure 1 outlines the overall design of investigating the causal associations between cancers and COVID-19 outcomes through MR study. Briefly, the MR method comprises two main steps: first, randomizing participants on the basis of IVs; then, assessing the causal associations between cancers and COVID-19 outcomes [14,17]. IVs should meet three key assumptions: (1) the IVs are robustly associated with cancers; (2) the IVs are not associated with confounders; and (3) the IVs should affect the outcomes of COVID-19 only through cancers, not via alternative pathways [17]. Previous MR studies have shown that some single nucleotide polymorphisms (SNPs) for cancers might be associated with confounders between cancers and COVID-19, such as educational attainment [18,19], body mass index (BMI) [20], income [18], alcohol consumption [21] and smoking [22,23]. Thus, we performed multivariable MR analyses to limit the effects of potential confounders.

### 2.2. Data Sources

The summary statistics in the genome-wide association studies (GWASs) for COVID-19 were sourced from the COVID-19 Host Genetics Initiative V5 [24], which excluded “23andMe” data. The COVID-19 GWAS data has been adjusted for age, gender, age^2^, age × gender, principal components and study-specific covariates by the original GWAS researchers. The COVID-19 outcomes included 1,683,768 participants (38,984 infection cases and 1,644,784 controls) for susceptibility, 1,887,658 participants (9986 hospitalized patients and 1,877,672 controls) for hospitalization, and 1,388,342 participants (5101 very serious respiratory confirmed patients and 1,383,241 controls) for severity, respectively. The uninfected individuals served as the controls. All cases were confirmed by laboratory, self-reported, or physician diagnosis. The severe cases were defined as patients who died or required respiratory support with COVID-19 infection [24].

The summary statistics of the GWASs for cancers were obtained from the UK biobank [25], International Lung Cancer Consortium (ILCCO) [26], Breast Cancer Association Consortium (BCAC) [27], Ovarian Cancer Association Consortium (OCAC) [28], Endometrial Cancer Association Consortium (ECAC) [29], Prostate Cancer Association Group to Investigate Cancer Associated Alterations in the Genome Consortium (PRACTICALC) [30] and the thyroid cancer study of Kohler et al. [31]. Overall cancer and 12 site-specific cancers were included: 336,272 participants for overall cancer, 27,209 participants for lung cancer, 18,313 participants for squamous cell lung cancer, 228,951 participants for breast cancer, 175,475 participants for estrogen receptor-positive (ER+) breast cancer, 127,442 participants for ER- breast cancer, 66,450 participants for ovarian cancer, 121,885 participants for endometrial cancer, 140,254 participants for prostate cancer, 1080 participants for thyroid cancer, 375,767 participants for melanoma, 337,159 participants for small bowel cancer, 377,673 participants for colorectal cancer, 372,510 participants for oropharyngeal cancer, 361,194 participants for lymphoma and 463,010 participants for cervical cancer.

Covariates for multivariable MR analyses were included: BMI (681,275 participants) [32], educational attainment (766,345 participants) [33], intelligence (269,867 participants) [34], average total household income before tax (income, 397,751 participants) [25], cigarettes per day (smoking, 337,334 participants) [35] and alcoholic drinks per week (alcohol consumption, 335,394 participants) [35]. All data came from the European population. Detailed information on data can be found in Table 1.

At the beginning of our study design, 24 site-specific cancers were considered. Overall cancer and 12 site-specific cancers were included, but another 12 types of cancer were not included due to insufficient SNPs (stomach cancer, pancreatic cancer, esophagus cancer, kidney cancer, liver cancer, biliary tract cancer, head and neck cancer, bladder cancer, testis cancer, brain cancer, multiple myeloma and bone cancer). In Table S1, we provide detailed information on cancers that failed to perform the MR study.

### 2.3. Selection of Instrumental Variables

Appropriate SNPs used as IVs must be robustly associated with cancers (*p* < 5 × 10^−8^). To ensure independence, SNPs were restricted by low linkage disequilibrium (LD, r^2^ < 0.001, window size = 10,000 kb) using clumping [14,36]. We excluded palindromic SNPs whose minor allele frequency (MAF) was less than 0.42. In addition, we calculated F-statistics for SNPs to measure instrumental strength. SNPs with an F-statistic less than 10 were removed [37]. Detailed information on selected SNPs can be found in Table S2. One SNP (rs11571818) of squamous cell lung cancer was removed (F-statistic: 7.94).

### 2.4. Statistical Analysis

In the univariable MR analysis, the IVW analysis was chosen as the primary approach to estimate the causal effects of cancers on COVID-19 outcomes [15,38]. We added the MR-Egger regression [39], weighted median [40], weighted mode [41] and MR pleiotropy residual sum and outlier (MR-PRESSO) [42] methods as supplements to sensitivity analyses. The third assumption (that IVs cannot affect the outcomes of COVID-19 through alternative pathways) was defined as independence from pleiotropy [14]. When performing MR analysis, results may be inaccurate due to the pleiotropy of these SNPs [36]. Therefore, we evaluated the potential pleiotropy via the MR-PRESSO approach. The MR-PRESSO approach could identify and correct possible outliers and estimate causal effects [42]. We evaluated the heterogeneity by Cochran’s Q test. The fixed-effect model was used if no heterogeneity was observed (*p* < 0.1); otherwise, a random-effect model was applied. In addition, we used the “leave-one-out” validation to determine whether a single SNP had a significant independent influence on the MR estimation.

We applied the random-effect IVW method to assess the causal effects of cancers on COVID-19 outcomes for the multivariable MR analyses, after controlling BMI, educational attainment, intelligence, smoking and alcohol consumption. Given the number of cancers and COVID-19 outcomes considered, a two-sided *p*-value using the Bonferroni correction (0.0033, 0.05/15 cancers) was used. 0.0033 < *p* < 0.05 was regarded as suggestive evidence for a potential association. The *β* (*β* = lnOR; OR, odds ratio) and its SE (standard error) were calculated to reflect effect sizes. All statistical analyses were conducted in R v4.0.1 (R Foundation, Vienna, Austria) with the packages “TwoSampleMR” and “MRPRESSO” [42,43].

## 3. Results

### 3.1. Cancers and COVID-19 Severity

A total of 1,388,342 participants (5101 very serious respiratory confirmed patients and 1,383,241 controls) were included for COVID-19 severity. Severe COVID-19 cases were defined as patients who died or required respiratory support with COVID-19 infection. The effects of each SNP in cancers on COVID-19 severity can be found in Figure S1. There was significant heterogeneity in the IVW analyses of prostate cancer (*p* = 0.07), ovarian cancer (*p* < 0.001), melanoma (*p* = 0.01) and cervical cancer (*p* = 0.08) (Table 2). Hence, we performed the random-effect model in their IVW analyses. IVW analysis suggested no causal effect of overall cancer (*p* = 0.34), lung cancer (*p* = 0.60), squamous cell lung cancer (*p* = 0.66), breast cancer (*p* = 0.43), ER+ breast cancer (*p* = 0.79), ER− breast cancer (*p* = 0.66), endometrial cancer (*p* = 0.79), prostate cancer *(p* = 0.54), thyroid cancer (*p* = 0.70), ovarian cancer (*p* = 0.62), melanoma (*p* = 0.79), small bowel cancer (*p* = 0.09), colorectal cancer (*p* = 0.85), oropharyngeal cancer (*p* = 0.31), lymphoma (*p* = 0.51) or cervical cancer (*p* = 0.25) on the COVID-19 severity (Table 2).

In the sensitivity analyses, the MR-PRESSO test indicated significant horizontal pleiotropy in the analyses of prostate cancer (*p* = 0.03) and ovarian cancer (*p* = 0.01) (Table 2). After removing the horizontal pleiotropy SNPs (rs12139208 for prostate cancer; rs115478735 for ovarian cancer), MR-PRESSO analysis suggested that prostate cancer and ovarian cancer had no causal association with COVID-19 severity (*p* = 0.60; *p* = 0.96). Although horizontal pleiotropy was observed in melanoma (*p* = 0.02), it showed no significant outlier. We conducted the “leave-one-out” analysis and found no potential SNP significantly biasing the results (Figure S4). Taken together, sensitivity analyses (MR-Egger, weighted median, weighted mode and MR-PRESSO) revealed that cancers had no causal association with COVID-19 severity (Table 2). Results of multivariable MR analyses also supported our findings (Table S3).

### 3.2. Cancers and COVID-19 Hospitalization

COVID-19 hospitalization analysis contained 1,887,658 participants (9986 hospitalization patients and 1,877,672 controls). Figure S2 represents the effects of each SNP in cancers on COVID-19 hospitalization. Significant heterogeneity was observed in the analyses of thyroid cancer (*p* = 0.06), ovarian cancer (*p* < 0.001) and cervical cancer (*p* = 0.04) (Table 3). The random-effect model was subsequently applied. IVW analysis revealed no causal effect of overall cancer (*p* = 0.42), lung cancer (*p* = 0.37), squamous cell lung cancer (*p* = 0.66), breast cancer (*p* = 0.74), ER+ breast cancer (*p* = 0.51), ER− breast cancer (*p* = 0.93), endometrial cancer (*p* = 0.24), prostate cancer (*p* = 0.17), thyroid cancer (*p* = 0.80), ovarian cancer (*p* = 0.96), melanoma (*p* = 0.45), small bowel cancer (*p* = 0.08), colorectal cancer (*p* = 0.79) or cervical cancer (*p* = 0.32) on COVID-19 hospitalization (Table 3).

In the sensitivity analyses, the MR-PRESSO test indicated significant horizontal pleiotropy in the analysis of ovarian cancer (*p* < 0.001; Table 3). After removing the horizontal pleiotropy SNPs (rs115478735 and rs71238846), ovarian cancer was still not significantly associated with COVID-19 hospitalization in the MR-PRESSO analysis (*p* = 0.78). The “leave-one-out” analysis showed no outliers (Figure S5). Although MR-Egger test indicated a potential association of thyroid cancer with COVID-19 hospitalization (*p* = 0.04), estimates in the three analyses (weighted median, weighted mode and MR-PRESSO; Table 3) directionally matched the result of IVW analysis. In the multivariable MR analyses (Table S4), potential association with COVID-19 hospitalization was observed in overall cancer (*p* = 0.01) and prostate cancer (*p* = 0.046) when adjusting for education attainment. A significant association was also found in small bowel cancer (*p* = 0.047) when adjusting for smoking. However, the associations of overall cancer, prostate cancer and small bowel cancer with COVID-19 hospitalization could not be replicated when intelligence (*p* = 0.18; *p* = 0.10; *p* = 0.23), income (*p* = 0.28; *p* = 0.06; *p* = 0.28) and alcohol consumption (*p* = 0.58; *p* = 0.11; *p* = 0.43) were adjusted (Table S4). Therefore, there was no strong evidence for a causal association of overall cancer, prostate cancer, or small bowel cancer with COVID-19 hospitalization.

### 3.3. Cancers and COVID-19 Susceptibility

A total of 1,683,768 participants (38,984 infection patients and 1,644,784 controls) were included for COVID-19 susceptibility. Figure S3 shows the effects of each SNP in cancers on COVID-19 susceptibility. There was significant heterogeneity in the IVW analyses of ER+ breast cancer (*p* = 0.02), prostate cancer (*p* = 0.06), thyroid cancer (*p* = 0.06), ovarian cancer (*p* < 0.001), melanoma (*p* = 0.05) and cervical cancer (*p* < 0.001) (Table 4). Thus, we performed the random-effect model for their IVW analyses. IVW analysis suggested no causal effect of overall cancer (*p* = 0.69), lung cancer (*p* = 0.96), squamous cell lung cancer (*p* = 0.08), breast cancer (*p* = 0.43), ER+ breast cancer (*p* = 0.30), ER− breast cancer (*p* = 0.18), endometrial cancer (*p* = 0.83), prostate cancer (*p* = 0.58), thyroid cancer (*p* = 0.28), ovarian cancer (*p* = 0.93), melanoma (*p* = 0.82), small bowel cancer (*p* = 0.19), colorectal cancer (*p* = 0.30), oropharyngeal cancer (*p* = 0.80), lymphoma (*p* = 0.37) or cervical cancer (*p* = 0.68) on COVID-19 susceptibility (Table 4).

In the sensitivity analyses, the MR-PRESSO test indicated significant horizontal pleiotropy in the analyses of ER+ breast cancer (*p* = 0.02) and ovarian cancer (*p* < 0.001) (Table 4). After removing the horizontal pleiotropy SNPs (rs4971059 for ER+ breast cancer, rs115478735 and rs71238846 for ovarian cancer), MR-PRESSO analysis suggested that ER+ breast cancer and ovarian cancer still had no causal association with COVID-19 susceptibility (*p* = 0.50; *p* = 0.39). The “leave-one-out” plot showed one potential instrumental outlier (rs6983267) for colorectal cancer (Figure S6M). However, results of multivariable MR analyses (Table S5) supported colorectal cancer having no significant causal effect on COVID-19 susceptibility. In summary, there was no strong evidence for a causal association of overall cancer or twelve site-specific cancers with COVID-19 susceptibility.

## 4. Discussion

During the COVID-19 pandemic, healthcare resources are extremely scarce, and there is an urgent need to allocate healthcare resources rationally [44]. Identifying individuals who are vulnerable to SARS-CoV-2 and those who are prone to severe illness is of great significance for optimizing the allocation of healthcare resources. Epidemiological studies have suggested that cancer is an independent adverse prognostic factor on COVID-19 outcomes [10,45], but causality has not been assessed. We used the MR analysis to evaluate the causal effects of overall cancer and twelve site-specific cancers (lung cancer, breast cancer, endometrial cancer, prostate cancer, thyroid cancer, ovarian cancer, melanoma, small bowel cancer, colorectal cancer, oropharyngeal cancer, lymphoma and cervical cancer) on COVID-19 outcomes (severity, hospitalization and susceptibility). The MR study on extensive international genetic consortia provided no strong evidence to support the causal role of cancer in COVID-19 development.

MR leverages the random allocation of genetic variants at conception, independently of confounders, to identify the causal effects that are substantially less confounded and not vulnerable to reverse causation [14,15]. We used SNPs as instrumental variables to conduct the MR study. Five analyses (IVW, MR-Egger, weighted median, weighted mode and MR-PRESSO) suggested no causal effect of overall cancer or twelve site-specific cancers on COVID-19 outcomes. Multivariate MR estimates (adjusted for BMI, education attainment, intelligence, income, smoking and alcohol consumption) were consistent with the results of five analyses. Besides UK biobank, we introduced other data to verify the results of this study (Table S6). Taken together, we concluded that cancers might have no causal effect on increasing COVID-19 risk, and these results were robust. 

Although many studies have generally shown positive correlations of cancers with the risk of COVID-19 [10,45,46,47], some subsequent findings are inconsistent with previous studies. No statistically significant difference was found between the severe and non-severe COVID-19 group of cancer among non-Asian patients [48]. A meta-analysis involving 46,499 patients revealed that cancer was not a risk factor for COVID-19 death in elderly patients [49]. Moreover, another meta-analysis showed that colorectal cancer patients are not significantly susceptible to SARS-CoV-2 in the global population [50]. Interestingly, a recent meta-analysis suggested that no significantly increased risk of severe illness of COVID-19 was observed in patients with lung or stage IV cancer [51]. The conflicting results indicated that cancers might not be causally associated with COVID-19 outcomes.

Risk factors may be correlated with COVID-19 outcomes, but not as a causal association. Previous MR studies have shown that many traditional risk factors have no causal association with COVID-19 outcomes, such as decreased lung function, chronic obstructive pulmonary disease, blood pressure, type 2 diabetes, chronic kidney disease, coronary artery disease, stroke and nonalcoholic fatty liver disease [52,53,54,55]. However, BMI has been robustly correlated and causally associated with COVID-19 outcomes [54]. In fact, many studies have shown that there is a significant difference in the age distribution between cancer and non-cancer patients infected with COVID-19 [46,47,56]. In addition, the mortality rate of COVID-19 in cancer patients appears to be mainly determined by age, gender and comorbidities [57,58]. Therefore, the reported correlations of risk factors with COVID-19 outcomes might be confounded in observational studies, possibly due to confounders including BMI, age and gender. Notably, cancer patients should remain a key focus during the COVID-19 pandemic. Risk factors were clinically helpful in identifying critically ill patients of COVID-19, even without a causal association.

MR design is less confounding than observational study, but limitations of this MR study need to be acknowledged. First, some types of cancer—such as stomach cancer, pancreatic cancer, liver cancer and brain cancer—were not included in the study because of insufficient SNPs (Table S1). Potential causality for COVID-19 outcomes might be observed in other types of cancer. Second, our results are primarily based on participants of European descent, to reduce racial influence. The findings of our MR study might not apply to other ethnic groups. With racial minorities disproportionately affected by the pandemic [59,60], reliable research on non-European ancestry is urgently needed. Third, gender-specific cancers (breast cancer, endometrial cancer, prostate cancer, ovarian cancer and cervical cancer) were included. Although the original researchers have adjusted the COVID-19 GWAS data for gender, it might have a confounded impact. Lastly, data were extracted from vast genetic epidemiological networks, but our study failed to detect minimal effects.

## 5. Conclusions

Overall, we used MR analysis to evaluate the causal effects of overall cancer and twelve site-specific cancers on COVID-19 severity, hospitalization and susceptibility. Results of the MR study did not suggest strong evidence to support the causal associations of any examined cancer with COVID-19 outcomes. Previous observational correlations of cancers with COVID-19 outcomes were likely confounded. More large-scale epidemiological studies are needed to validate our findings.

## Figures and Tables

**Table 1:** Summary of the included data.

Variable		Cases	Controls	Sample Size	Year	GWAS ID
COVID-19	COVID-19 susceptibility	38,984	1,644,784	1,683,768	2021	-
	COVID-19 hospitalization	9986	1,877,672	1,887,658	2021	-
	COVID-19 severity	5101	1,383,241	1,388,342	2021	-
Cancer	Overall cancer	26,576	309,696	336,272	2017	ukb-a-307
	Lung cancer	11,348	15,861	27,209	2014	ieu-a-966
	Squamous cell lung cancer	3275	15,038	18,313	2014	ieu-a-967
	Breast cancer	122,977	105,974	228,951	2017	ieu-a-1126
	ER+ Breast cancer	69,501	105,974	175,475	2017	ieu-a-1127
	ER− Breast cancer	21,468	105,974	127,442	2017	ieu-a-1128
	Ovarian cancer	25,509	40,941	66,450	2017	ieu-a-1120
	Endometrial cancer	12,906	108,979	121,885	2018	ebi-a-GCST006464
	Prostate cancer	79,148	61,106	140,254	2018	ieu-b-85
	Thyroid cancer	649	431	1080	2013	ieu-a-1082
	Melanoma	3751	372,016	375,767	2021	ieu-b-4969
	Small bowel cancer	156	337,003	337,159	2017	ukb-a-56
	Colorectal cancer	5657	372,016	377,673	2021	ieu-b-4965
	Oropharyngeal cancer	494	372,016	372,510	2021	ieu-b-4968
	Lymphoma	1752	359,442	361,194	2018	ukb-d-C_LYMPHOMA
	Cervical cancer	3175	459,835	463,010	2018	ukb-b-918
Covariates	BMI	-	-	681,275	2018	ieu-b-40
	Educational attainment	-	-	766,345	2018	ieu-a-1239
	Intelligence	-	-	269,867	2018	ebi-a-GCST006250
	Income	-	-	397,751	2018	ukb-b-7408
	Smoking	-	-	337,334	2019	ieu-b-25
	Alcohol consumption	-	-	335,394	2019	ieu-b-73

**Table 2:** Causal effects of cancers on COVID-19 severity estimated by univariable Mendelian randomization.

Cancer Types	No. of SNPs	IVW			MR-Egger			Weighted Median			Weighted Mode			MR-PRESSO			Heterogeneity	Pleiotropy
		*β*	SE	*p*	*β*	SE	*p*	*β*	SE	*p*	*β*	SE	*p*	*β*	SE	*p*	*p*	*p*
Overall cancer	4	−3.44	3.61	0.34	112.35	104.87	0.40	−1.63	4.25	0.70	0.77	6.26	0.91	−3.44	4.11	0.46	0.27	0.33
Lung cancer	5	0.03	0.07	0.60	0.16	0.25	0.57	0.06	0.08	0.45	0.08	0.08	0.38	0.03	0.06	0.59	0.53	0.58
Squamous cell lung cancer	2	−0.05	0.12	0.66	-	-	-	-	-	-	-	-	-	-	-	-	-	-
Breast cancer	109	0.04	0.05	0.43	0.05	0.11	0.61	0.07	0.08	0.39	0.05	0.09	0.56	0.05	0.05	0.31	0.35	0.23
ER+ Breast cancer	81	−0.01	0.05	0.79	0.04	0.11	0.70	0.09	0.07	0.20	0.10	0.08	0.24	0.0001	0.05	1.00	0.13	0.10
ER− Breast cancer	27	0.03	0.06	0.66	−0.20	0.17	0.25	−0.03	0.09	0.73	−0.07	0.11	0.56	0.03	0.06	0.63	0.29	0.35
Endometrial cancer	12	0.02	0.09	0.79	−0.08	0.36	0.84	0.01	0.13	0.96	0.31	0.26	0.26	0.02	0.09	0.80	0.36	0.38
Prostate cancer	91	−0.02	0.04	0.54	−0.15	0.09	0.11	−0.02	0.07	0.74	−0.07	0.07	0.30	−0.02	0.04	0.60	0.07 *	0.03 ^$^
Thyroid cancer	249	−0.001	0.002	0.70	−0.003	0.003	0.29	−0.003	0.003	0.32	−0.004	0.004	0.27	−0.001	0.002	0.70	0.33	0.33
Ovarian cancer	9	0.08	0.16	0.62	−0.08	0.41	0.84	0.06	0.11	0.57	0.11	0.12	0.40	0.004	0.10	0.96	<0.001 *	0.01 ^$^
Melanoma	6	−3.45	12.83	0.79	−6.56	39.71	0.88	−10.76	10.13	0.29	−17.50	11.33	0.18	−3.45	12.83	0.80	0.01 *	0.02 ^$^
Small bowel cancer	5	84.03	48.79	0.09	−11.30	156.92	0.95	43.10	61.67	0.48	40.60	79.89	0.64	84.03	31.59	0.06	0.79	0.76
Colorectal cancer	7	−1.05	5.44	0.85	1.45	19.05	0.94	−1.15	6.94	0.87	−1.76	8.85	0.85	−1.05	3.46	0.77	0.88	0.89
Oropharyngeal cancer	2	−52.19	51.79	0.31	-	-	-	-	-	-	-	-	-	-	-	-	0.95	-
Lymphoma	2	−15.04	22.77	0.51	-	-	-	-	-	-	-	-	-	-	-	-	0.95	-
Cervical cancer	2	−28.58	24.70	0.25	-	-	-	-	-	-	-	-	-	-	-	-	0.08 *	-

* Significant heterogeneity (*p* < 0.1); ^$^ significant horizontal pleiotropy (*p* < 0.05).

**Table 3:** Causal effects of cancers on COVID-19 hospitalization estimated by univariable Mendelian randomization.

Cancer Types	No. of SNPs	IVW			MR-Egger			Weighted Median			Weighted Mode			MR-PRESSO			Heterogeneity	Pleiotropy
		*β*	SE	*p*	*β*	SE	*p*	*β*	SE	*p*	*β*	SE	*p*	*β*	SE	*p*	*p*	*p*
Overall cancer	4	−1.86	2.32	0.42	22.70	61.39	0.75	−2.32	2.73	0.40	−2.83	3.81	0.51	−1.86	1.65	0.34	0.68	0.71
Lung cancer	4	0.04	0.05	0.37	0.29	0.20	0.29	0.06	0.05	0.23	0.07	0.06	0.31	0.04	0.03	0.31	0.66	0.63
Squamous cell lung cancer	2	−0.04	0.08	0.66	-	-	-	-	-	-	-	-	-	-	-	-	0.99	-
Breast cancer	106	0.01	0.03	0.74	-0.003	0.07	0.97	0.01	0.05	0.80	0.02	0.06	0.70	0.02	0.03	0.60	0.20	0.13
ER+ Breast cancer	79	−0.02	0.03	0.51	0.04	0.07	0.56	−0.02	0.05	0.71	0.02	0.05	0.77	−0.01	0.03	0.70	0.36	0.22
ER− Breast cancer	25	−0.004	0.04	0.93	0.03	0.12	0.83	−0.03	0.06	0.63	−0.05	0.09	0.59	-0.005	0.04	0.90	0.58	0.60
Endometrial cancer	12	0.06	0.05	0.24	0.43	0.21	0.07	0.07	0.08	0.34	0.03	0.11	0.77	0.06	0.06	0.28	0.36	0.37
Prostate cancer	90	0.04	0.03	0.17	−0.01	0.06	0.85	0.06	0.04	0.15	0.05	0.05	0.28	0.04	0.03	0.16	0.12	0.07
Thyroid cancer	246	−0.0003	0.001	0.80	−0.004	0.002	0.04 ^#^	−0.0005	0.002	0.79	0.0002	0.002	0.94	0.0003	0.0005	0.63	0.06 *	0.08
Ovarian cancer	9	0.01	0.11	0.96	−0.20	0.28	0.52	0.01	0.08	0.94	0.01	0.08	0.85	0.01	0.04	0.78	<0.001 *	<0.001 ^$^
Melanoma	6	−3.52	4.62	0.45	−3.62	16.93	0.84	−10.08	6.00	0.09	−11.14	7.97	0.22	−3.52	5.60	0.56	0.20	0.26
Small bowel cancer	2	78.90	45.54	0.08	-	-	-	-	-	-	-	-	-	-	-	-	0.83	-
Colorectal cancer	7	0.99	3.70	0.79	7.67	13.80	0.60	2.91	4.62	0.53	4.04	5.77	0.51	0.99	2.48	0.70	0.85	0.85
Oropharyngeal cancer	-	-	-	-	-	-	-	-	-	-	-	-	-	-	-	-	-	-
Lymphoma	-	-	-	-	-	-	-	-	-	-	-	-	-	-	-	-	-	-
Cervical cancer	2	−19.95	20.17	0.32	-	-	-	-	-	-	-	-	-	-	-	-	0.04 *	-

* Significant heterogeneity (*p* < 0.1); ^$^ significant horizontal pleiotropy (*p* < 0.05); ^#^ potential association (*p* < 0.05).

**Table 4:** Causal effects of cancers on COVID-19 susceptibility estimated by univariable Mendelian randomization.

Cancer Types	No. of SNPs	IVW			MR-Egger			Weighted Median			Weighted Mode			MR-PRESSO			Heterogeneity	Pleiotropy
		*β*	SE	*p*	*β*	SE	*p*	*β*	SE	*p*	*β*	SE	*p*	*β*	SE	*p*	*p*	*p*
Overall cancer	4	0.47	1.15	0.69	−3.29	42.93	0.95	−0.62	1.38	0.65	−0.78	1.95	0.72	0.47	1.32	0.75	0.27	0.35
Lung cancer	5	0.001	0.02	0.96	0.03	0.08	0.77	0.02	0.03	0.54	0.02	0.03	0.56	0.00	0.02	0.95	0.54	0.59
Squamous cell lung cancer	2	−0.07	0.04	0.08	-	-	-	-	-	-	-	-	-	-	-	-	0.77	-
Breast cancer	109	−0.01	0.02	0.43	−0.01	0.04	0.85	−0.02	0.03	0.44	−0.03	0.03	0.43	−0.01	0.02	0.57	0.26	0.23
ER+ Breast cancer	81	−0.02	0.02	0.30	0.01	0.04	0.78	−0.02	0.02	0.38	−0.03	0.03	0.36	−0.01	0.02	0.50	0.02 *	0.02 ^$^
ER− Breast cancer	27	−0.03	0.02	0.18	−0.08	0.06	0.19	−0.03	0.03	0.27	−0.05	0.04	0.24	−0.03	0.02	0.22	0.39	0.46
Endometrial cancer	12	−0.01	0.03	0.83	0.04	0.11	0.69	−0.01	0.03	0.69	−0.02	0.05	0.69	−0.01	0.02	0.76	0.92	0.91
Prostate cancer	91	−0.01	0.01	0.58	−0.04	0.03	0.16	−0.03	0.02	0.16	−0.03	0.02	0.17	−0.01	0.01	0.67	0.06 *	0.05
Thyroid cancer	248	0.001	0.0006	0.28	−0.000001	0.001	1.00	0.0001	0.0009	0.94	0.0004	0.001	0.71	0.0006	0.0006	0.28	0.06 *	0.05
Ovarian cancer	9	−0.01	0.09	0.93	−0.14	0.22	0.54	−0.01	0.04	0.74	0.02	0.04	0.68	−0.03	0.03	0.39	<0.001 *	<0.001 ^$^
Melanoma	6	0.76	3.28	0.82	9.84	8.38	0.31	−1.10	2.97	0.71	−1.11	3.78	0.78	0.76	3.28	0.83	0.05 *	0.05
Small bowel cancer	4	23.09	17.71	0.19	148.10	80.98	0.21	5.85	20.89	0.78	−2.59	29.45	0.94	23.09	19.36	0.32	0.31	0.37
Colorectal cancer	7	1.96	1.88	0.30	8.71	8.86	0.37	1.85	2.64	0.48	6.56	6.09	0.32	1.96	2.31	0.43	0.17	0.16
Oropharyngeal cancer	2	−3.82	15.14	0.80	-	-	-	-	-	-	-	-	-	-	-	-	0.77	-
Lymphoma	2	−6.05	6.79	0.37	-	-	-	-	-	-	-	-	-	-	-	-	0.47	-
Cervical cancer	2	−7.01	17.21	0.68	-	-	-	-	-	-	-	-	-	-	-	-	<0.001 *	-

* Significant heterogeneity (*p* < 0.1); ^$^ significant horizontal pleiotropy (*p* < 0.05).

## Data Availability

All relevant data are within the paper.

## Supplementary Materials

The following supporting information can be downloaded at https://www.mdpi.com/article/10.3390/cancers14092086/s1. Figure S1: The effects of each SNP in cancers on COVID-19 severity; Figure S2: The effects of each SNP in cancers on COVID-19 hospitalization; Figure S3: The effects of each SNP in cancers on COVID-19 susceptibility; Figure S4: The leave-one-out sensitivity analysis of the causal effects of cancers on COVID-19 severity; Figure S5: The leave-one-out sensitivity analysis of the causal effects of cancers on COVID-19 hospitalization; Figure S6: The leave-one-out sensitivity analysis of the causal effects of cancers on COVID-19 susceptibility; Table S1: Excluded cancers with insufficient SNPs; Table S2: Selected SNPs of included cancers; Table S3: Causal effects of cancers on COVID-19 severity estimated by multivariable Mendelian randomization; Table S4: Causal effects of cancers on COVID-19 hospitalization estimated by multivariable Mendelian randomization; Table S5: Causal effects of cancers on COVID-19 susceptibility estimated by multivariable Mendelian randomization; Table S6: Verification of the causal effects of cancers on COVID-19 outcomes.
