# Supplementary material for: Cancers and COVID-19 Risk: A Mendelian Randomization Study
Source: Cancers (Basel). 2022 Apr 22;14(9):2086. doi: 10.3390/cancers14092086 (PMC9099868; doi:10.3390/cancers14092086)
Supplement: Supplementary file 1 [file cancers-14-02086-s001.zip › cancers-1653310 Supplementary Materials.pdf]

Supplementary Materials

Cancers and COVID-19 Risk: A Mendelian Randomization Study

Zengbin Li [1], Yudong Wei [1], Guixian Zhu [1], Mengjie Wang [1] and Lei Zhang [1,2,3,4,*]

[1] China-Australia Joint Research Center for Infectious Diseases, School of Public Health, Xi'an Jiaotong University Health Science Center, Xi'an 710061, China; zengbinli@stu.xjtu.edu.cn (Z.L.); weiyudong@stu.xjtu.edu.cn (Y.W.); xianxianshell@stu.xjtu.edu.cn (G.Z.); mjwang0211@stu.xjtu.edu.cn (M.W.)
[2] Melbourne Sexual Health Centre, Alfred Health, Melbourne, VIC 3053, Australia
[3] Central Clinical School, Faculty of Medicine, Nursing and Health Sciences, Monash University, Melbourne, VIC 3800, Australia
[4] Department of Epidemiology and Biostatistics, College of Public Health, Zhengzhou University, Zhengzhou 450001, China
*Correspondence: lei.zhang1@monash.edu; Tel.: +86-29-82655135

Table S1. Excluded cancers with insufficient SNPs

	Cancer		GWAS ID	Year	Cases	Controls
Discovery	Stomach cancer	Benign neoplasm: Stomach	finn-b-CD2_BENIGN_STOMACH	2021	1252	217,540
		Malignant neoplasm of stomach	finn-b-C3_STOMACH_EXALLC	2021	633	174,006
	Pancreatic cancer	Pancreatic cancer	ieu-a-822	2009	1896	1939
		Pancreas benign neoplasm	finn-b-CD2_BENIGN_PANCREAS_EXALLC	2021	163	180,666
	Oesophagus cancer	Malignant neoplasm of oesophagus	finn-b-C3_OESOPHAGUS	2021	144	218,648
		Benign neoplasm: Oesophagus	finn-b-CD2_BENIGN_OESOPHAGUS	2021	232	218,560
	Kidney cancer	Malignant neoplasm of kidney, except renal pelvis	ukb-b-1316	2018	1114	461,896
		Malignant neoplasm of kidney, except renal pelvis	finn-b-C3_KIDNEY_NOTRENALPELVIS_EXALLC	2021	971	174,006
		Benign neoplasm: Kidney	finn-b-CD2_BENIGN_KIDNEY_EXALLC	2021	381	180,506
		Malignant neoplasm of kidney, except renal pelvis	finn-b-C3_KIDNEY_NOTRENALPELVIS_EXALLC	2021	971	217,821
	Liver cancer	Secondary malignant neoplasm of liver	ukb-b-16713	2018	1139	461,871
		Liver & bile duct cancer	ieu-b-4915	2021	350	372,016
		Liver cell carcinoma	ieu-b-4953	2021	168	372,016
		Benign neoplasm: Liver	finn-b-CD2_BENIGN_LIVER	2021	232	218,560
		Benign neoplasm: Liver/bile ducts	finn-b-CD2_BENIGN_LIVE_BILE	2021	264	218,528
		Malignant neoplasm of liver and intrahepatic bile ducts	finn-b-C3_LIVER_INTRAHEPATIC_BILE_DUCTS	2021	304	218,488
	Biliary tract cancer	Malignant neoplasm of other and unspecified parts of biliary tract	finn-b-C3_BILIARY_TRACT	2021	109	218,683
		Gallbladder cancer	ieu-a-1057	2012	41	866
	Head and neck cancer	Head and neck cancer	ieu-b-4912	2021	1106	372,016
		Benign lipomatous neoplasm of skin and subcutaneous tissue of head, face and neck	finn-b-CD2_BENIGN_LIPO_SKIN_HEADFACENECK	2021	915	217,877

		Benign neoplasm: Connective and other soft tissue of head, face and neck	finn-b-CD2_BENIGN_CONNSOFT_HEADFACETRUNK	2021	262	218,530
	Bladder cancer	Bladder cancer	ukb-b-8193	2018	1101	461,832
		Malignant neoplasm of bladder	ukb-d-C67	2018	1554	359,640
		Benign neoplasm: Bladder	finn-b-CD2_BENIGN_BLADDER	2021	109	218,683
		Malignant neoplasm of bladder	finn-b-C3_BLADDER	2021	1115	217,677
	Testis cancer	Malignant neoplasm of testis	finn-b-C3_TESTIS	2021	199	95,014
	Brain cancer	Brain cancer	ieu-b-4875	2021	606	372,016
		Malignant neoplasm of brain	finn-b-C3_BRAIN	2021	464	218,328
		Malignant neoplasm of eye, brain and central nervous system	finn-b-C3_EYE_BRAIN_NEURO	2021	1446	217,346
		Benign neoplasm: Brain, supratentorial	finn-b-CD2_BENIGN_BRAIN_SUPRATENT	2021	218	218,574
	Multiple myeloma	Multiple myeloma	ieu-b-4957	2021	601	372,016
	Bone cancer	Malignant neoplasm of bone and articular cartilage	finn-b-C3_BONE_CARTILAGE_EXALLC	2021	119	174,006
		Benign neoplasm: Short bones of lower limb	finn-b-CD2_BENIGN_BONE_SHORT_LOWER	2021	128	218,664
		Benign neoplasm: Scapula and long bones of upper limb	finn-b-CD2_BENIGN_BONE_SCAPULA_LONG_UPPER	2021	125	218,667
		Benign neoplasm: Long bones of lower limb	finn-b-CD2_BENIGN_BONE_LONG_LOWER	2021	443	218,349
		Benign neoplasm: Bones of skull and face	finn-b-CD2_BENIGN_BONE_SKULL_FACE	2021	120	218,672
Validation	Lung cancer	Non-small cell lung cancer	finn-b-C3_LUNG_NONSMALL	2021	1627	217,165
		Lung adenocarcinoma	ieu-a-965	2014	3442	14,894
		Non-small cell lung cancer, adenocarcinoma	finn-b-C3_NSCLC_ADENO	2021	571	218,221
		Non-small cell lung cancer, squamous	finn-b-C3_NSCLC_SQUAM_EXALLC	2021	365	174,004

Ovarian cancer	Ovarian cancer	ieu-b-4963	2021	1218	198,523
Endometrial cancer	Uterine/endometrial cancer	ukb-b-13545	2018	1151	461,782
Thyroid cancer	Benign neoplasm of thyroid gland	finn-b-CD2_BENIGN_THYROID	2021	455	218,337
Melanoma	Malignant melanoma of skin	finn-b-C3_MELANOMA_SKIN	2021	98	218,694
	Melanoma in situ	finn-b-CD2_INSITU_MELANOMA	2021	393	218,399
Colorectal cancer	Colorectal cancer	finn-b-C3_COLORECTAL	2021	3022	215,770
	Malignant neoplasm of colon	finn-b-C3_COLON	2021	1803	216,989
Lymphoma	Hodgkin lymphoma	finn-b-CD2_HODGKIN_LYMPHOMA	2021	369	218,423
	Other and unspecified types of non-Hodgkin lymphoma	finn-b-CD2_NONHODGKIN_NAS	2021	533	218,259
	Diffuse large B-cell lymphoma	finn-b-C3_DLBCL	2021	209	218,583
	Mature T/NK-cell lymphomas	finn-b-CD2_TNK_LYMPHOMA	2021	150	218,642
	Non-follicular lymphoma	finn-b-CD2_NONFOLLICULAR_LYMPHOMA	2021	1283	217,509
	Follicular lymphoma	finn-b-CD2_FOLLICULAR_LYMPHOMA_EXALLC	2021	522	180,756
Oropharyngeal cancer	Oropharyngeal cancer	ieu-b-96	2016	1090	2928
Cervix cancer	cin/pre-cancer cells cervix	ukb-b-17507	2018	1604	461,329
	Carcinoma in situ of cervix uteri	finn-b-CD2_INSITU_CERVIX_UTERI	2021	298	123,281
	Carcinoma in situ of cervix uteri, exocervix	finn-b-CD2_INSITU_CERVIX_UTERI_EXOCERVIX	2021	107	123,472

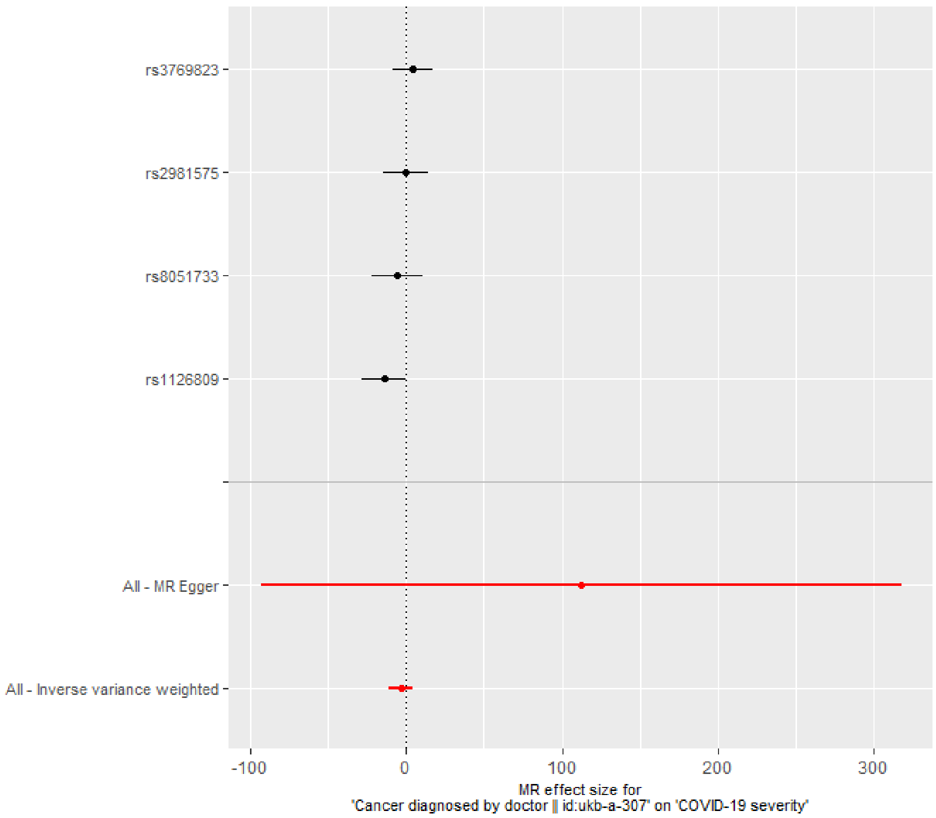

Figure S1A. The effect of each SNP in overall cancer on COVID-19 severity.

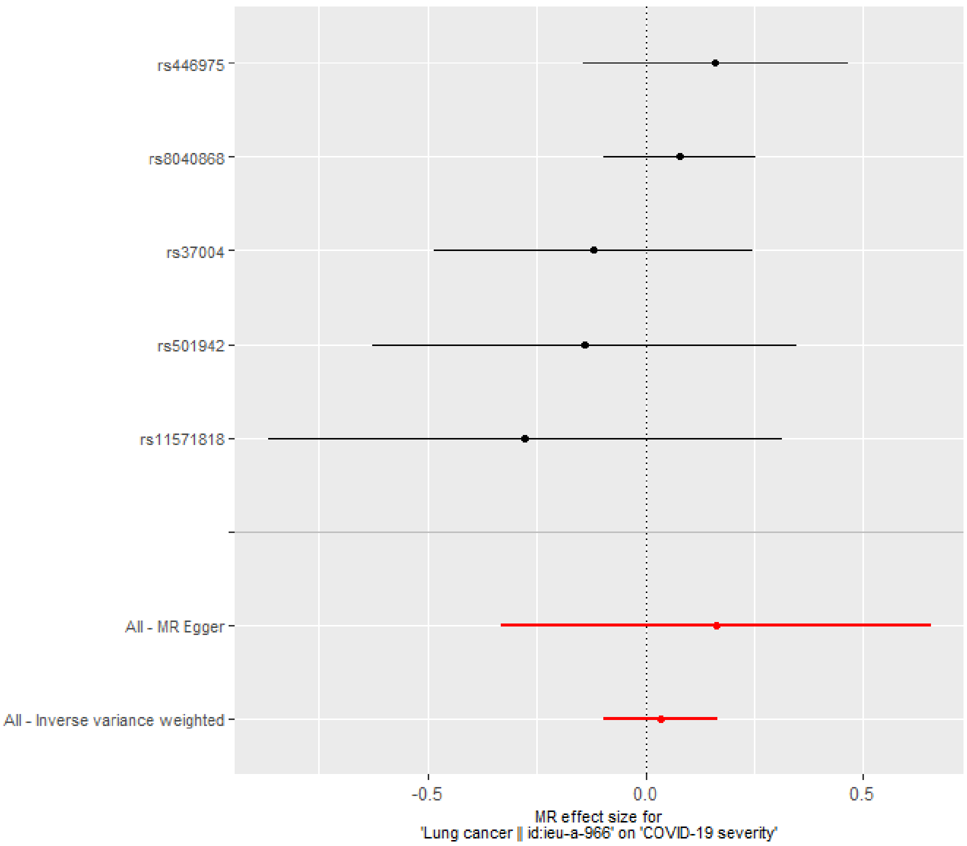

Figure S1B. The effect of each SNP in lung cancer on COVID-19 severity.

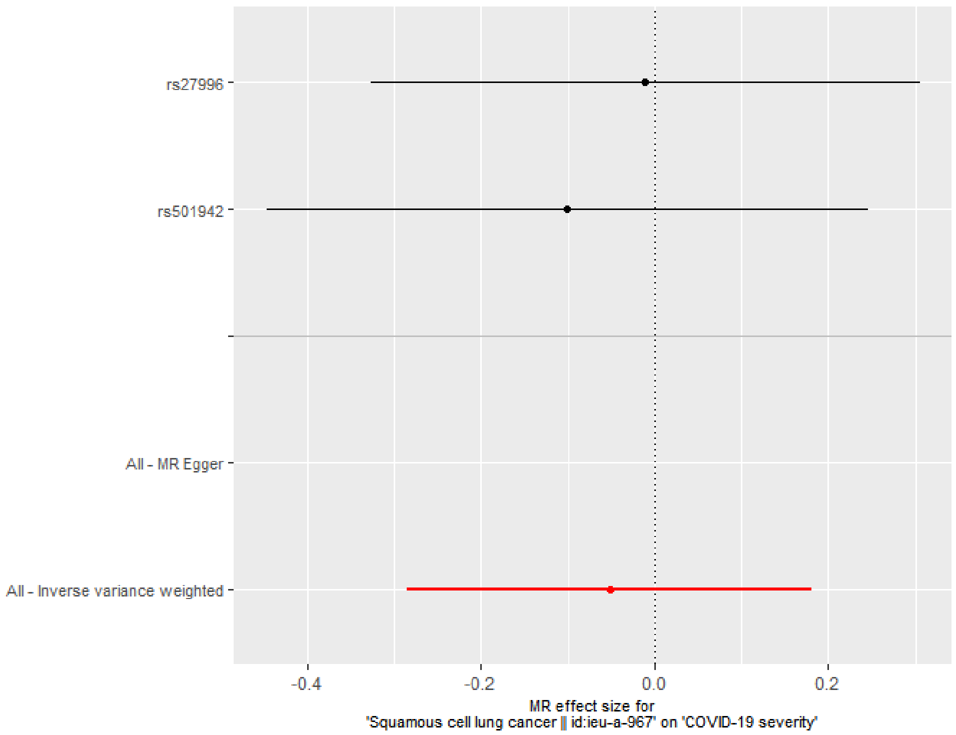

Figure S1C. The effect of each SNP in squamous cell lung cancer on COVID-19 severity.

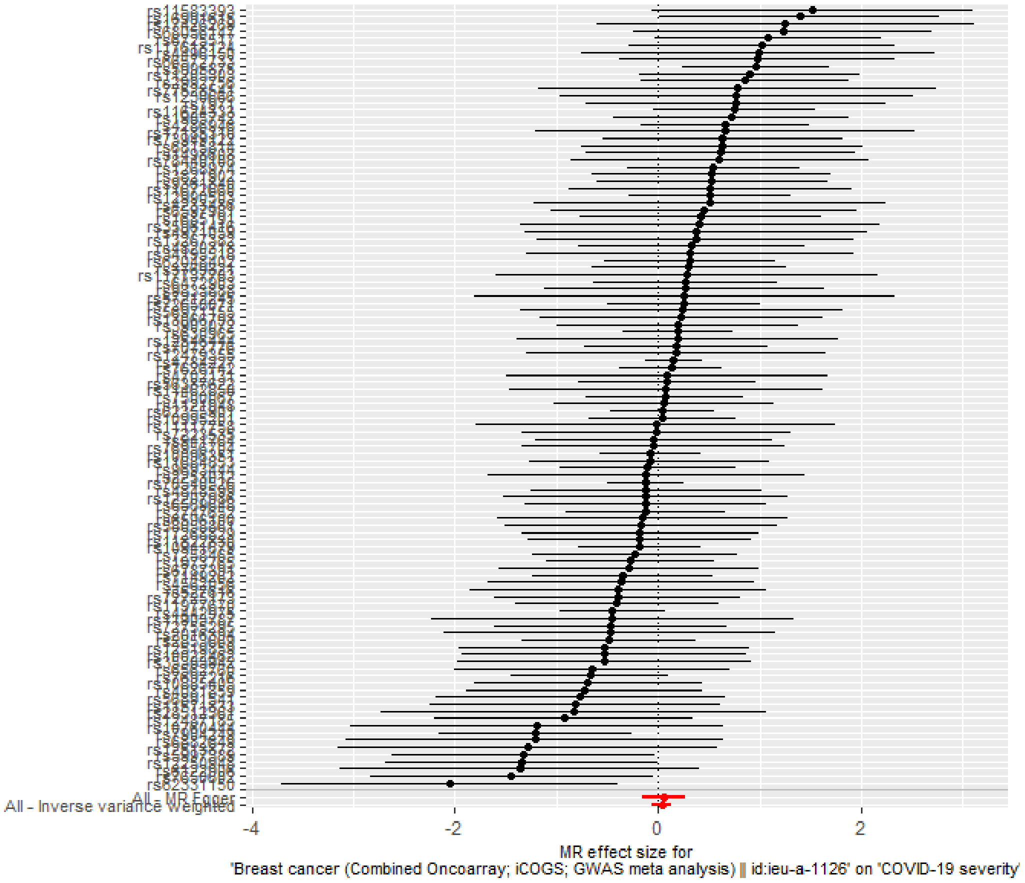

Figure S1D. The effect of each SNP in breast cancer on COVID-19 severity.

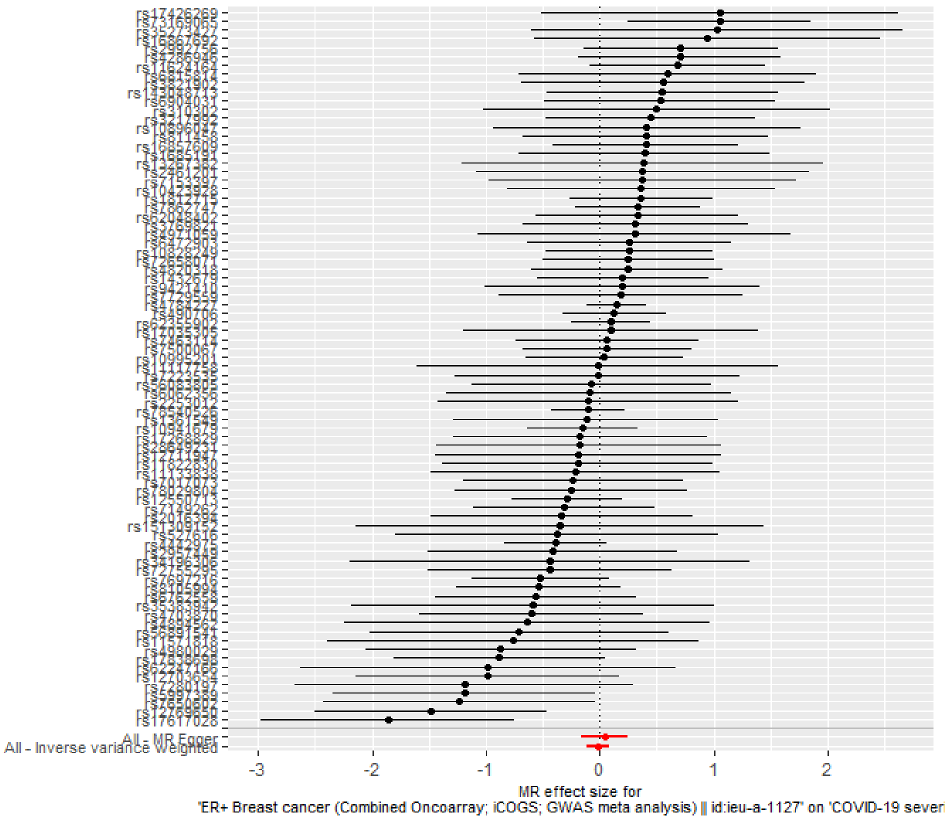

Figure S1E. The effect of each SNP in ER+ breast cancer on COVID-19 severity.

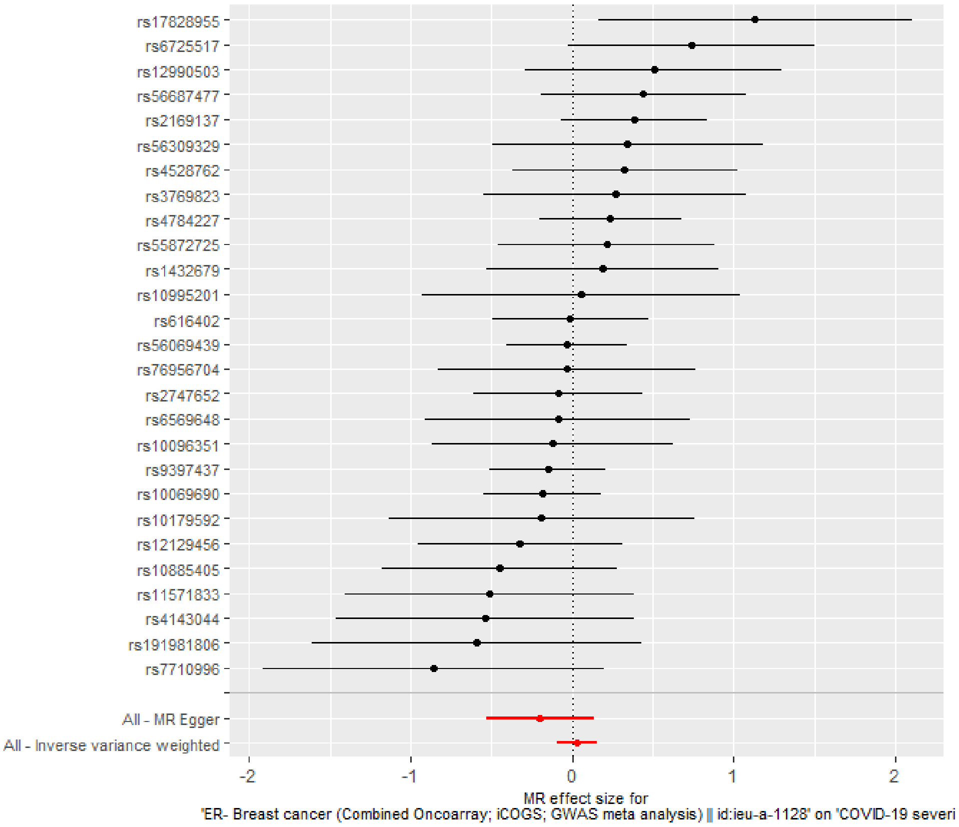

Figure S1F. The effect of each SNP in ER- breast cancer on COVID-19 severity.

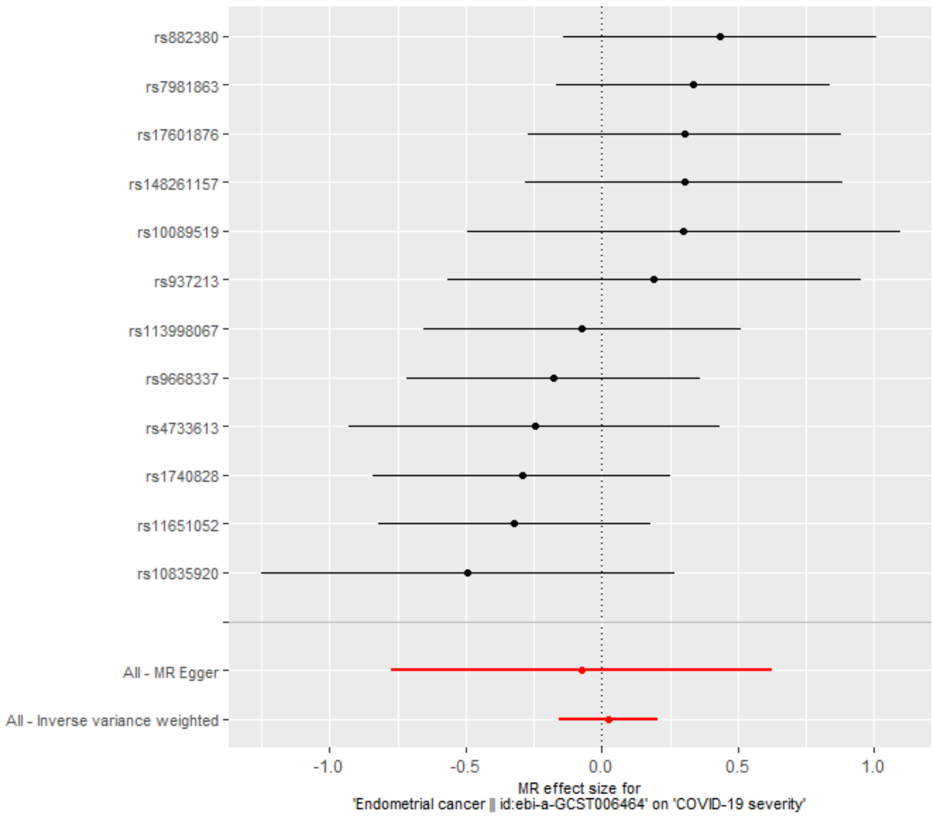

Figure S1G. The effect of each SNP in endometrial cancer on COVID-19 severity.

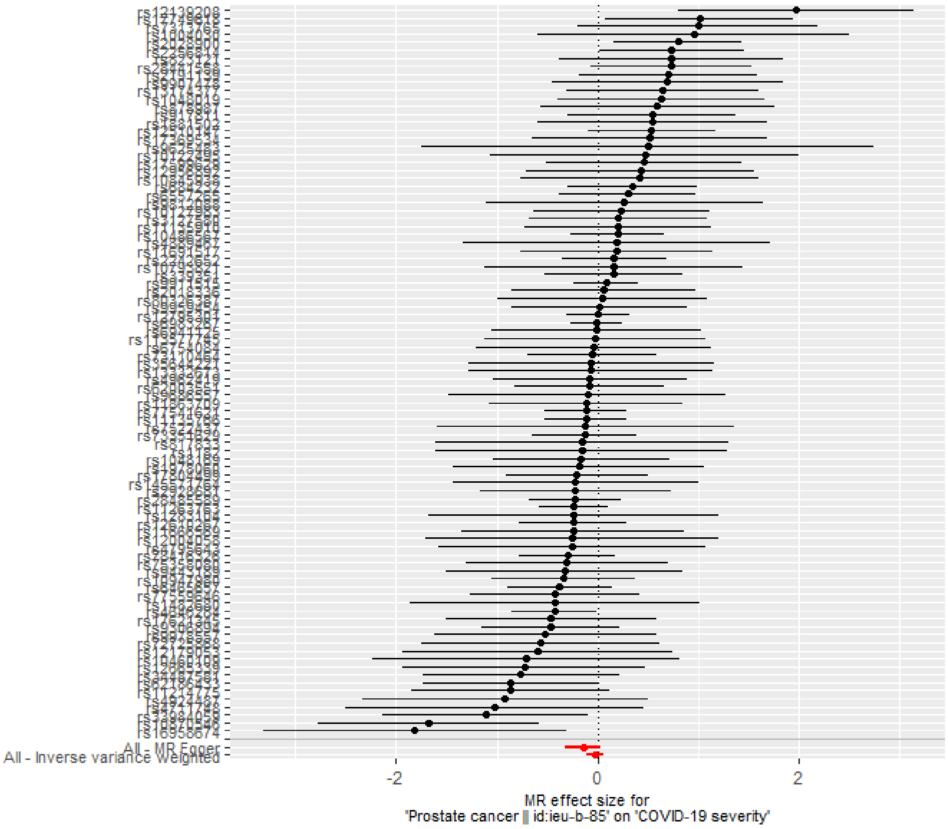

Figure S1H. The effect of each SNP in prostate cancer on COVID-19 severity.

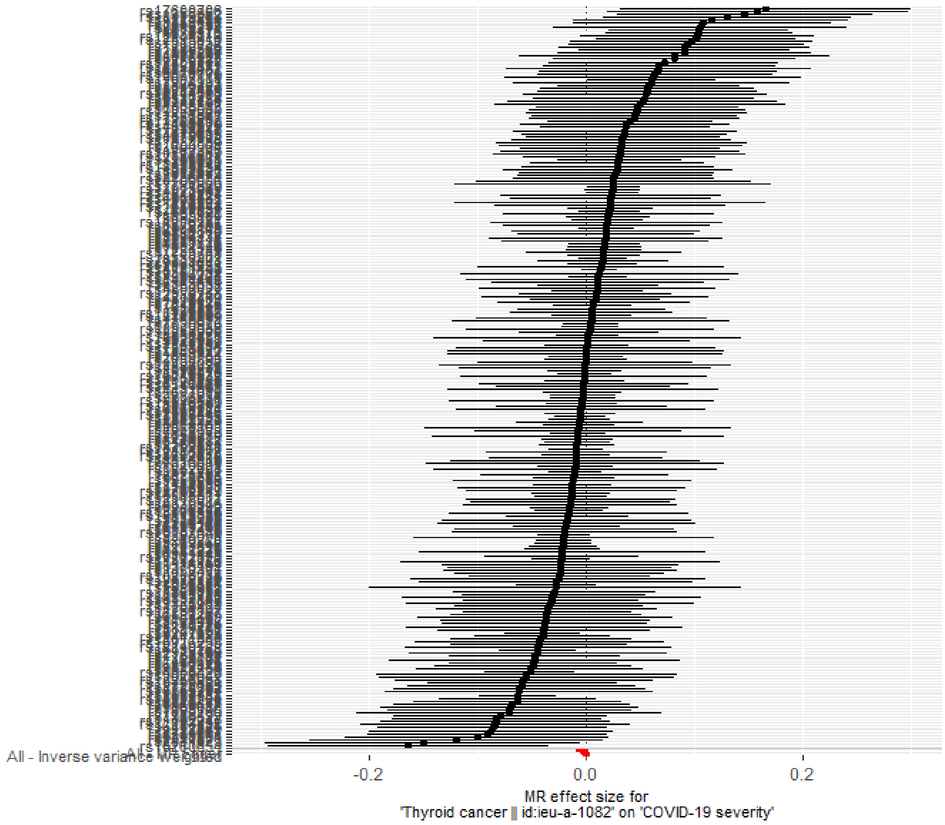

Figure S1I. The effect of each SNP in thyroid cancer on COVID-19 severity.

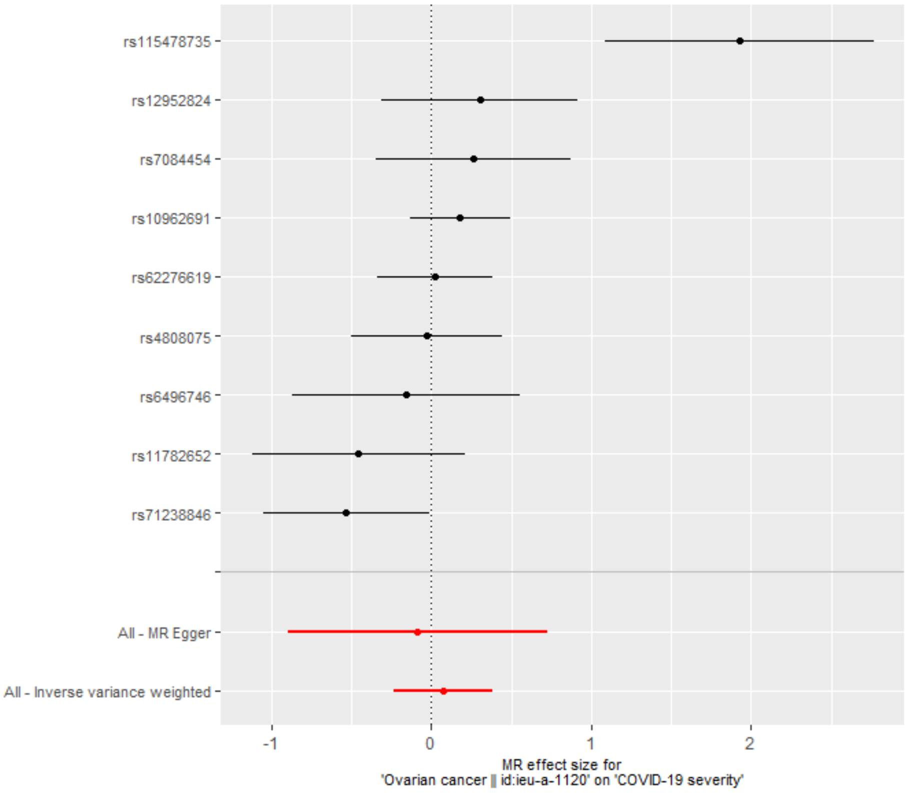

Figure S1J. The effect of each SNP in ovarian cancer on COVID-19 severity.

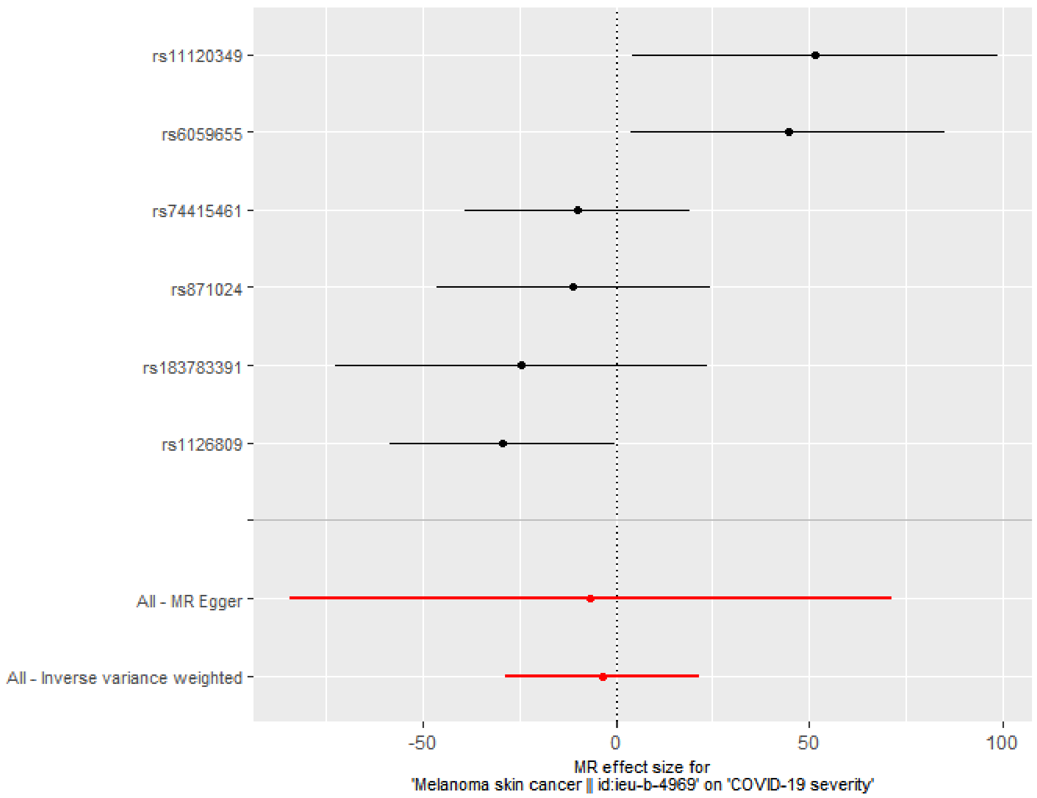

Figure S1K. The effect of each SNP in melanoma on COVID-19 severity.

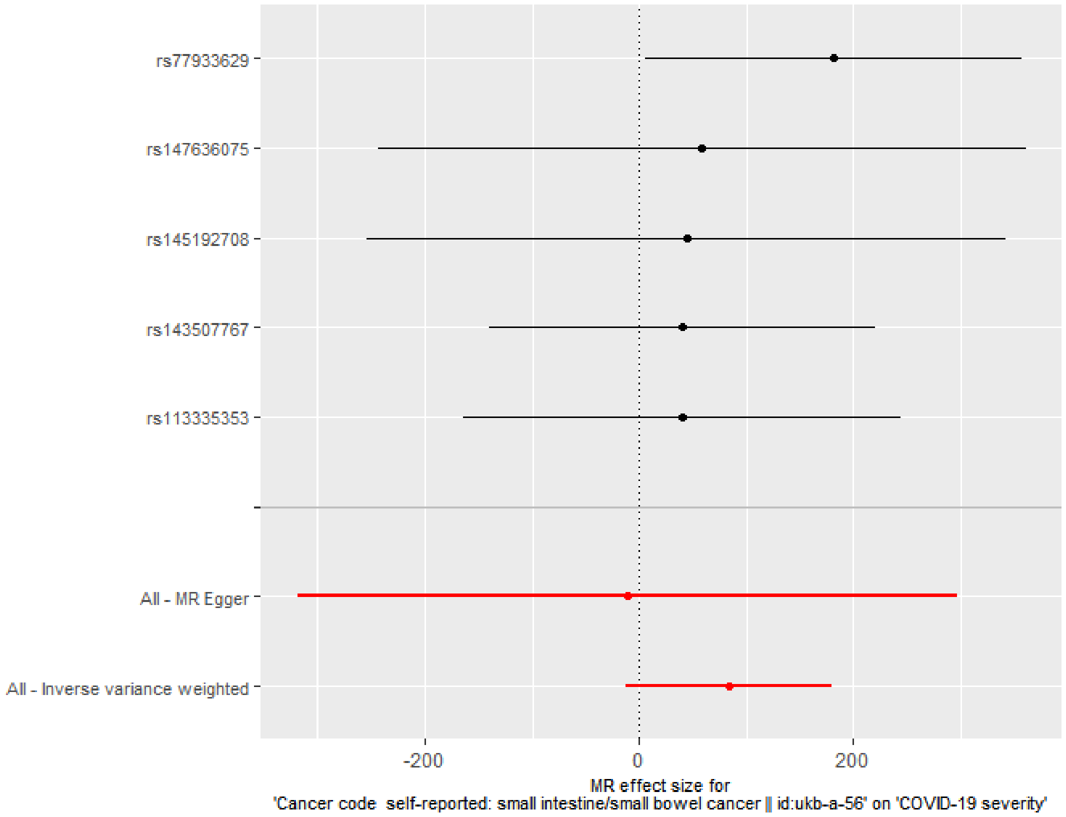

Figure S1L. The effect of each SNP in small bowel cancer on COVID-19 severity.

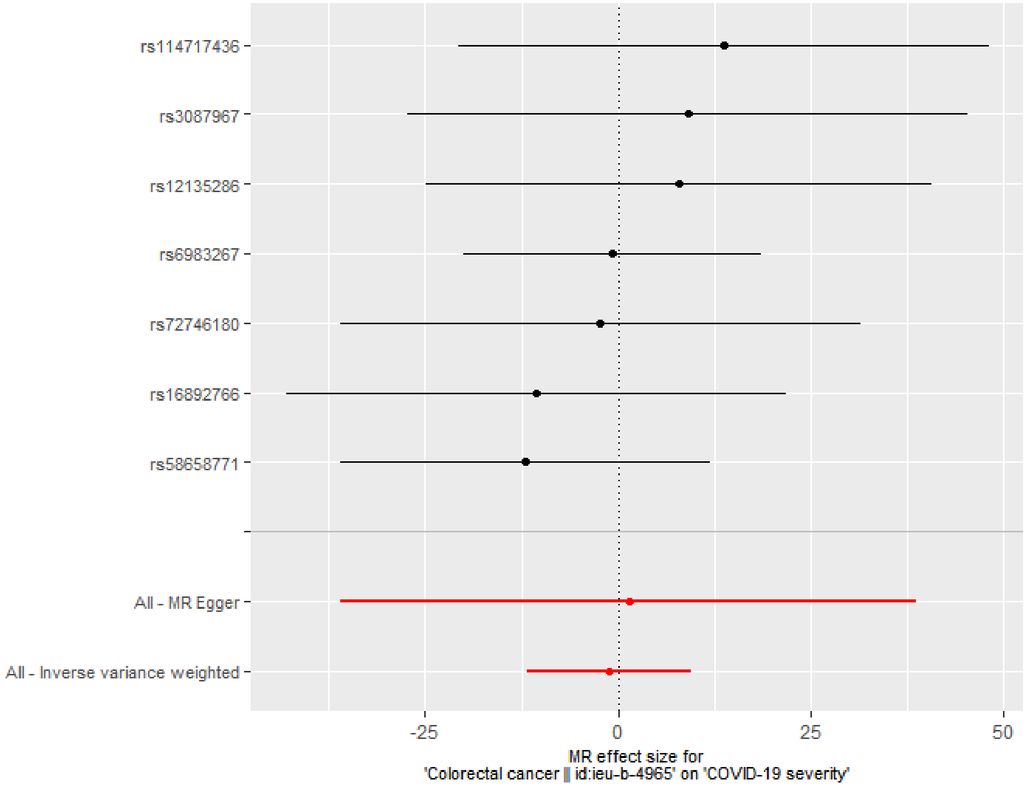

Figure S1M. The effect of each SNP in colorectal cancer on COVID-19 severity.

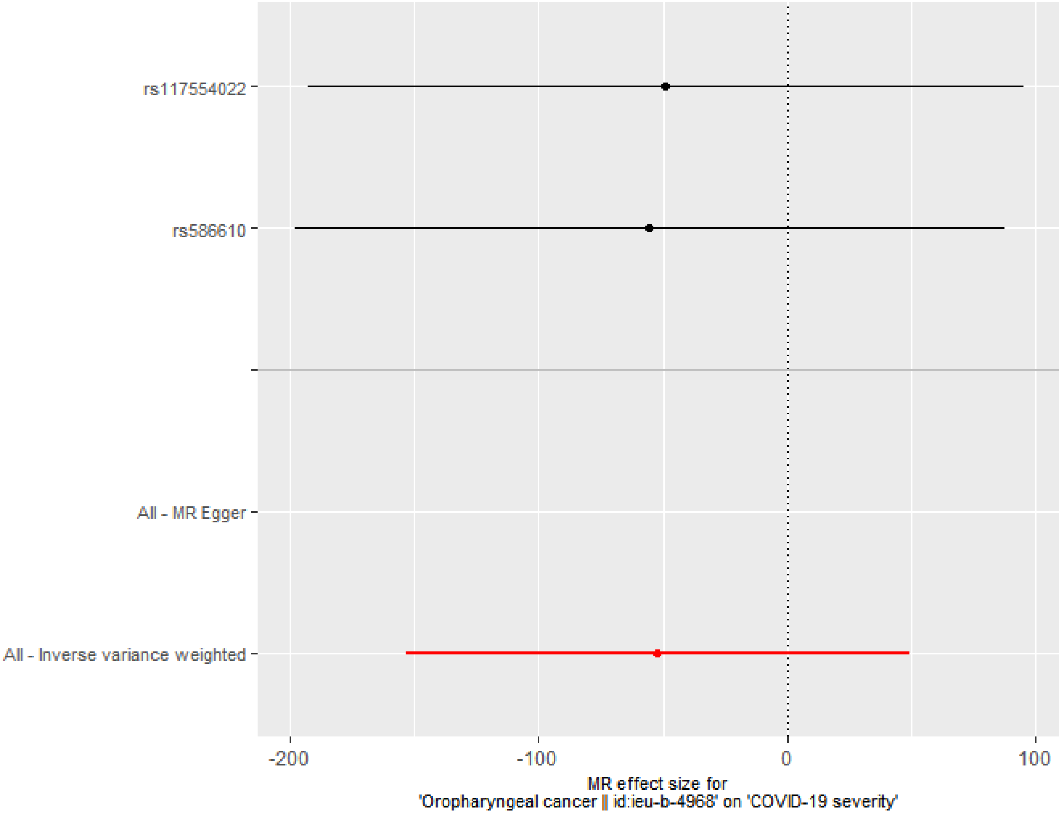

Figure S1N. The effect of each SNP in oropharyngeal cancer on COVID-19 severity.

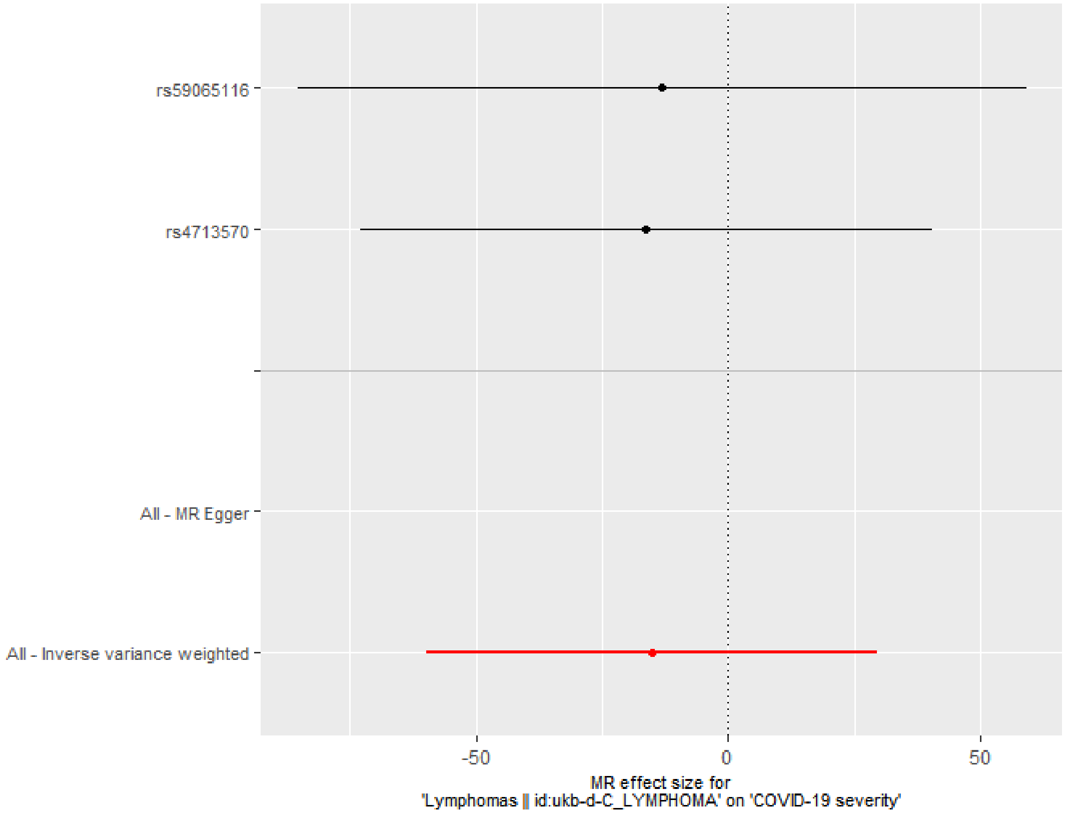

Figure S1O. The effect of each SNP in lymphoma on COVID-19 severity.

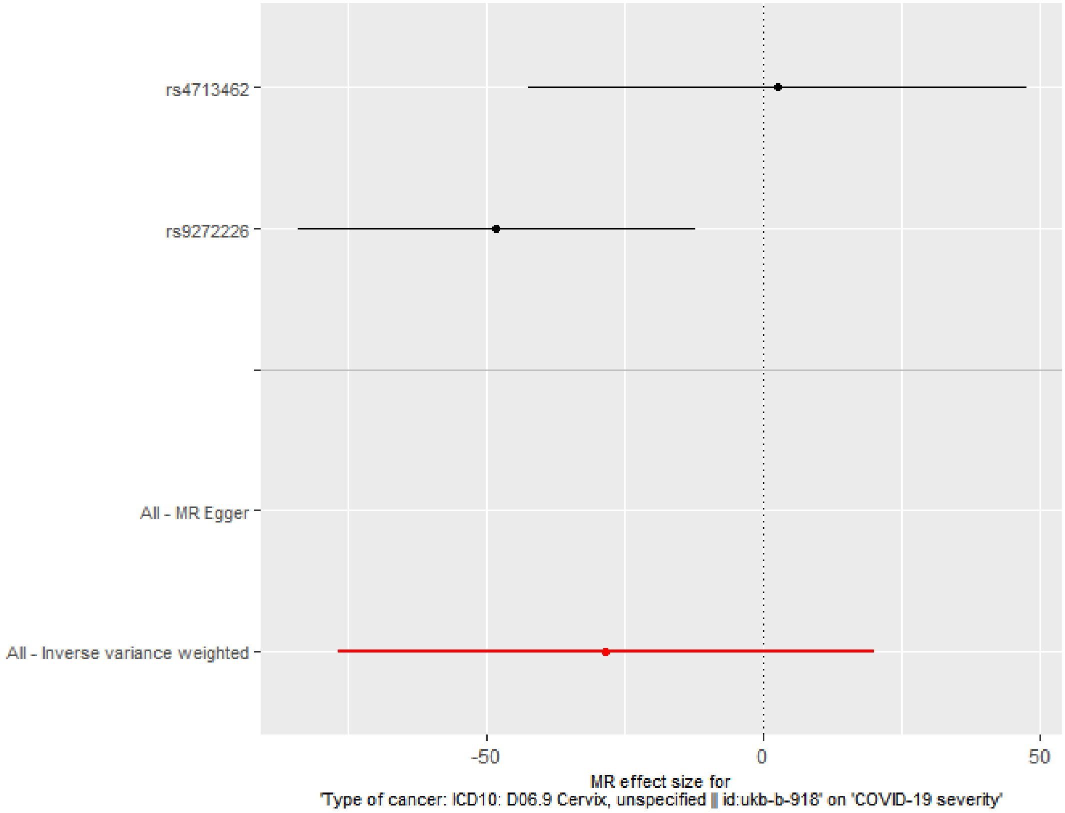

Figure S1P. The effect of each SNP in cervix cancer on COVID-19 severity.

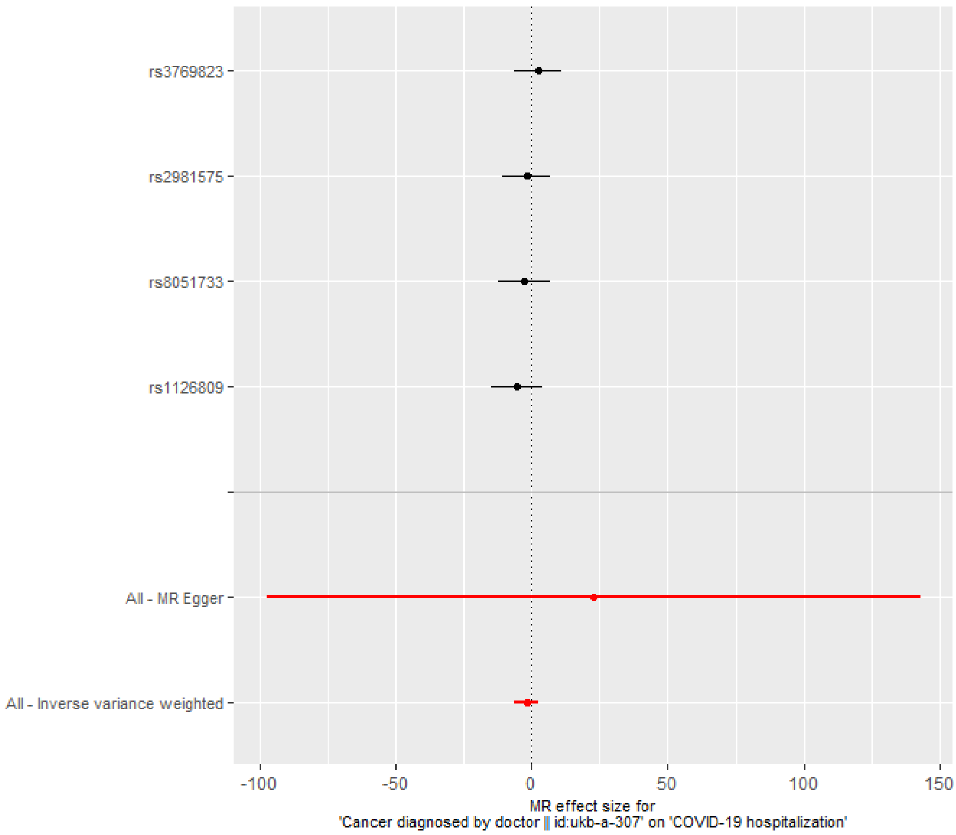

Figure S2A. The effect of each SNP in overall cancer on COVID-19 hospitalization.

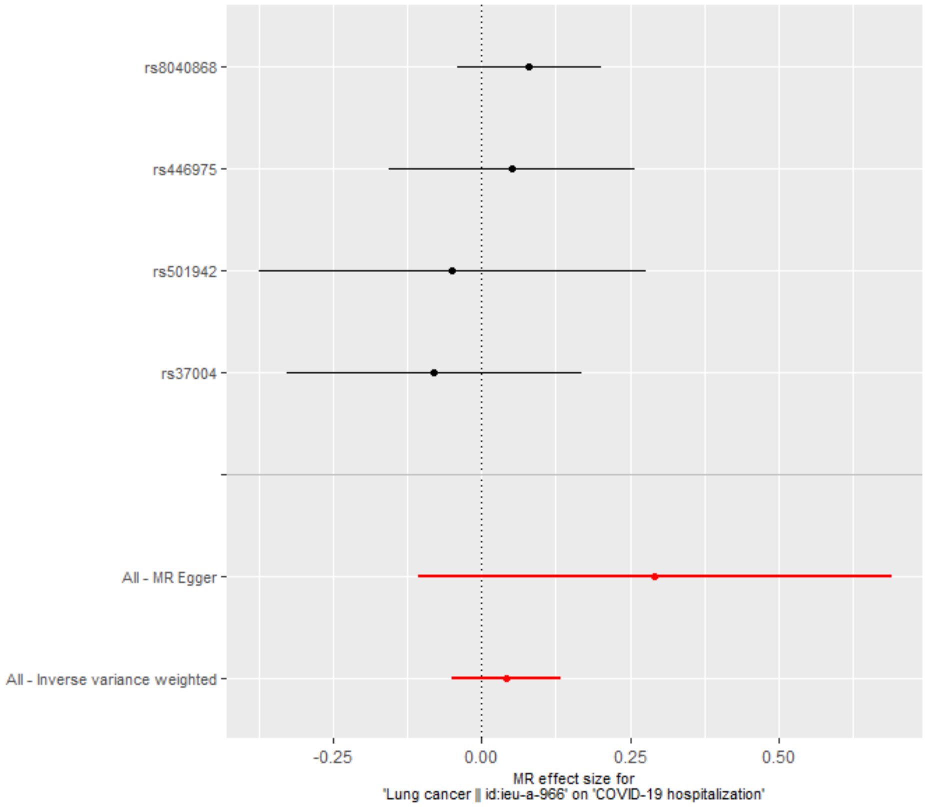

Figure S2B. The effect of each SNP in lung cancer on COVID-19 hospitalization.

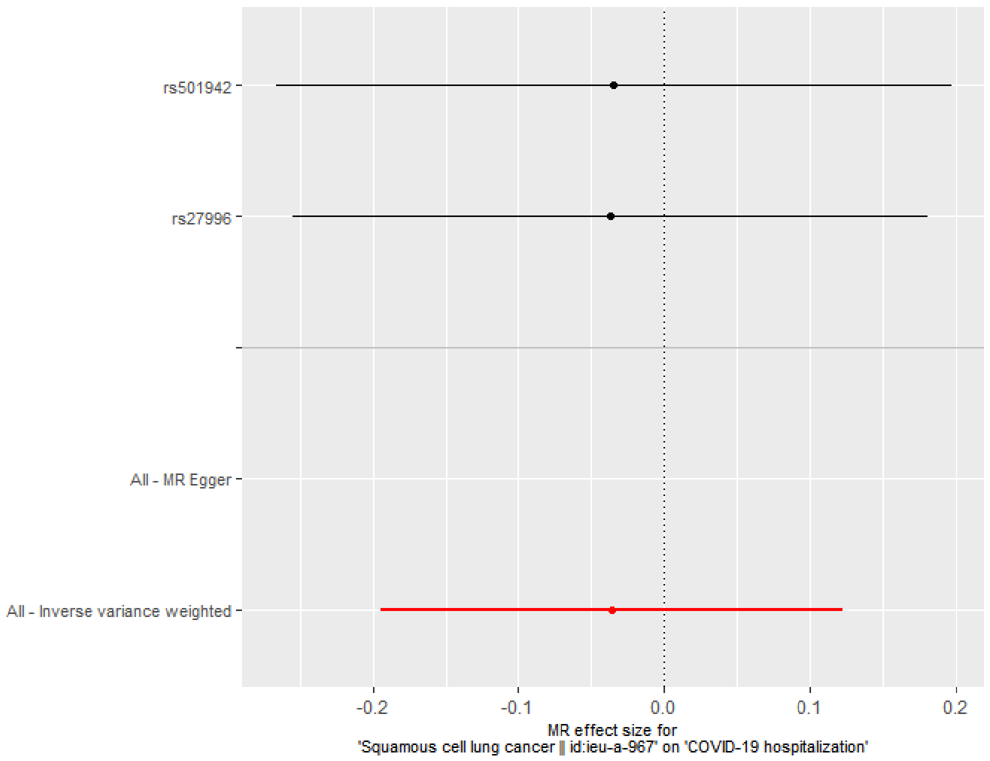

Figure S2C. The effect of each SNP in squamous cell lung cancer on COVID-19 hospitalization.

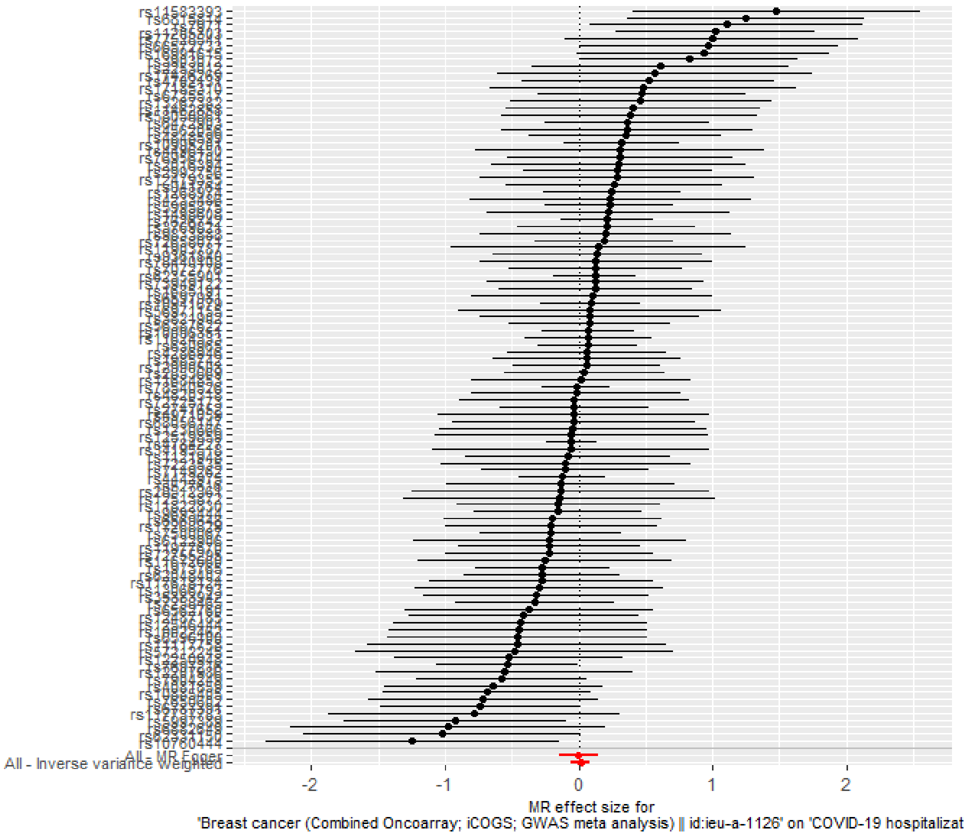

Figure S2D. The effect of each SNP in breast cancer on COVID-19 hospitalization.

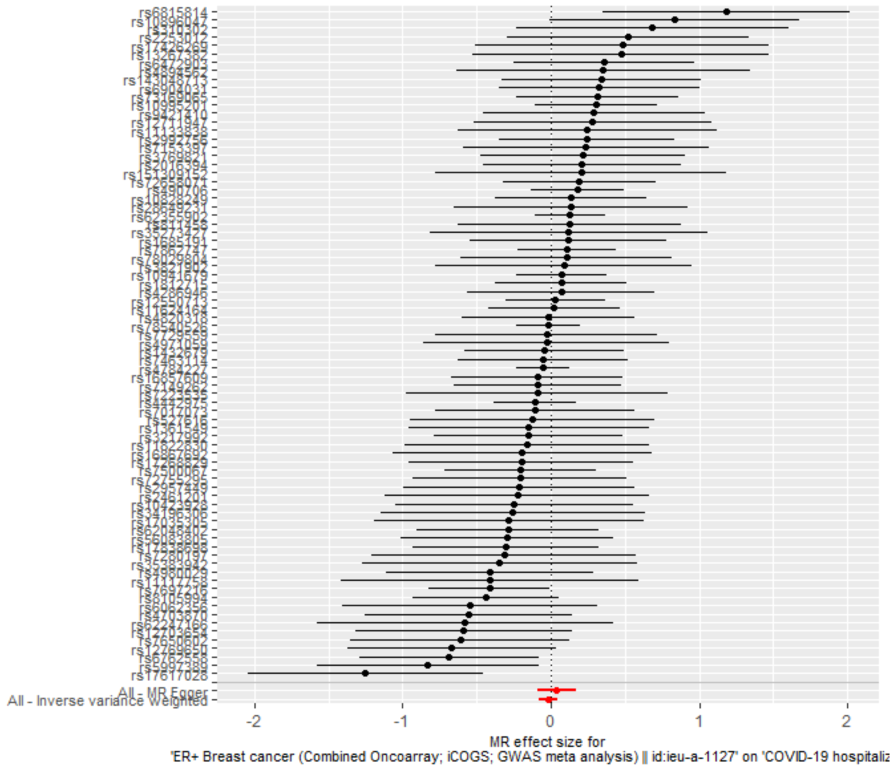

Figure S2E. The effect of each SNP in ER+ breast cancer on COVID-19 hospitalization.

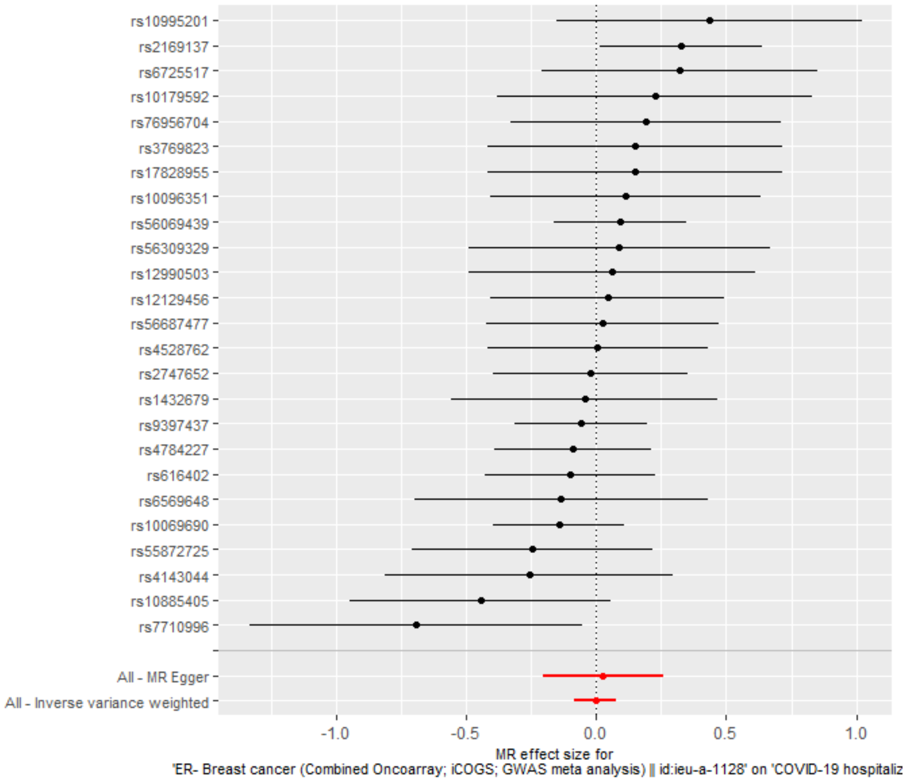

Figure S2F. The effect of each SNP in ER- breast cancer on COVID-19 hospitalization.

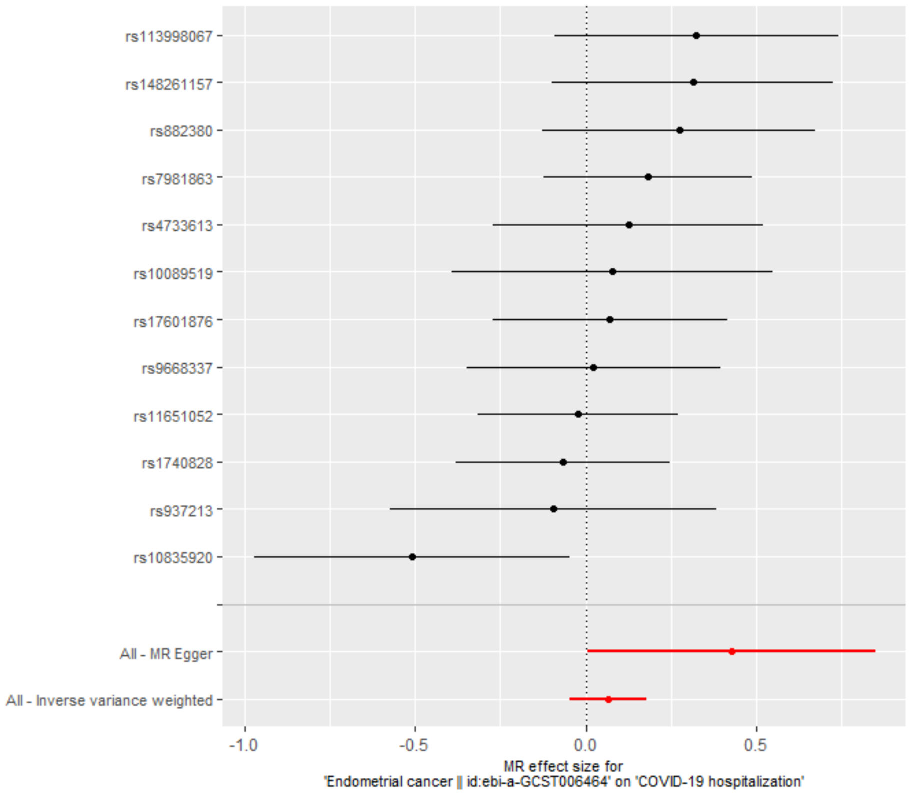

Figure S2G. The effect of each SNP in endometrial cancer on COVID-19 hospitalization.

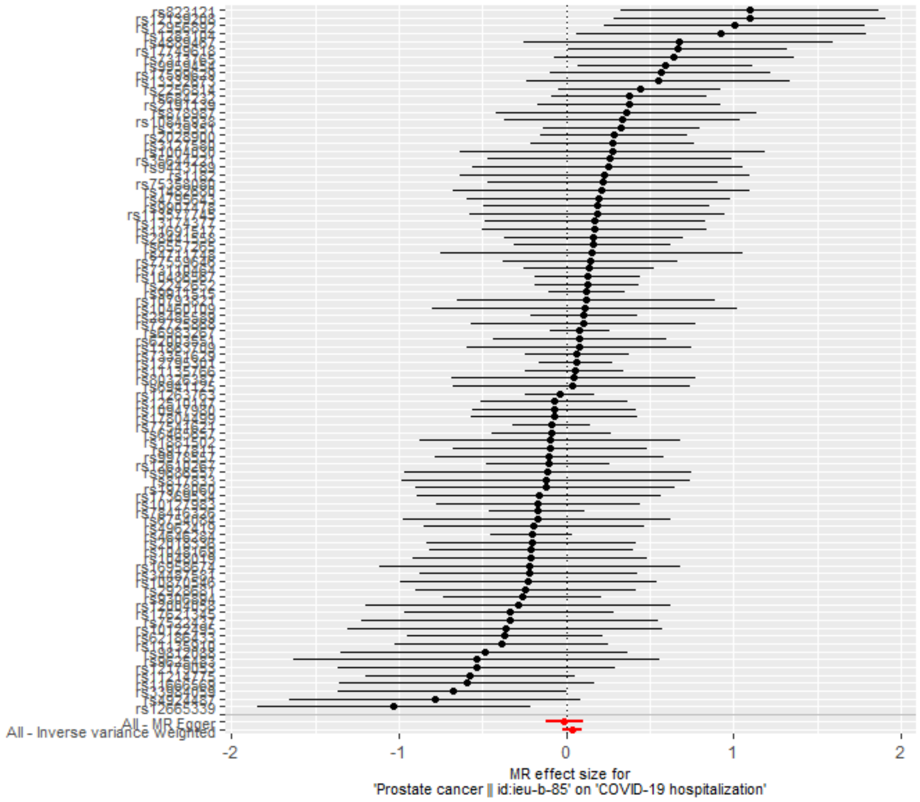

Figure S2H. The effect of each SNP in prostate cancer on COVID-19 hospitalization.

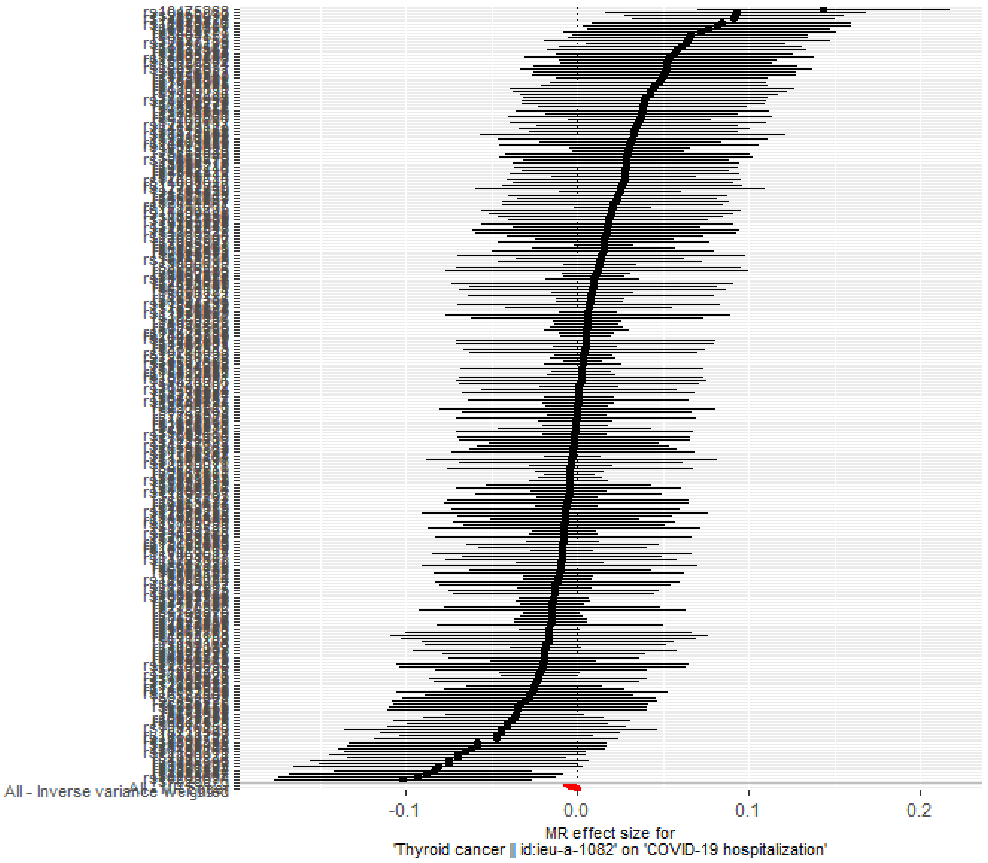

Figure S2I. The effect of each SNP in thyroid cancer on COVID-19 hospitalization.

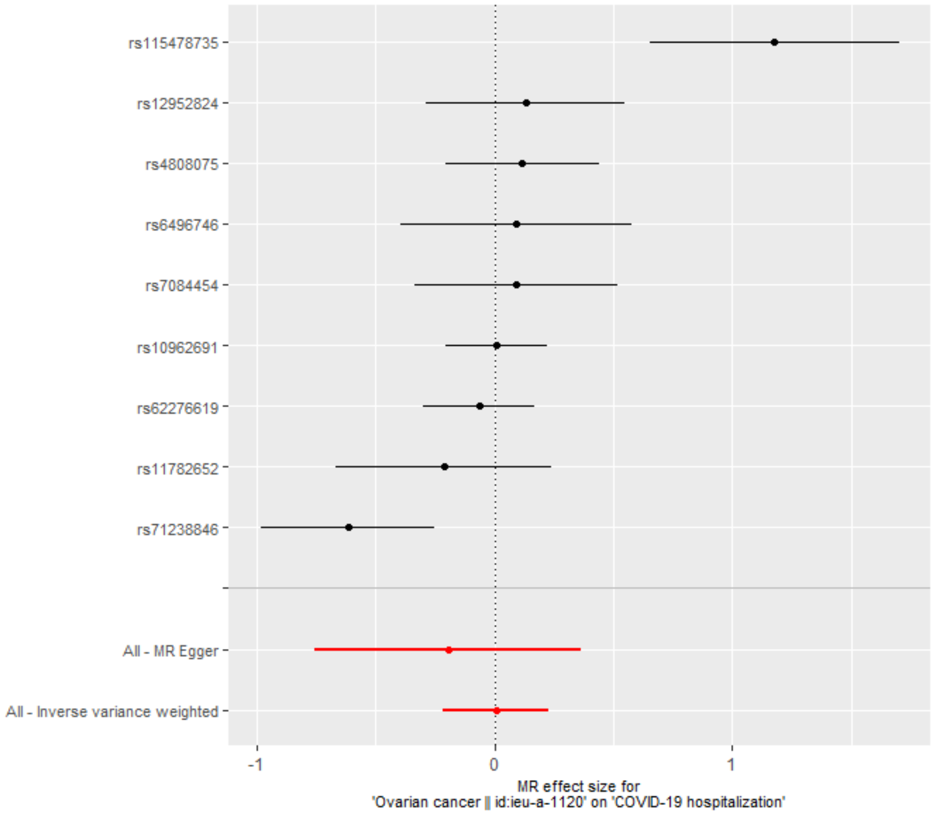

Figure S2J. The effect of each SNP in ovarian cancer on COVID-19 hospitalization.

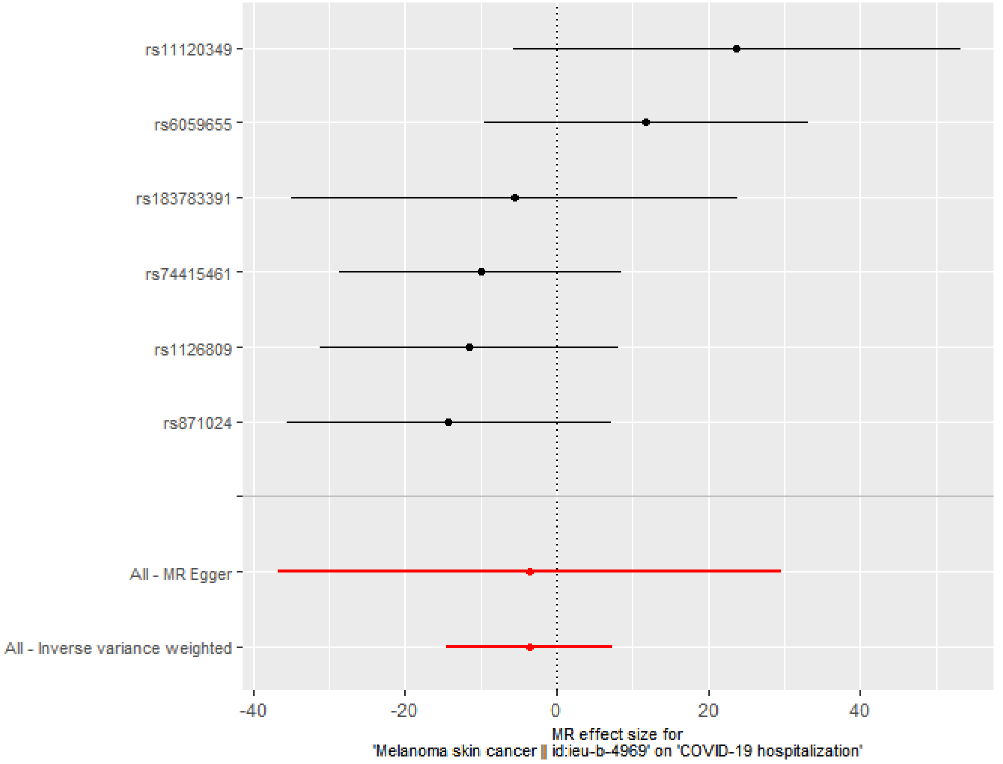

Figure S2K. The effect of each SNP in melanoma on COVID-19 hospitalization.

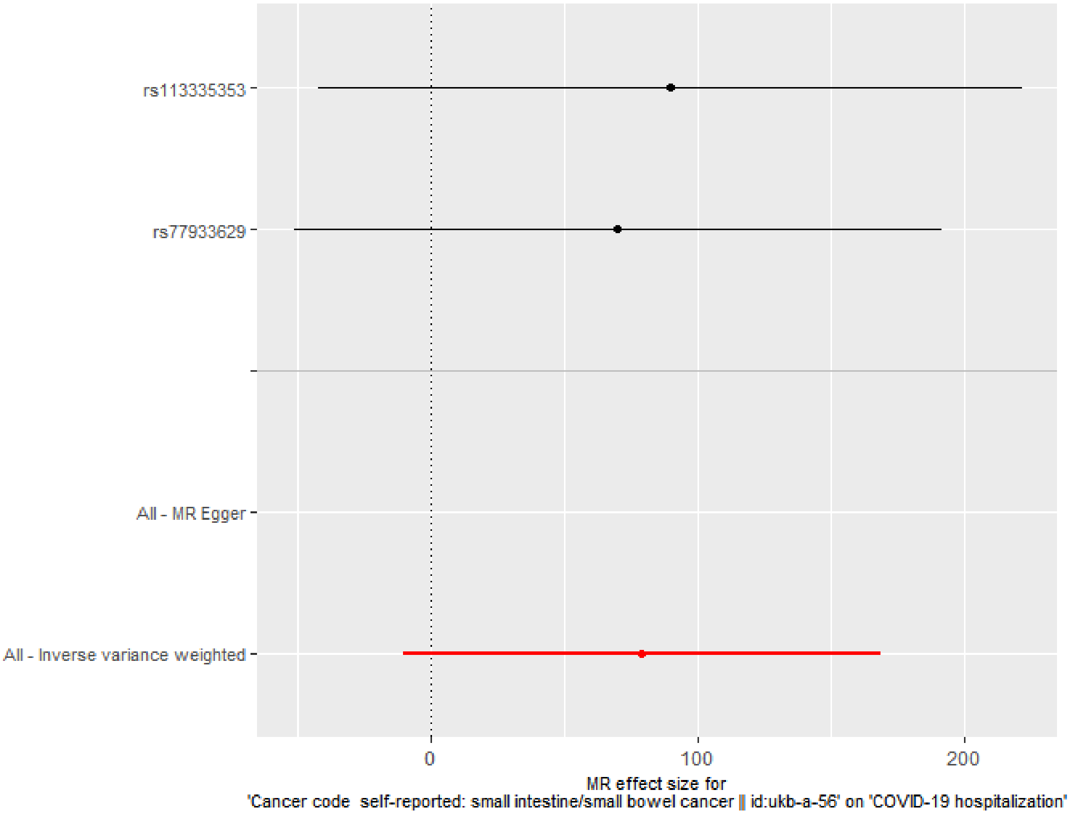

Figure S2L. The effect of each SNP in small bowel cancer on COVID-19 hospitalization.

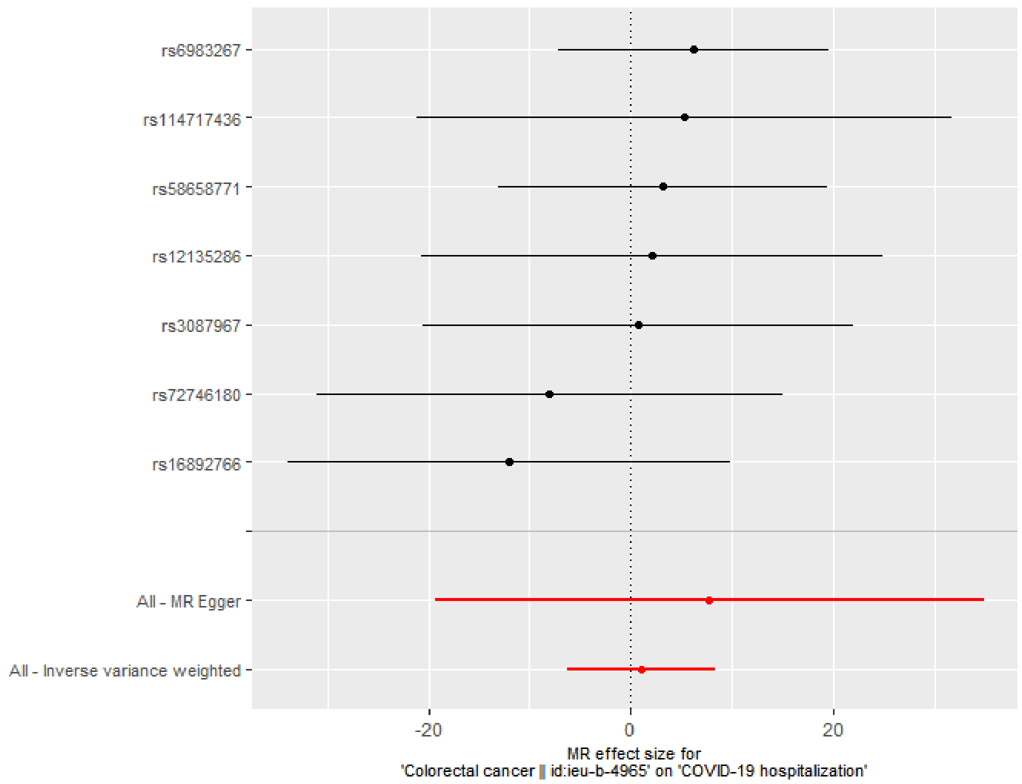

Figure S2M. The effect of each SNP in colorectal cancer on COVID-19 hospitalization.

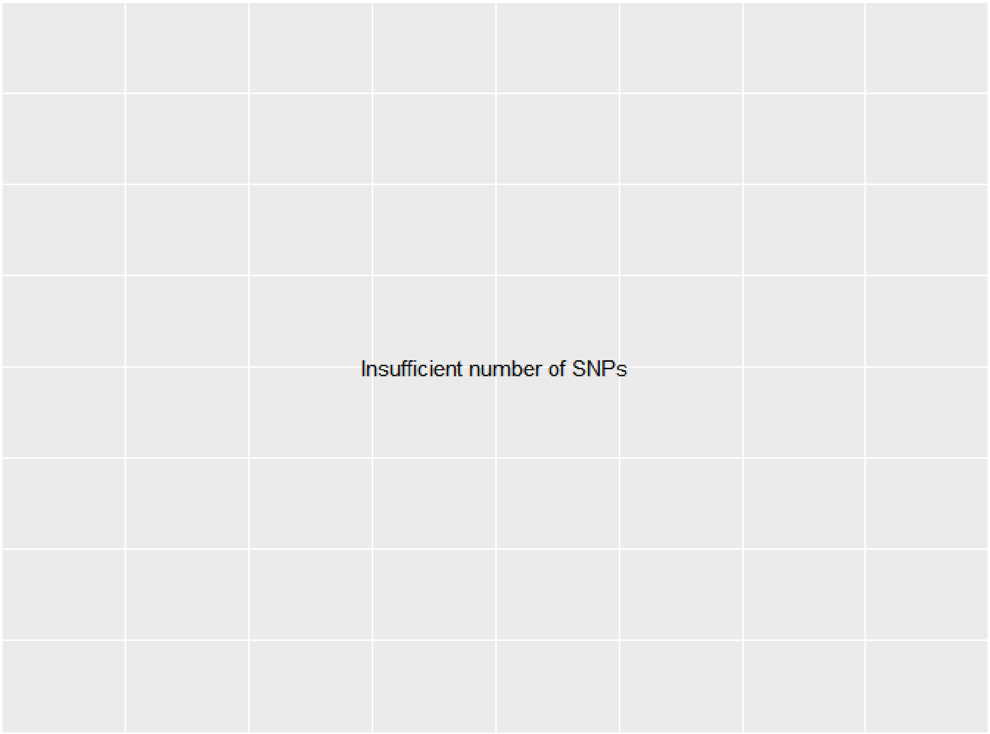

Figure S2N. The effect of each SNP in oropharyngeal cancer on COVID-19 hospitalization.

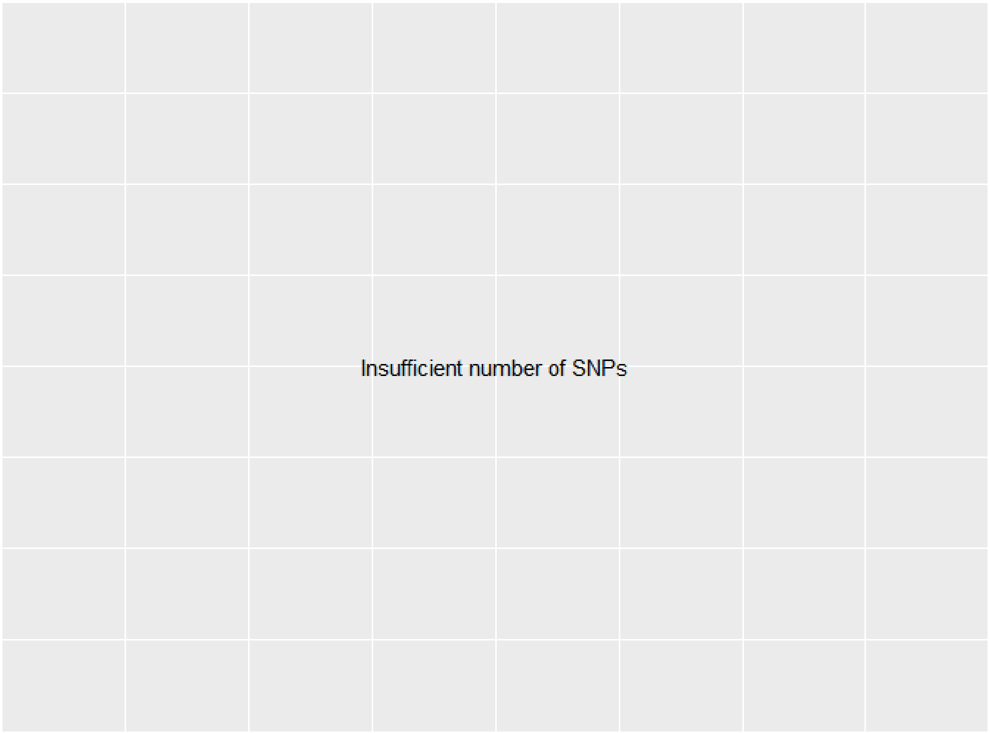

Figure S2O. The effect of each SNP in lymphoma on COVID-19 hospitalization.

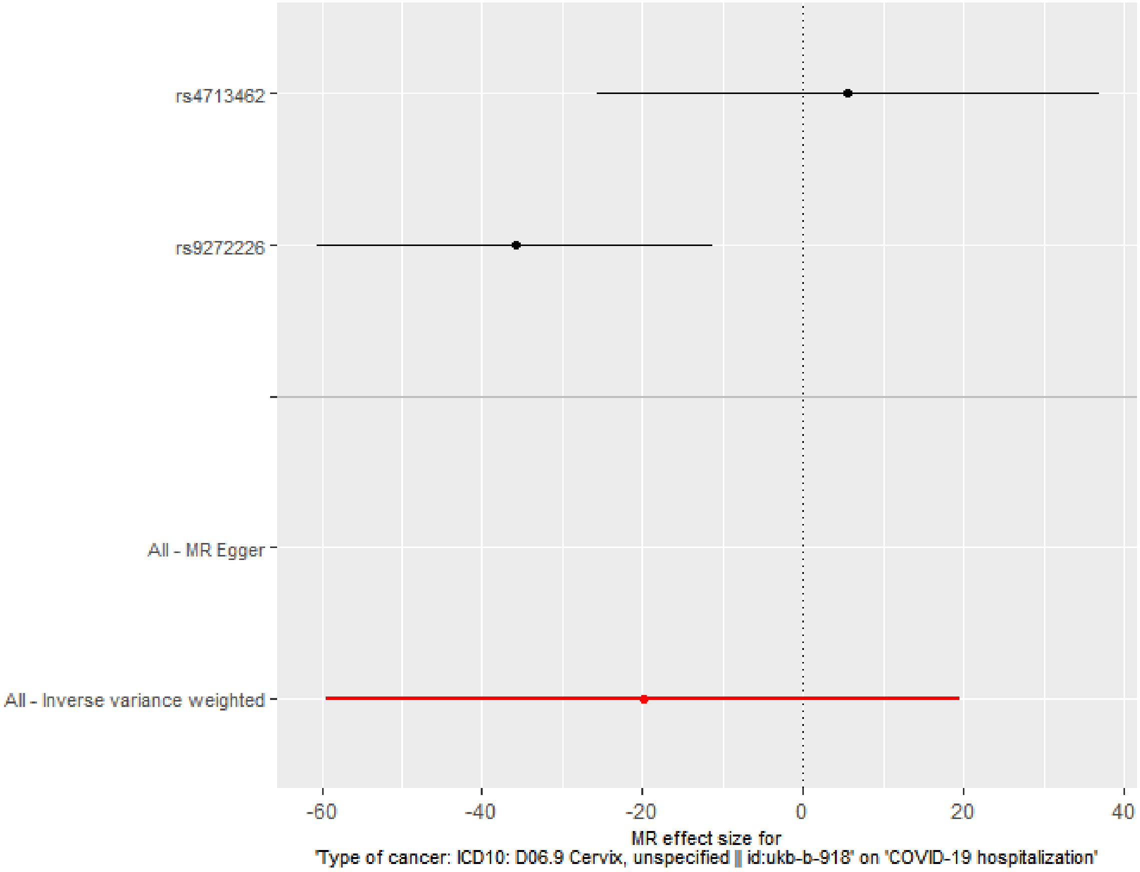

Figure S2P. The effect of each SNP in cervix cancer on COVID-19 hospitalization.

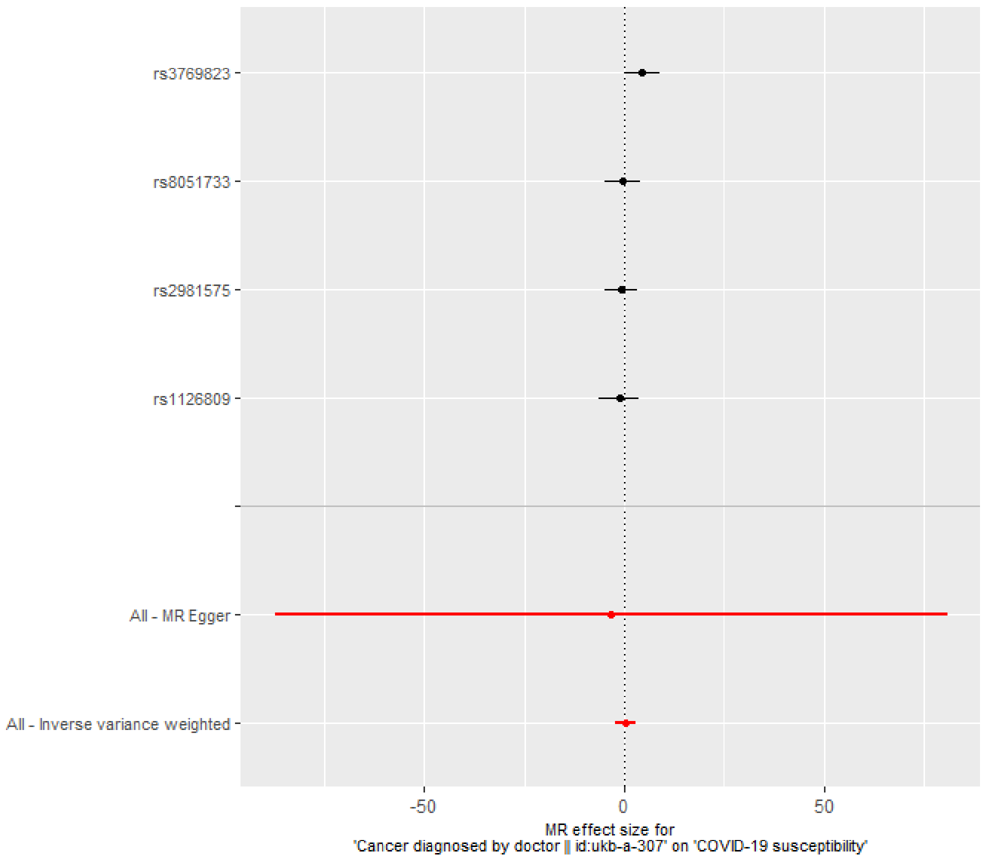

Figure S3A. The effect of each SNP in overall cancer on COVID-19 susceptibility.

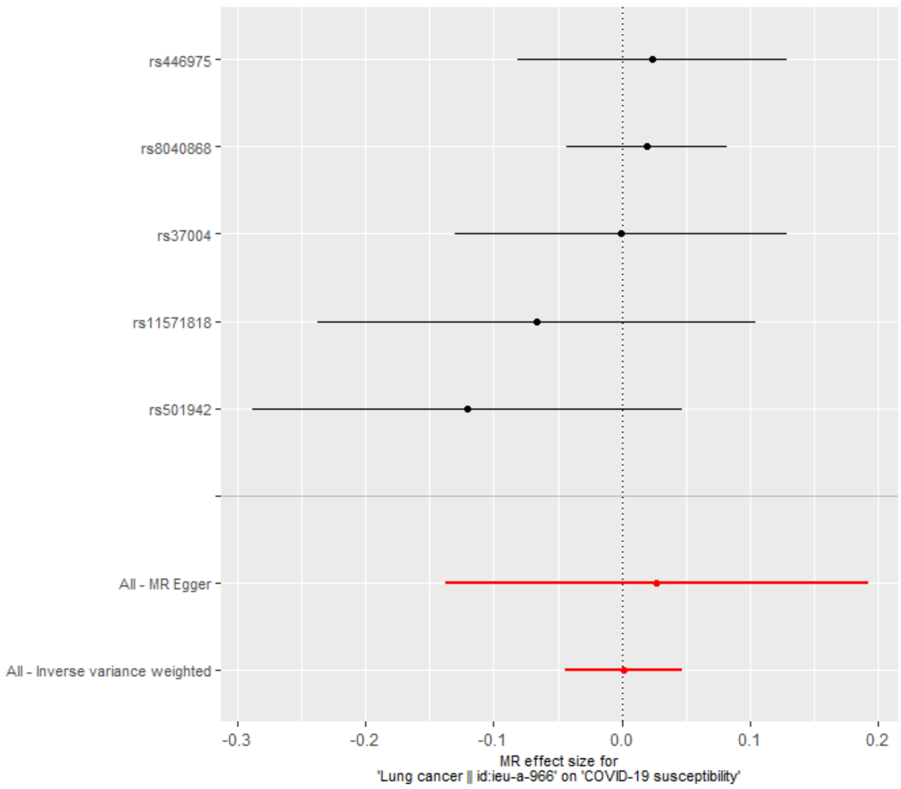

Figure S3B. The effect of each SNP in lung cancer on COVID-19 susceptibility.

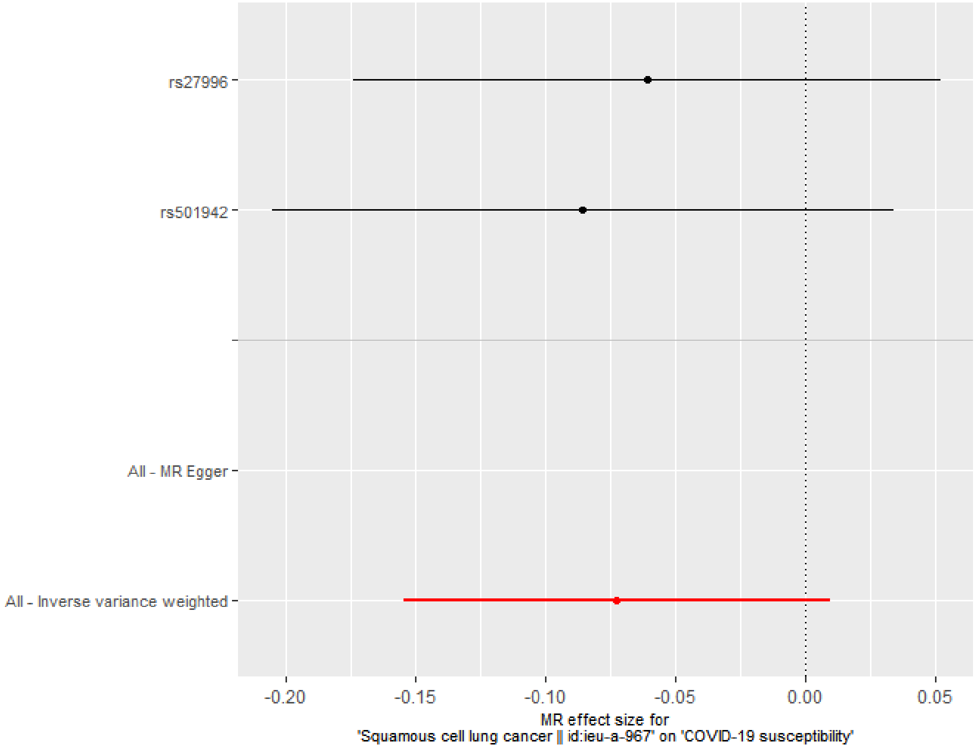

Figure S3C. The effect of each SNP in squamous cell lung cancer on COVID-19 susceptibility.

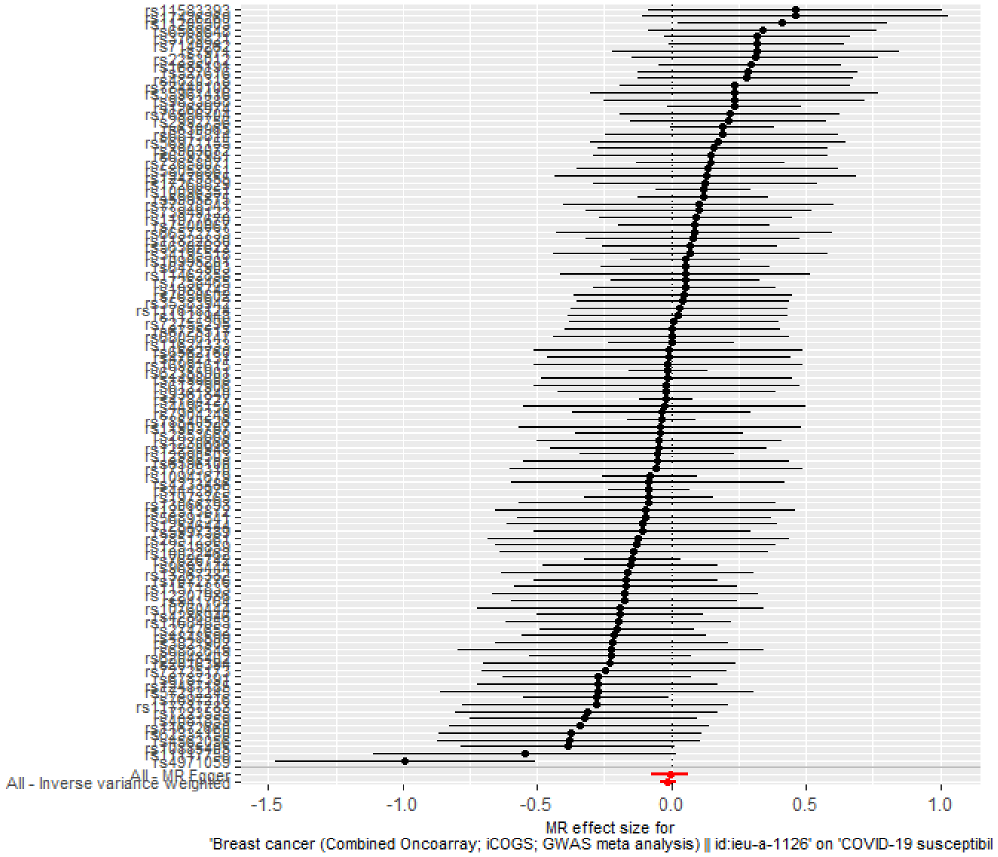

Figure S3D. The effect of each SNP in breast cancer on COVID-19 susceptibility.

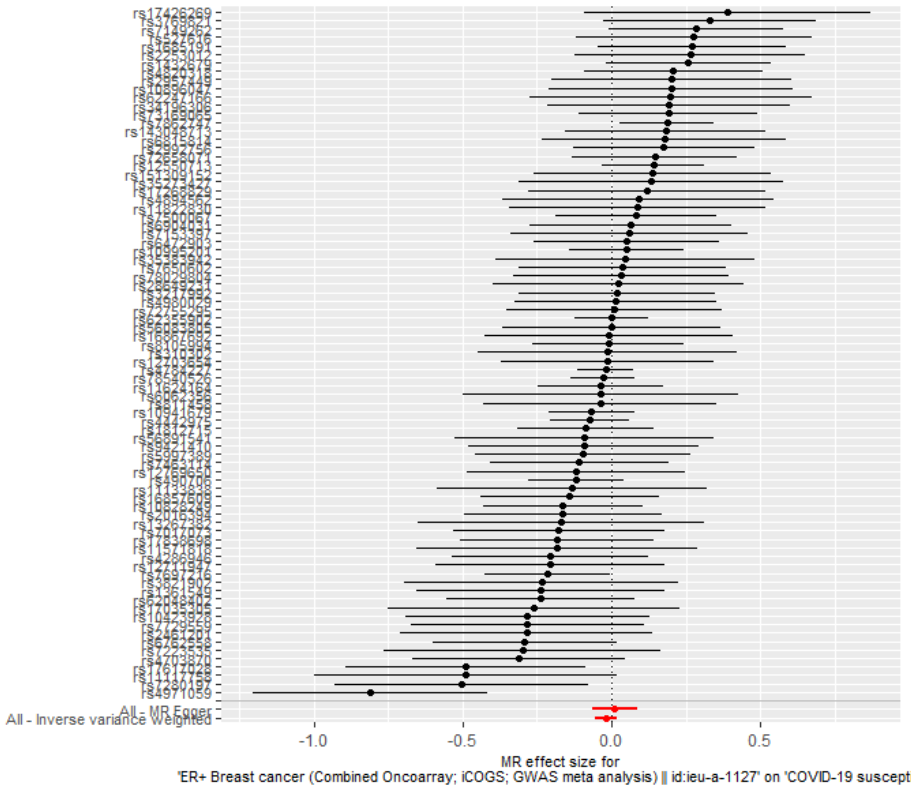

Figure S3E. The effect of each SNP in ER+ breast cancer on COVID-19 susceptibility.

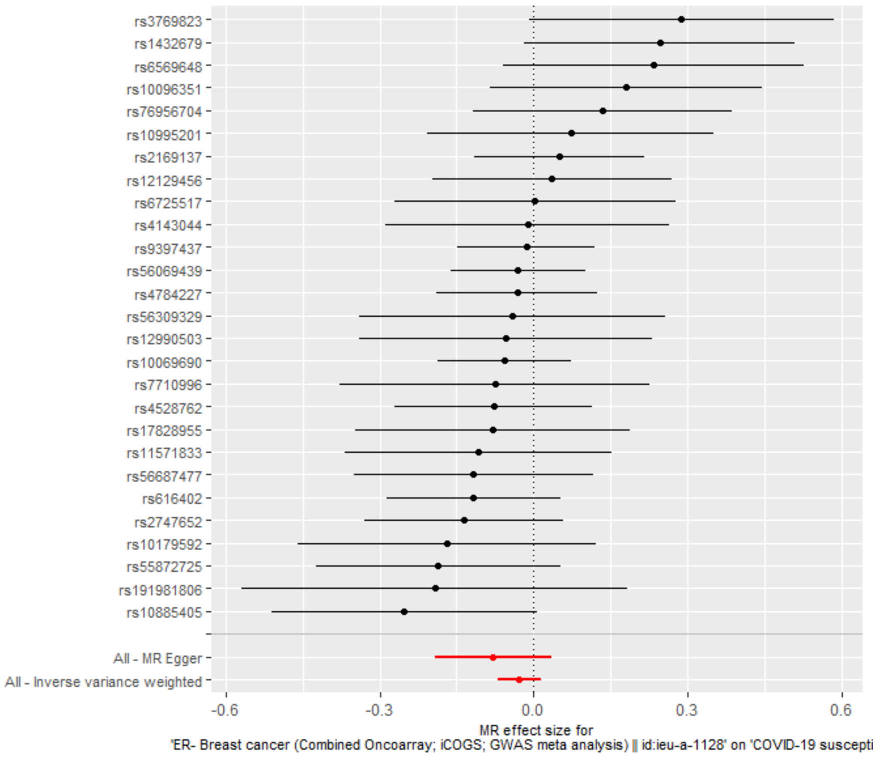

Figure S3F. The effect of each SNP in ER- breast cancer on COVID-19 susceptibility.

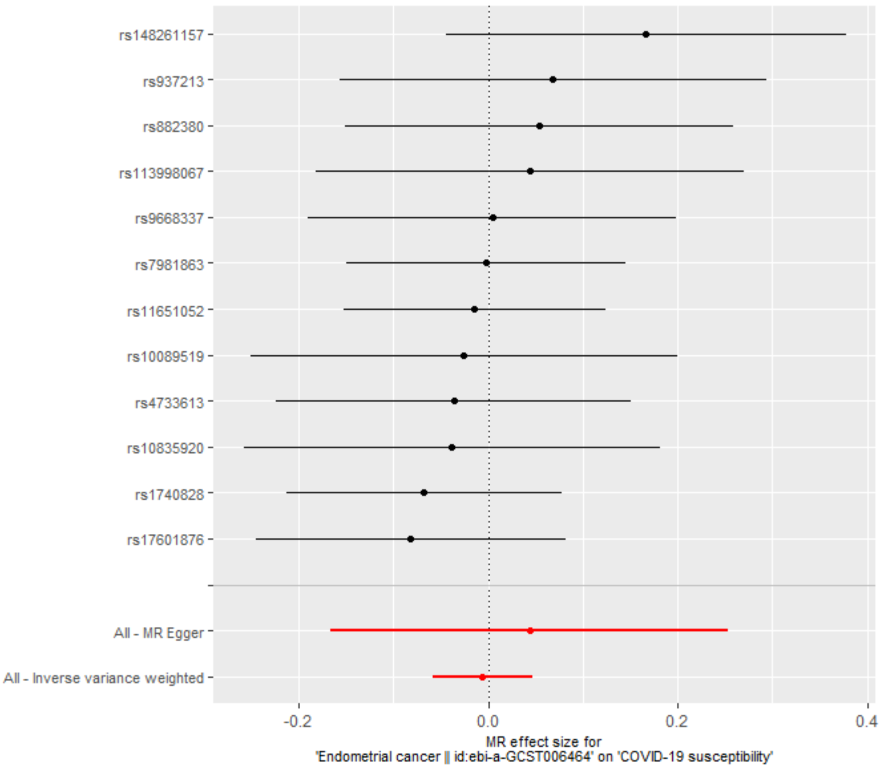

Figure S3G. The effect of each SNP in endometrial cancer on COVID-19 susceptibility.

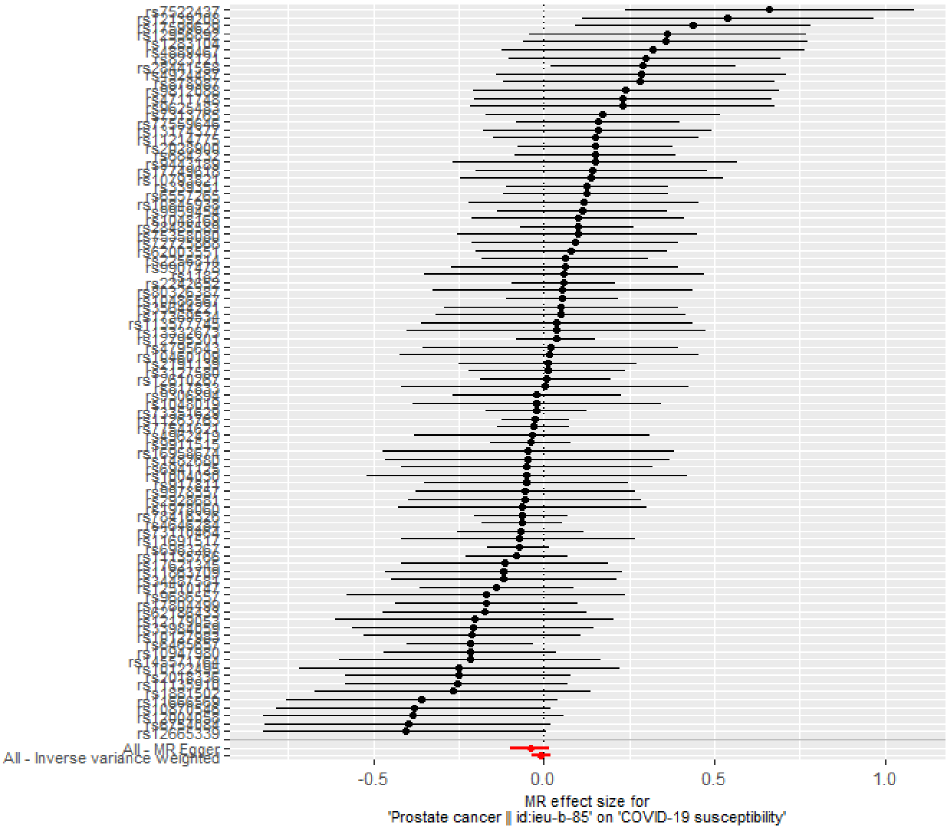

Figure S3H. The effect of each SNP in prostate cancer on COVID-19 susceptibility.

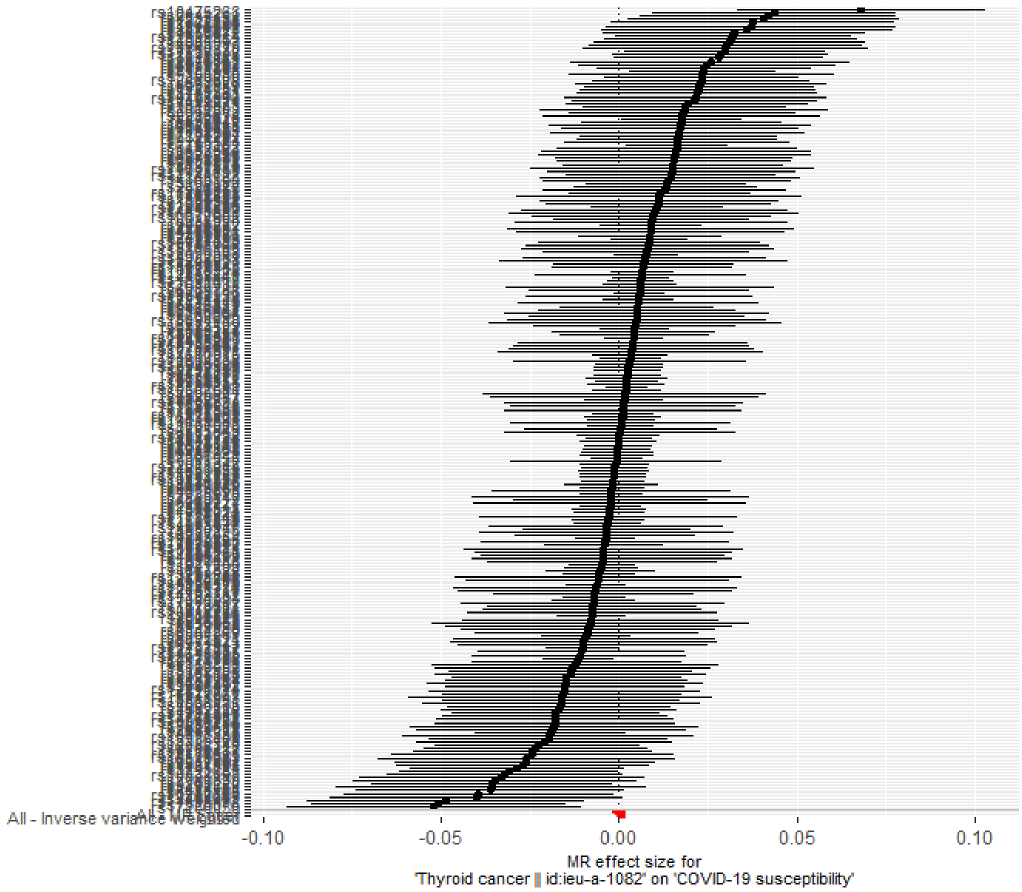

Figure S3I. The effect of each SNP in thyroid cancer on COVID-19 susceptibility.

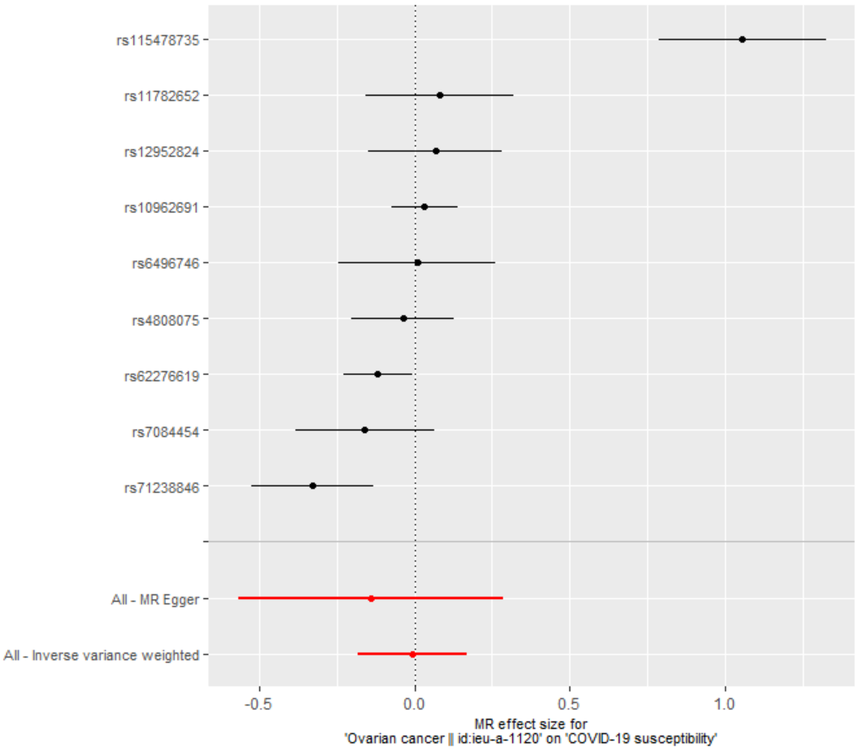

Figure S3J. The effect of each SNP in ovarian cancer on COVID-19 susceptibility.

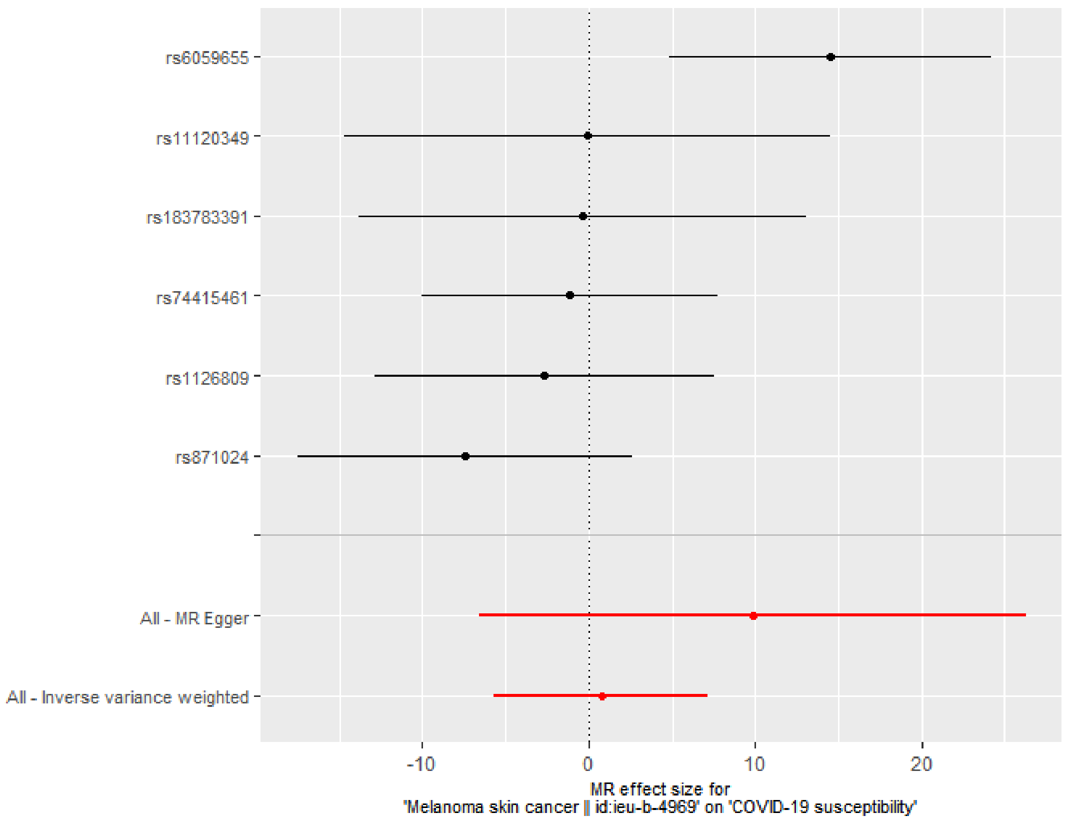

Figure S3K. The effect of each SNP in melanoma on COVID-19 susceptibility.

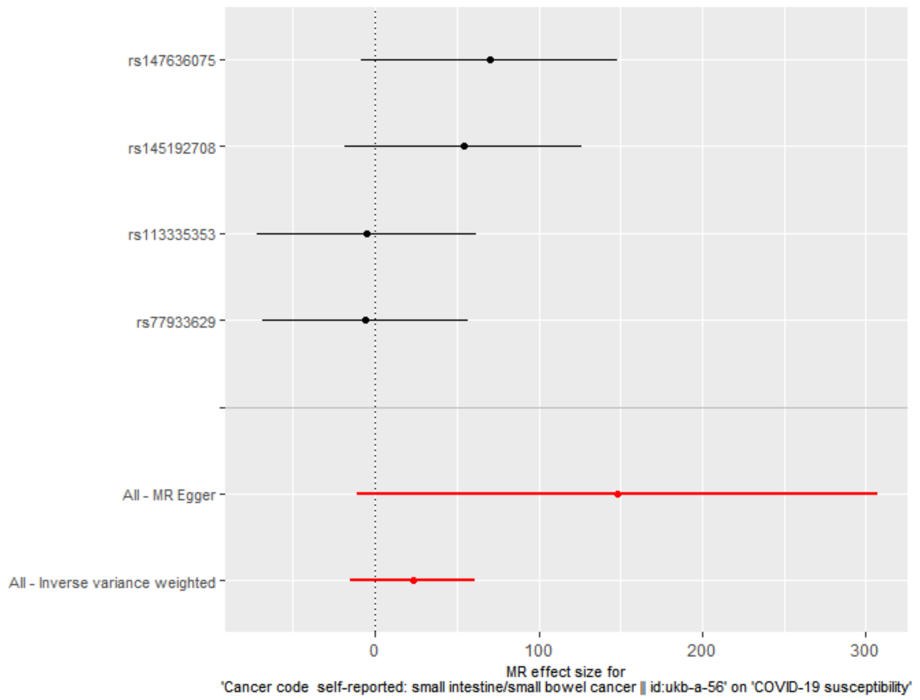

Figure S3L. The effect of each SNP in small bowel cancer on COVID-19 susceptibility.

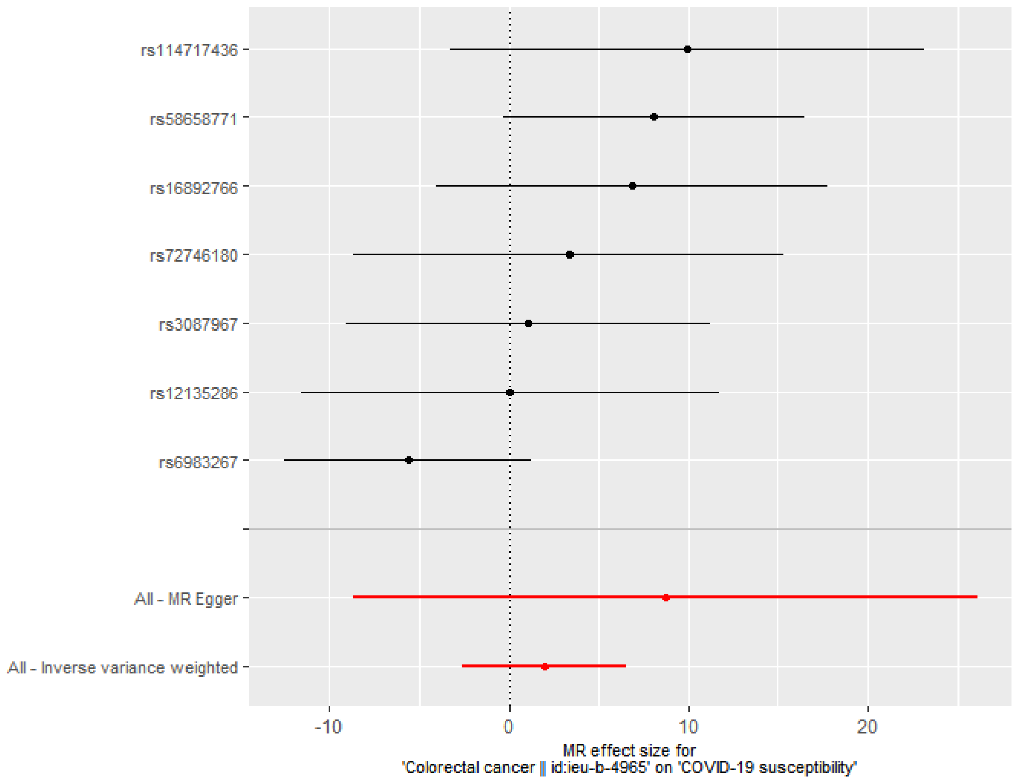

Figure S3M. The effect of each SNP in colorectal cancer on COVID-19 susceptibility.

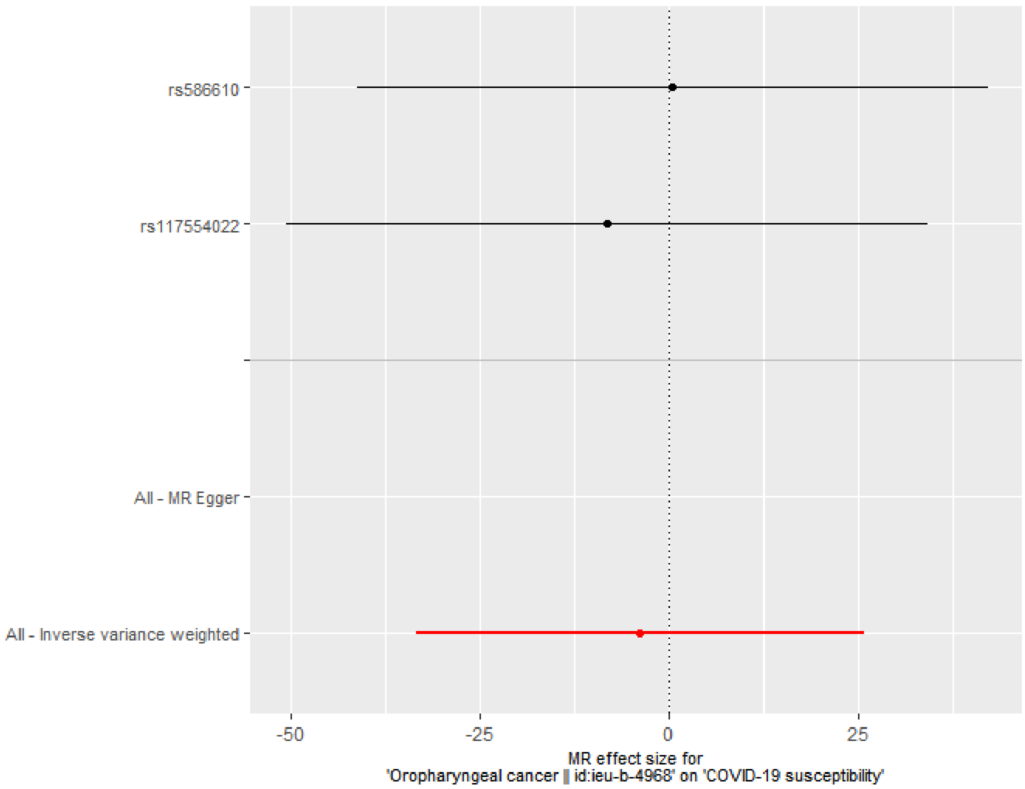

Figure S3N. The effect of each SNP in oropharyngeal cancer on COVID-19 susceptibility.

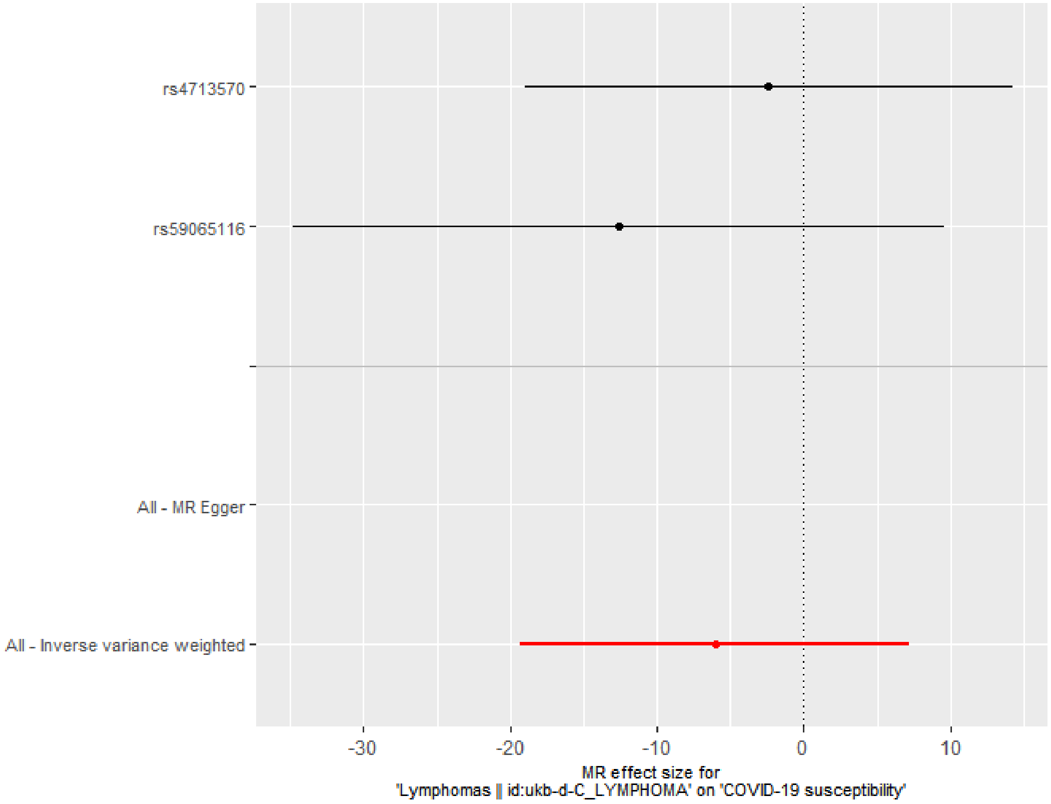

Figure S3O. The effect of each SNP in lymphoma on COVID-19 susceptibility.

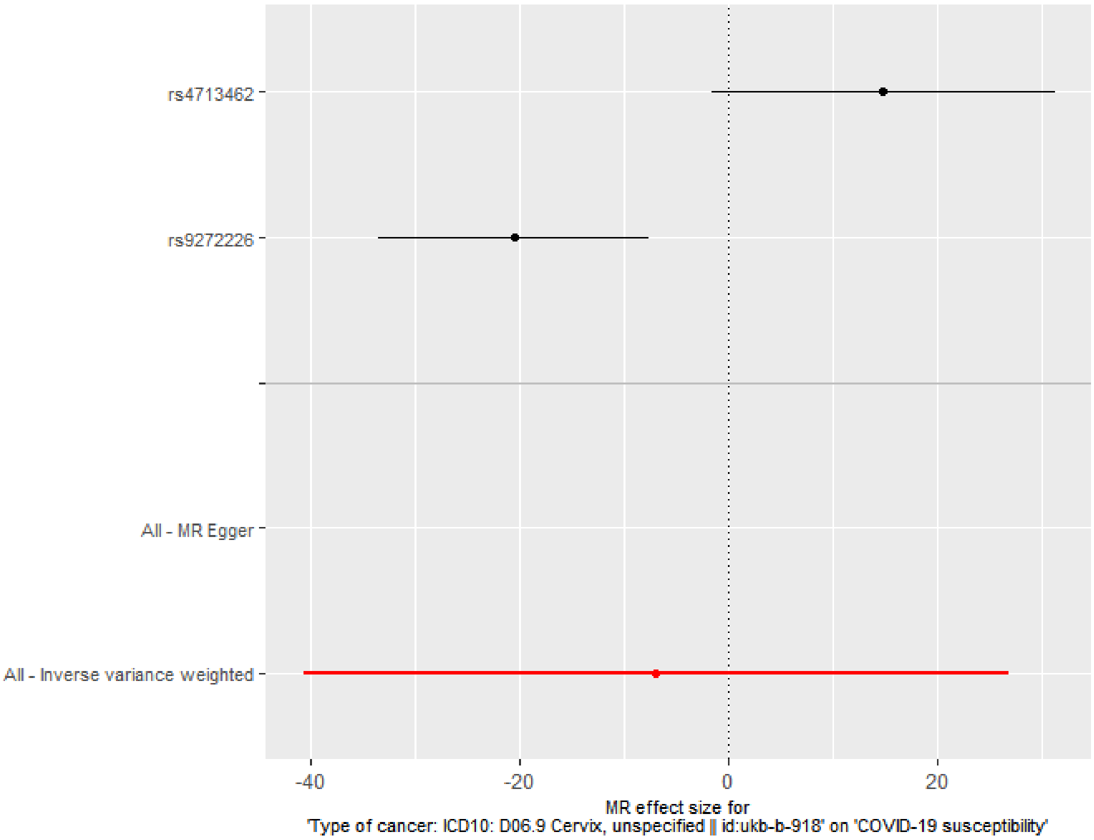

Figure S3P. The effect of each SNP in cervix cancer on COVID-19 susceptibility.

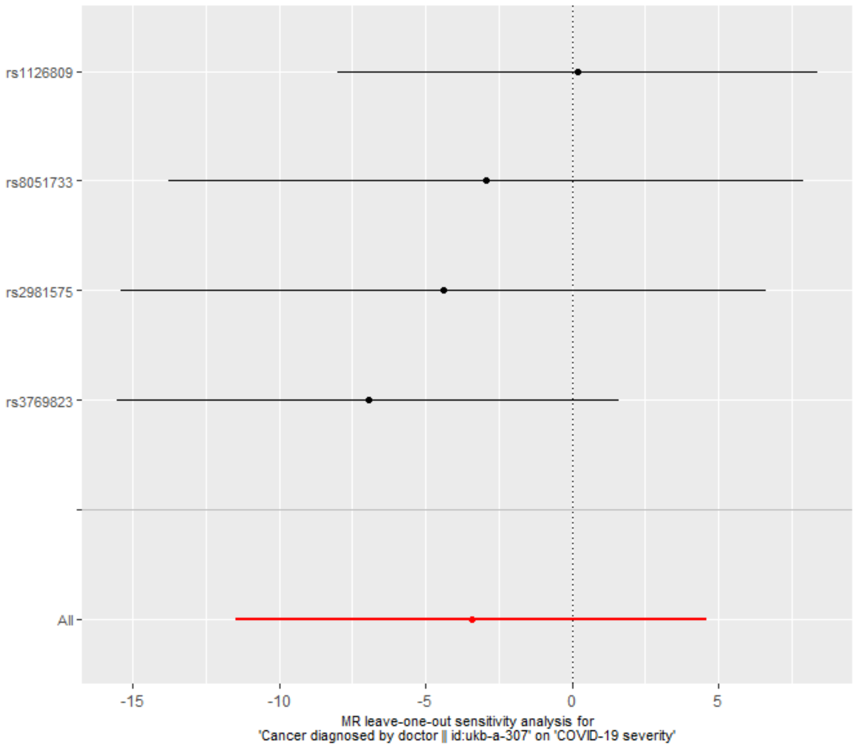

Figure S4A. The leave-one-out sensitivity analysis of the causal effect of overall cancer on COVID-19 severity.

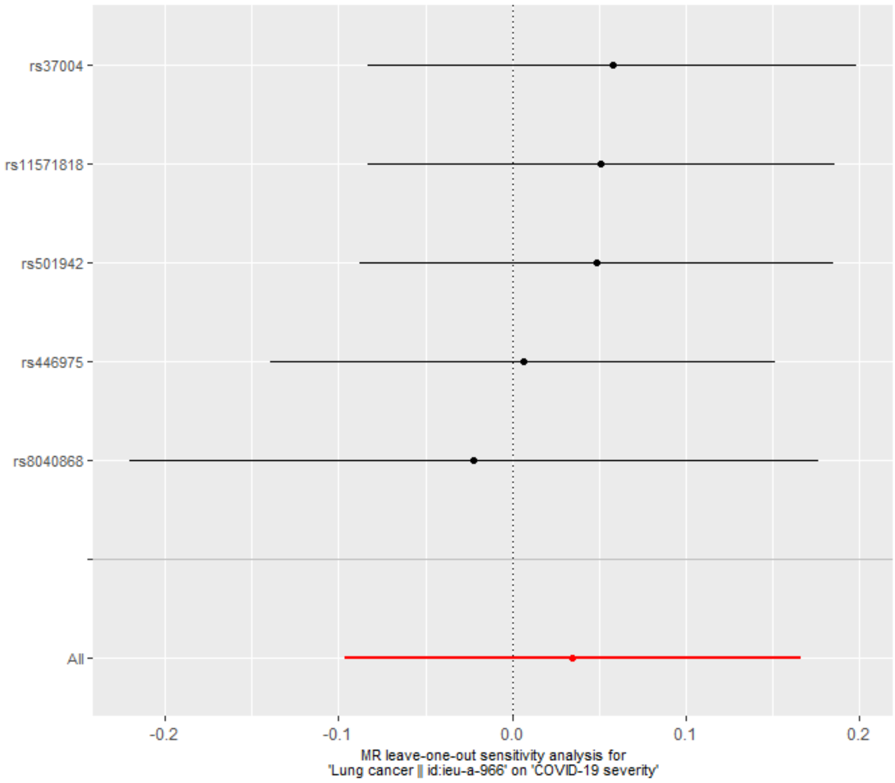

Figure S4B. The leave-one-out sensitivity analysis of the causal effect of lung cancer on COVID-19 severity.

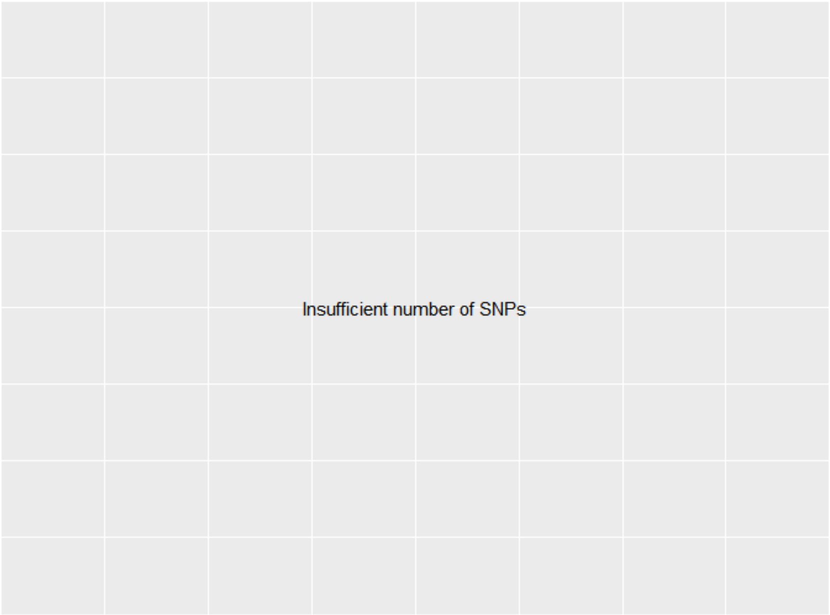

Figure S4C. The leave-one-out sensitivity analysis of the causal effect of squamous cell lung cancer on COVID-19 severity.

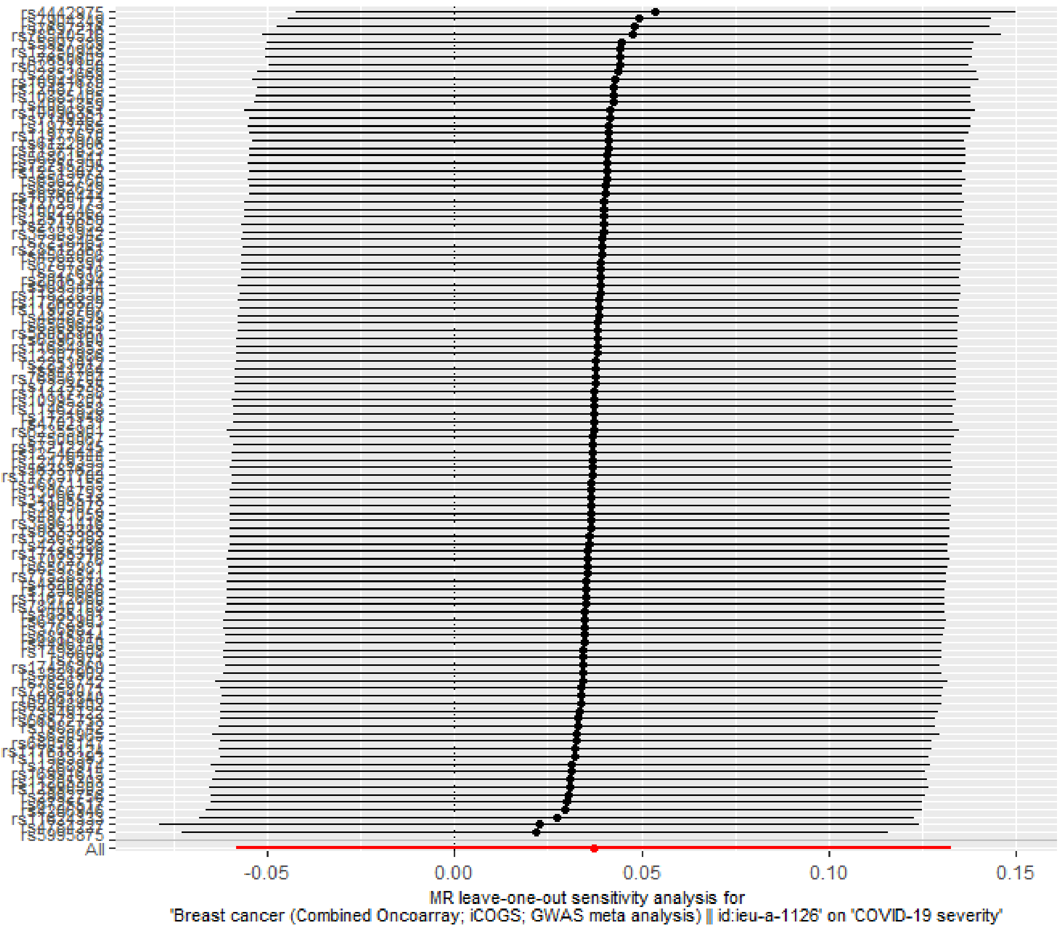

Figure S4D. The leave-one-out sensitivity analysis of the causal effect of breast cancer on COVID-19 severity.

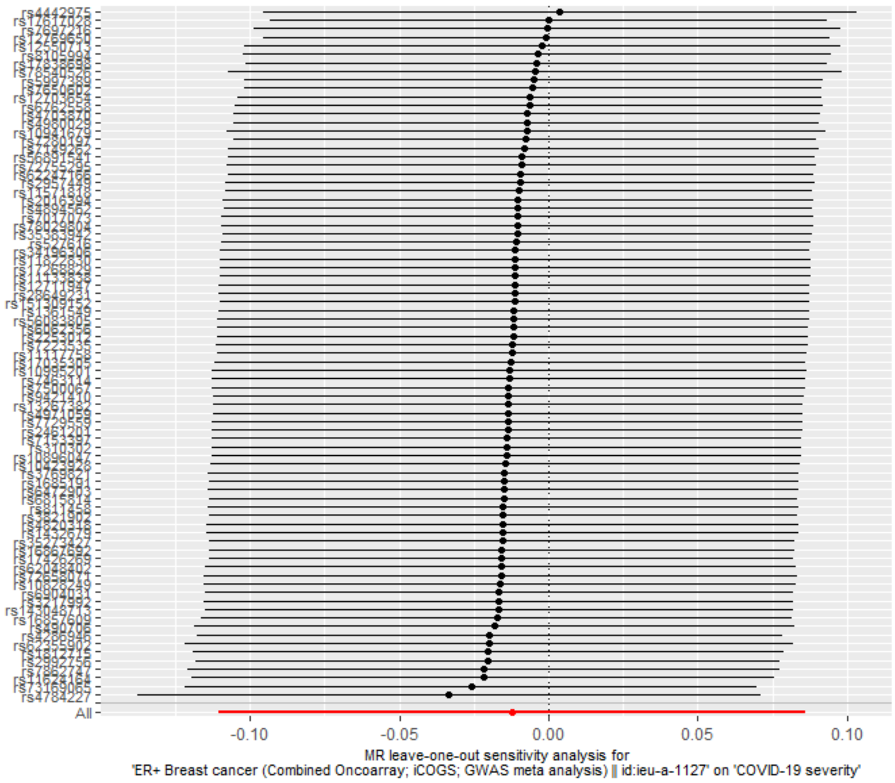

Figure S4E. The leave-one-out sensitivity analysis of the causal effect of ER+ breast cancer on COVID-19 severity.

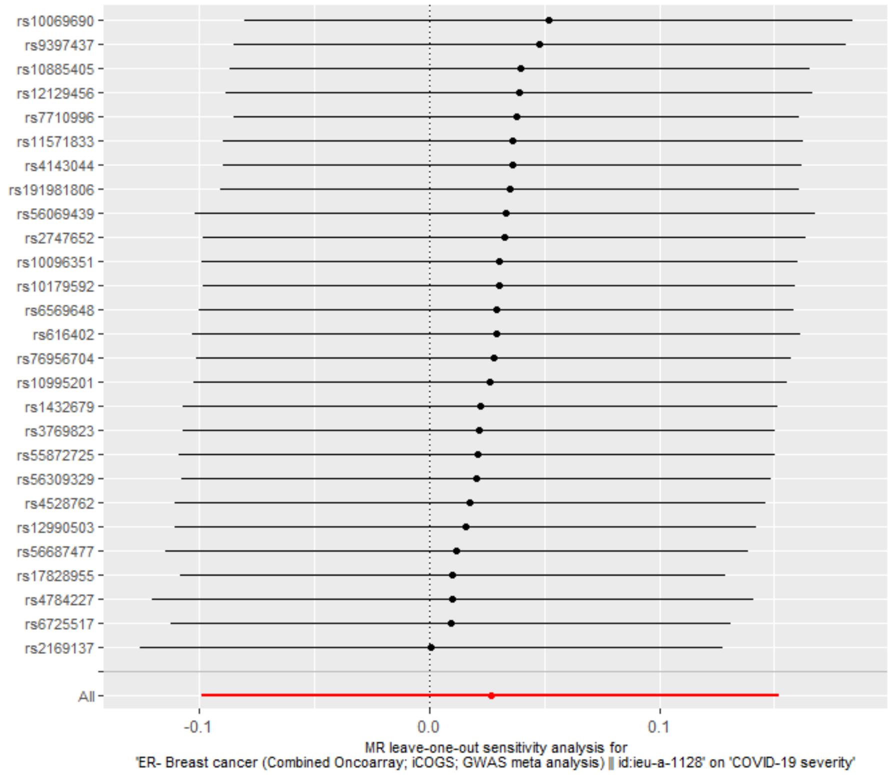

Figure S4F. The leave-one-out sensitivity analysis of the causal effect of ER- breast cancer on COVID-19 severity.

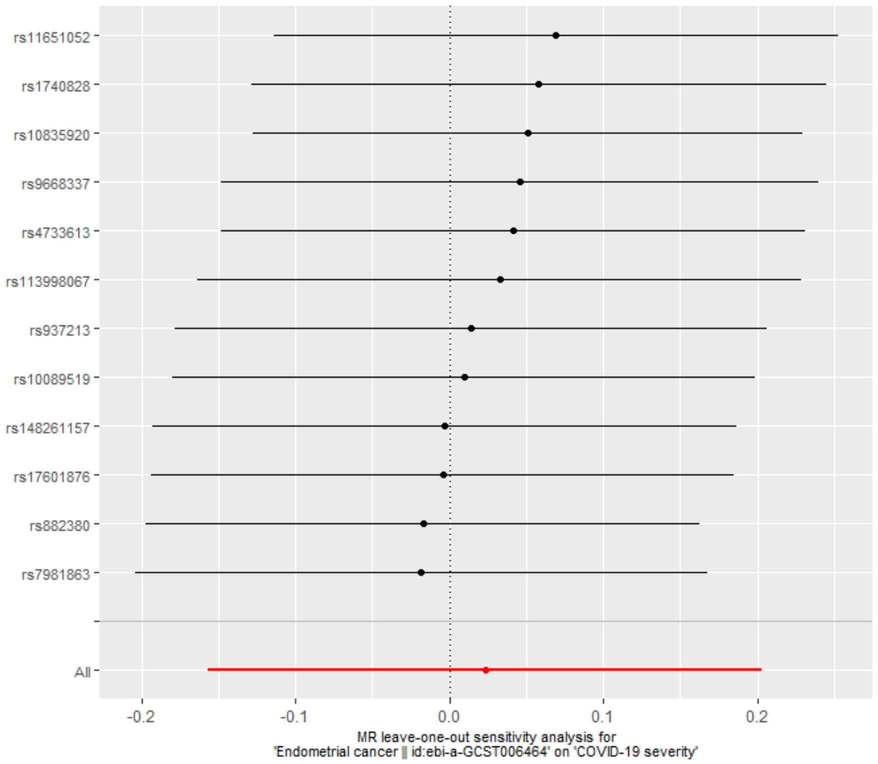

Figure S4G. The leave-one-out sensitivity analysis of the causal effect of endometrial cancer on COVID-19 severity.

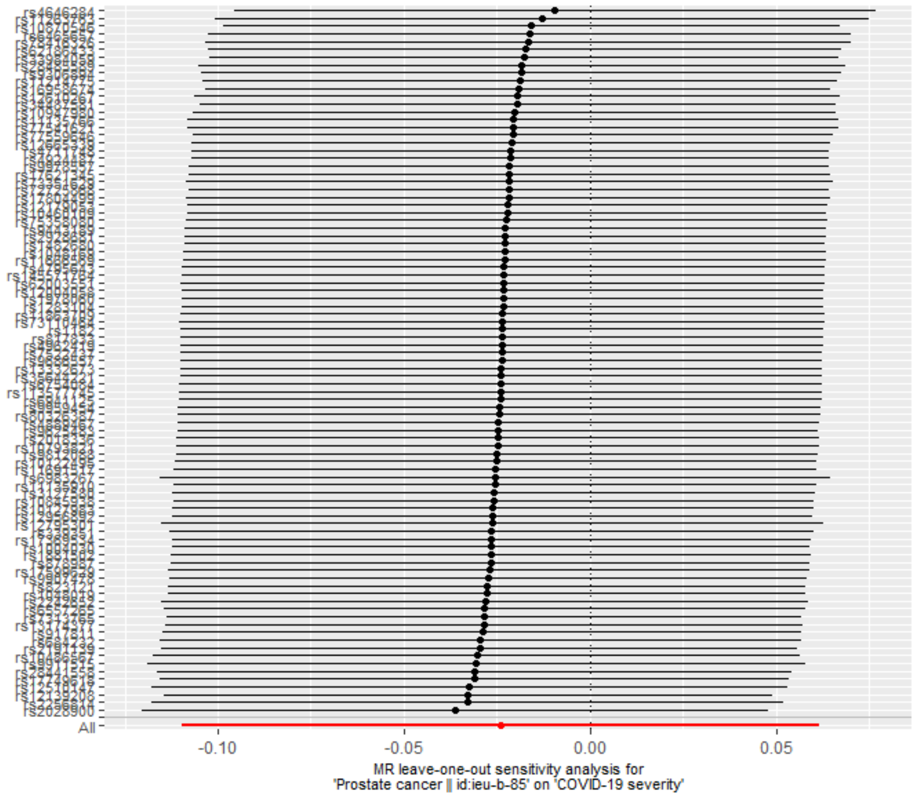

Figure S4H. The leave-one-out sensitivity analysis of the causal effect of prostate cancer on COVID-19 severity.

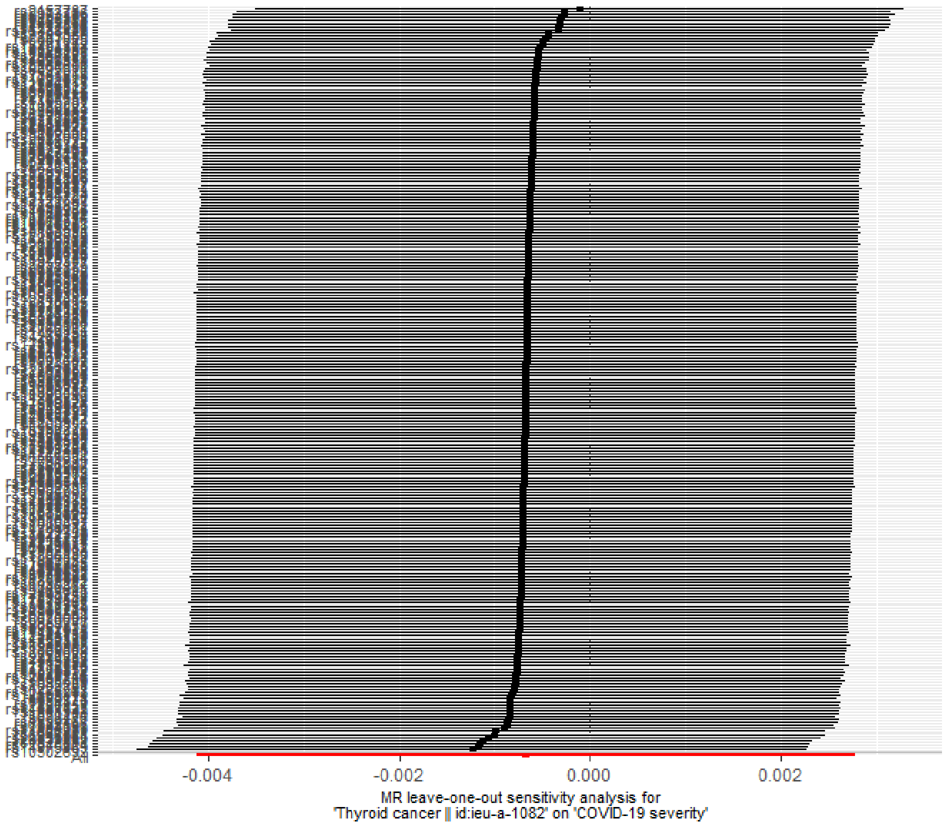

Figure S4I. The leave-one-out sensitivity analysis of the causal effect of thyroid cancer on COVID-19 severity.

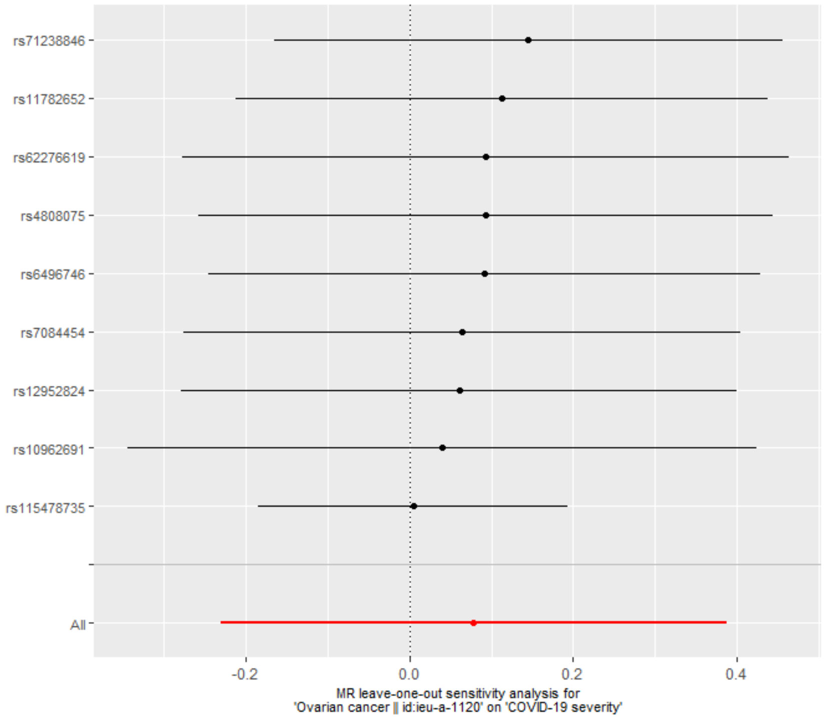

Figure S4J. The leave-one-out sensitivity analysis of the causal effect of ovarian cancer on COVID-19 severity.

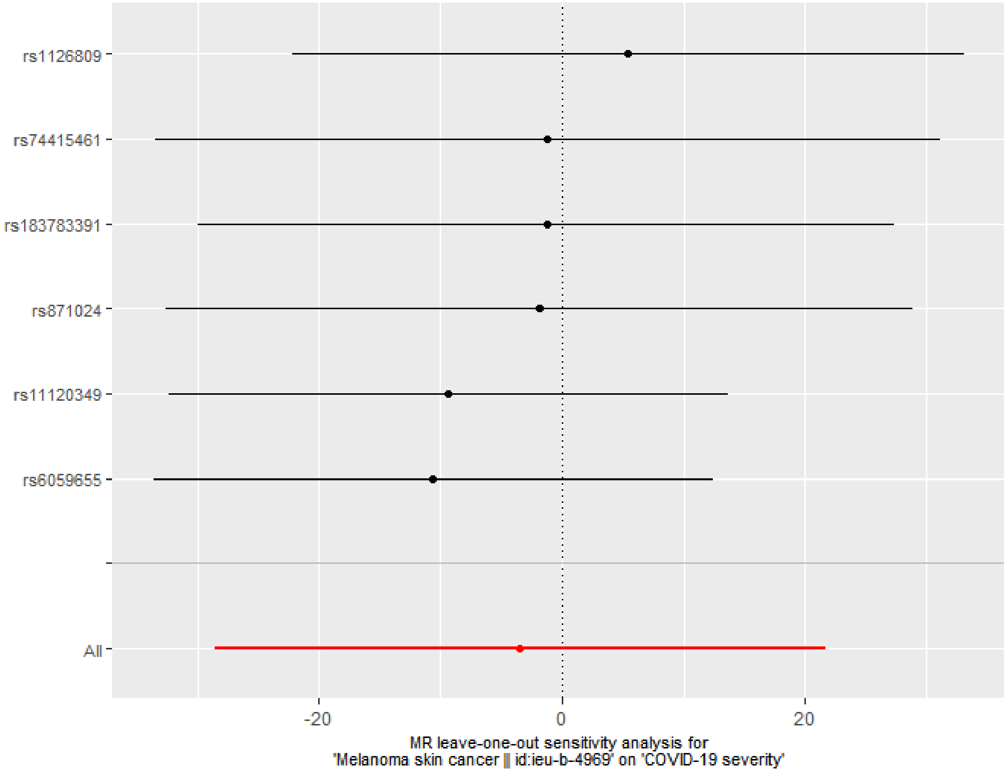

Figure S4K. The leave-one-out sensitivity analysis of the causal effect of melanoma on COVID-19 severity.

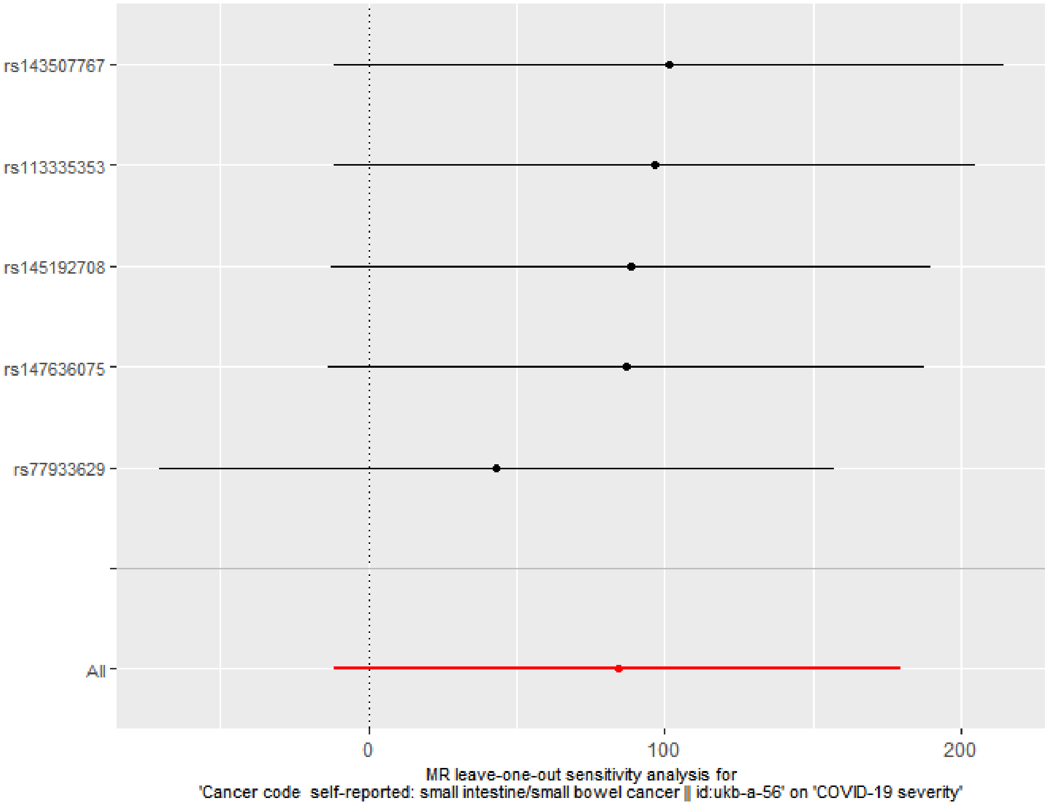

Figure S4L. The leave-one-out sensitivity analysis of the causal effect of small bowel cancer on COVID-19 severity.

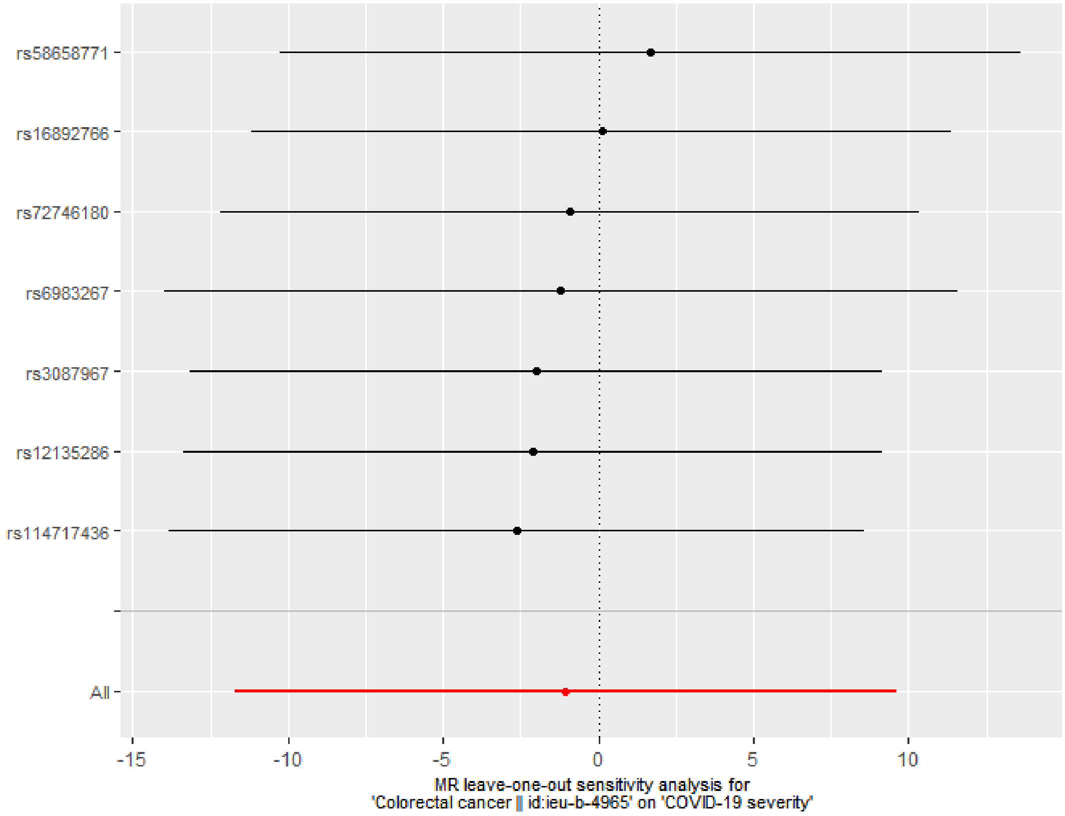

Figure S4M. The leave-one-out sensitivity analysis of the causal effect of colorectal cancer on COVID-19 severity.

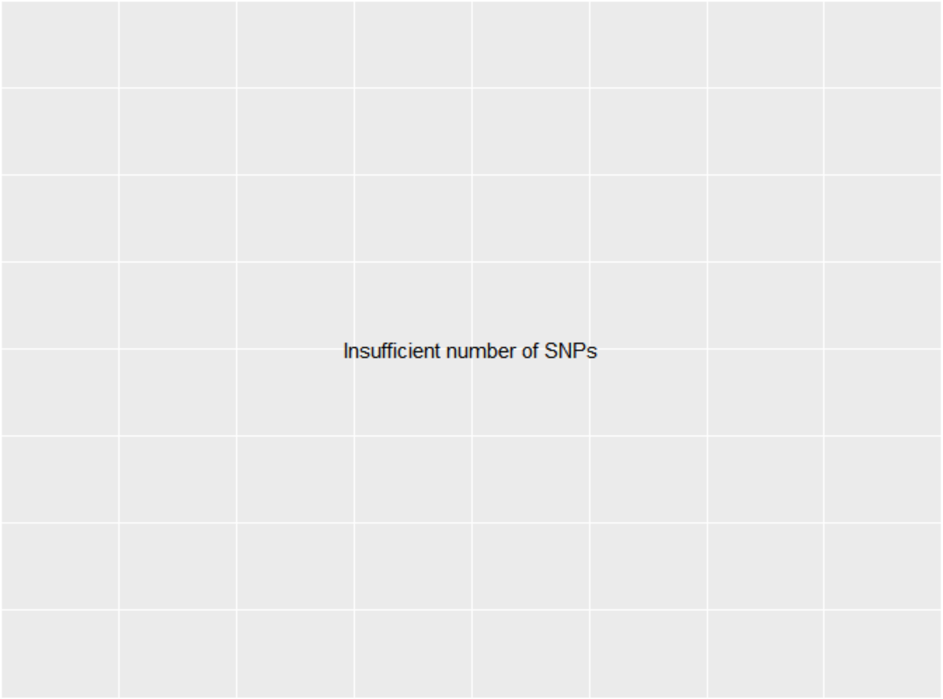

Figure S4N. The leave-one-out sensitivity analysis of the causal effect of oropharyngeal cancer on COVID-19 severity.

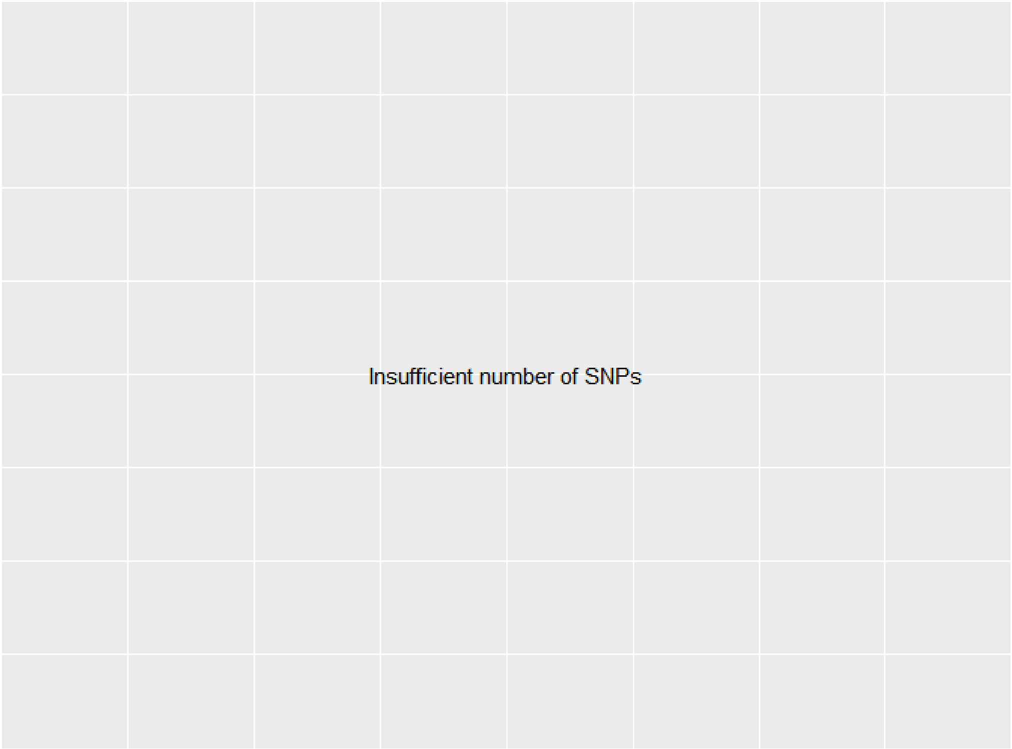

Figure S4O. The leave-one-out sensitivity analysis of the causal effect of lymphoma on COVID-19 severity.

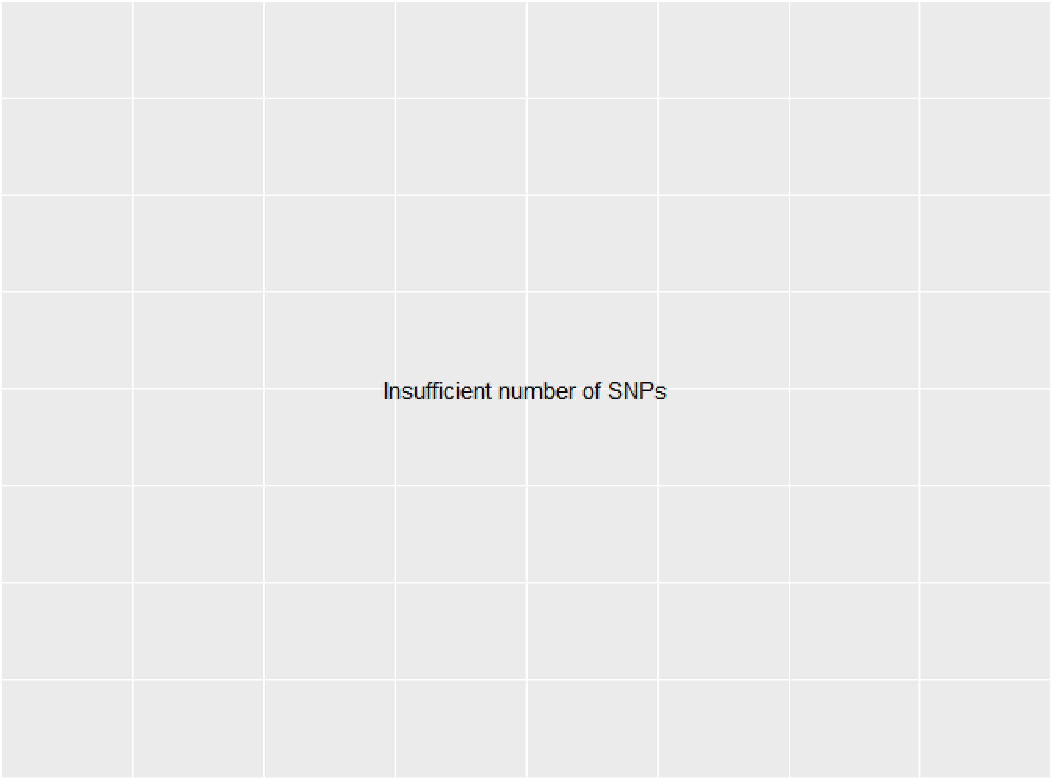

Figure S4P. The leave-one-out sensitivity analysis of the causal effect of cervix cancer on COVID-19 severity.

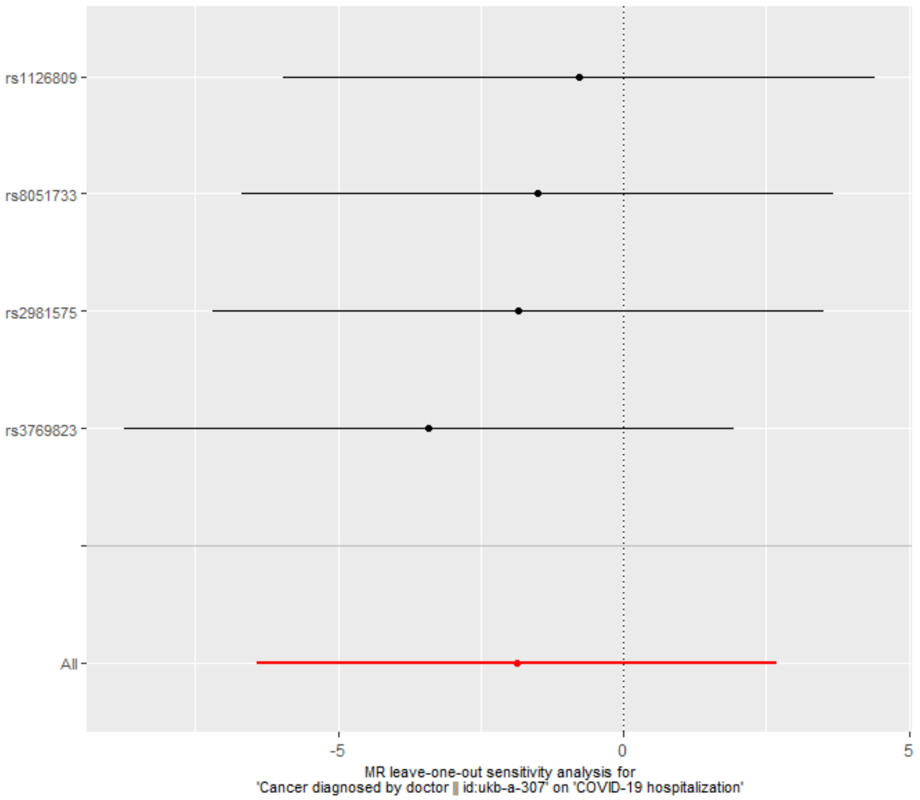

Figure S5A. The leave-one-out sensitivity analysis of the causal effect of overall cancer on COVID-19 hospitalization.

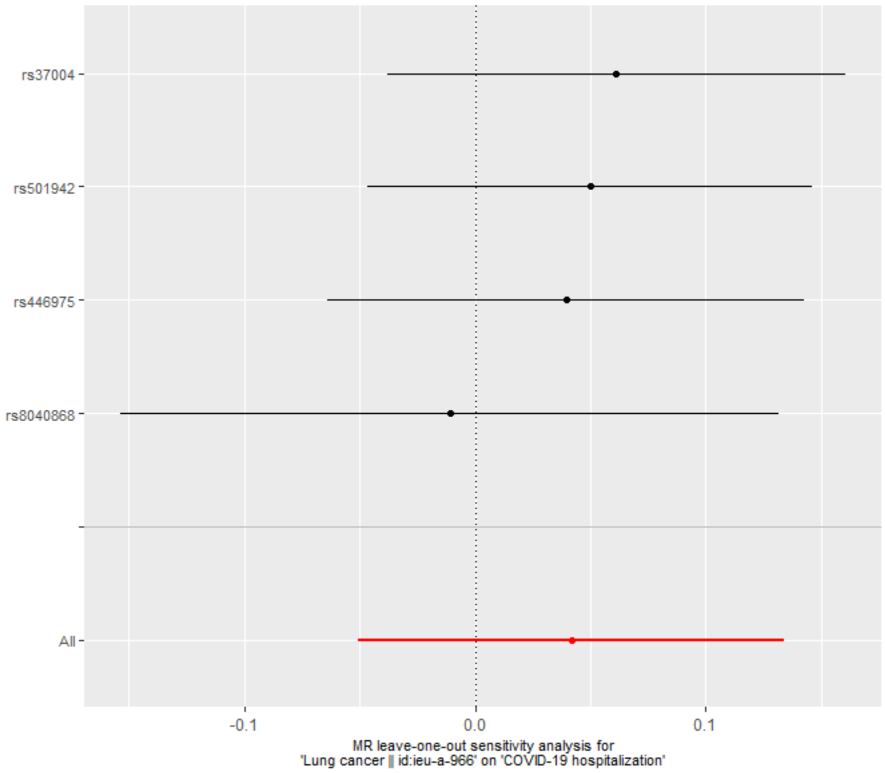

Figure S5B. The leave-one-out sensitivity analysis of the causal effect of lung cancer on COVID-19 hospitalization.

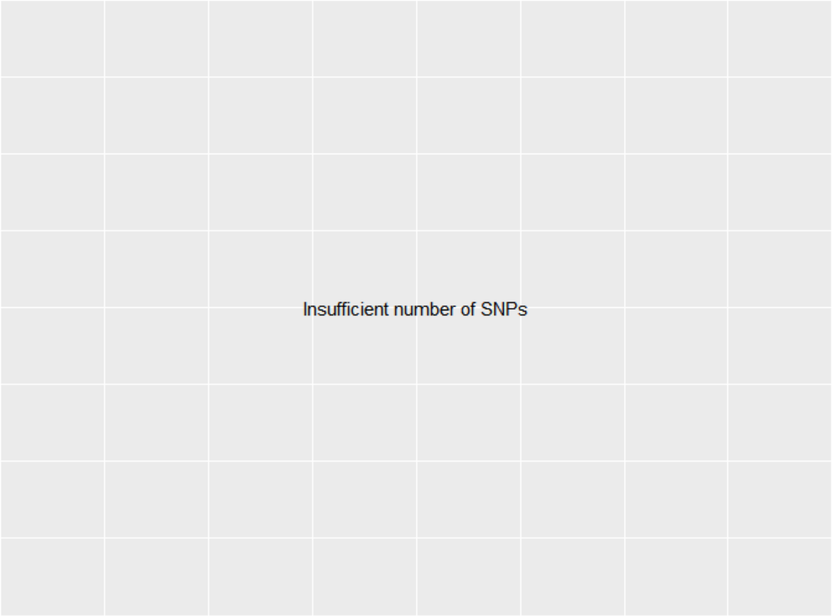

Figure S5C. The leave-one-out sensitivity analysis of the causal effect of squamous cell lung cancer on COVID-19 hospitalization.

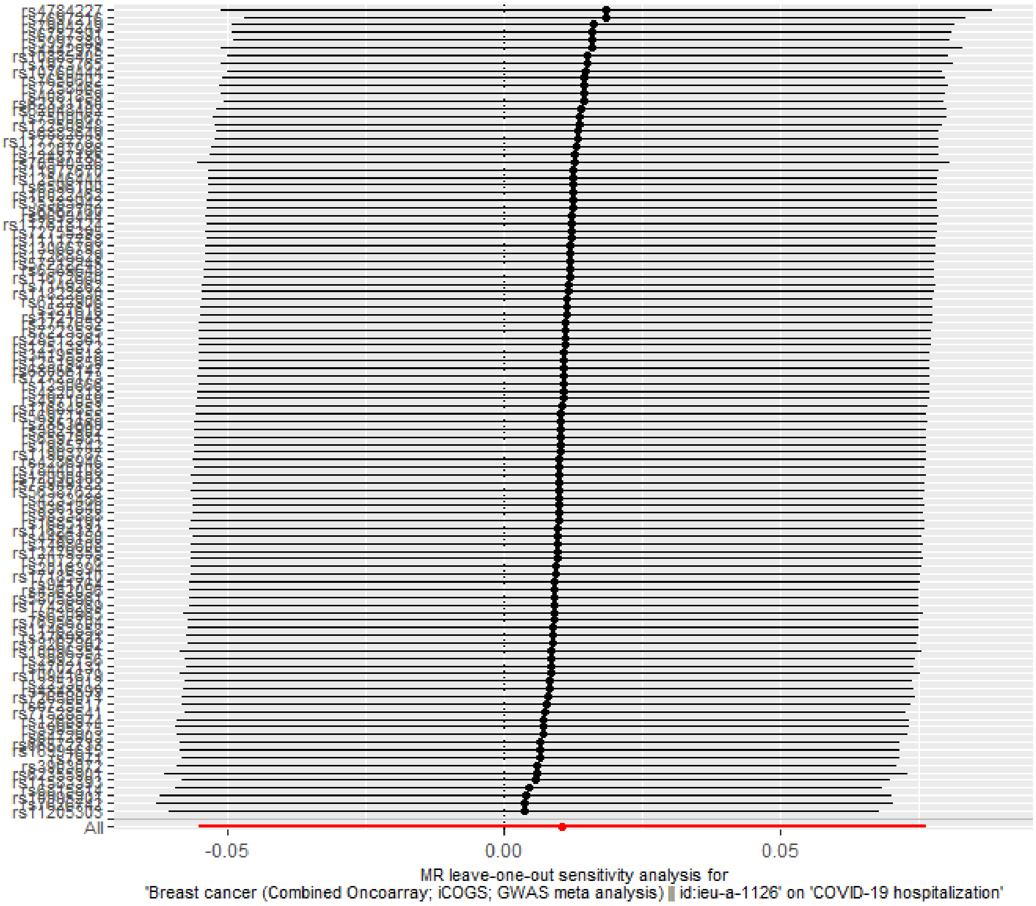

Figure S5D. The leave-one-out sensitivity analysis of the causal effect of breast cancer on COVID-19 hospitalization.

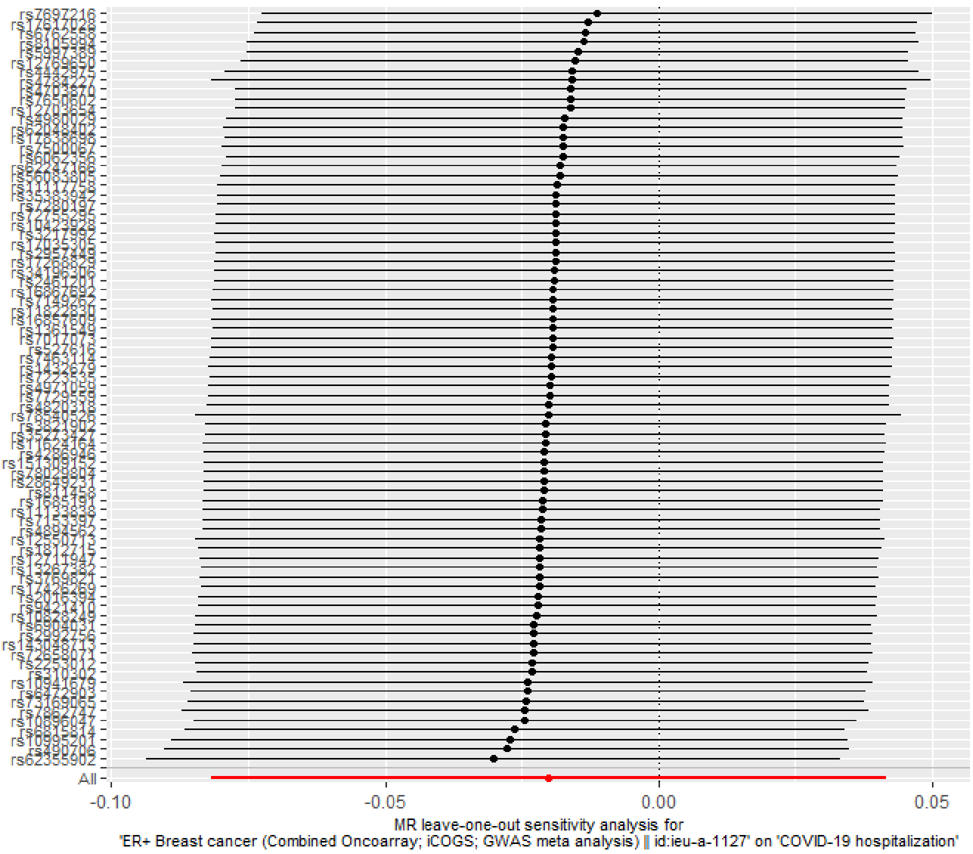

Figure S5E. The leave-one-out sensitivity analysis of the causal effect of ER+ breast cancer on COVID-19 hospitalization.

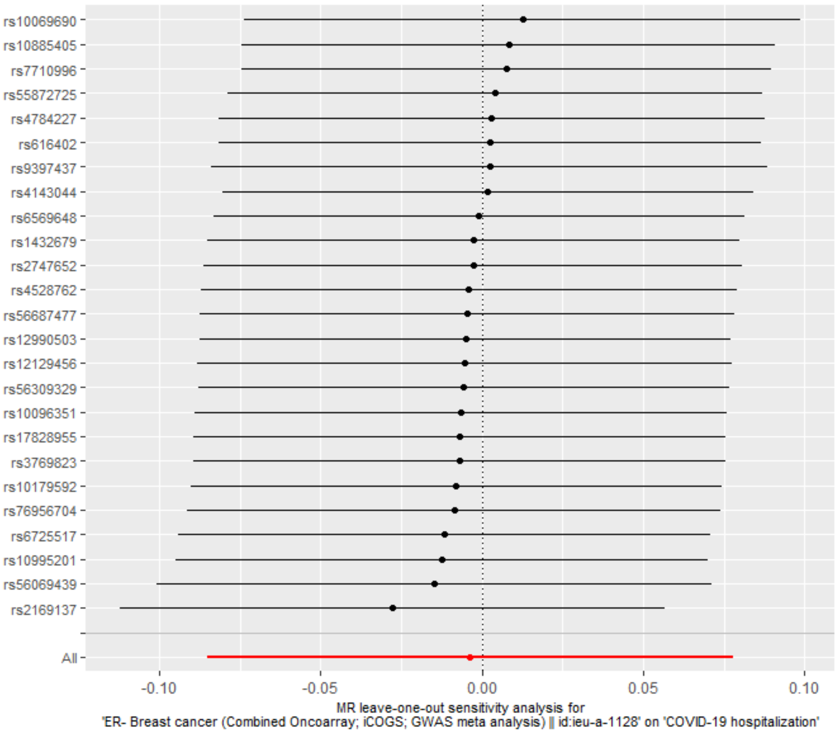

Figure S5F. The leave-one-out sensitivity analysis of the causal effect of ER- breast cancer on COVID-19 hospitalization.

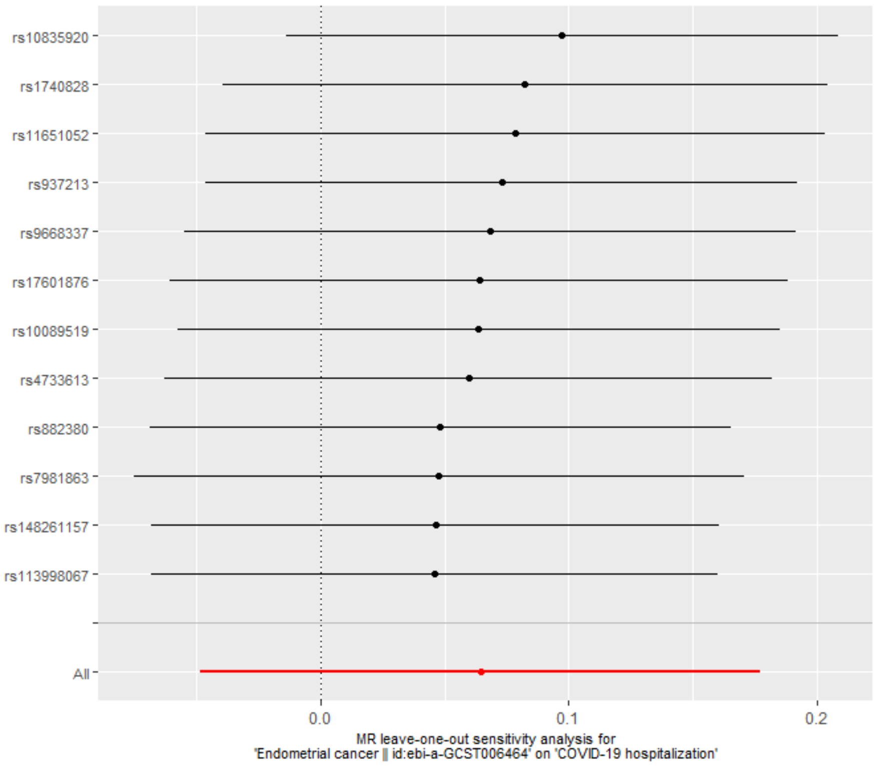

Figure S5G. The leave-one-out sensitivity analysis of the causal effect of endometrial cancer on COVID-19 hospitalization.

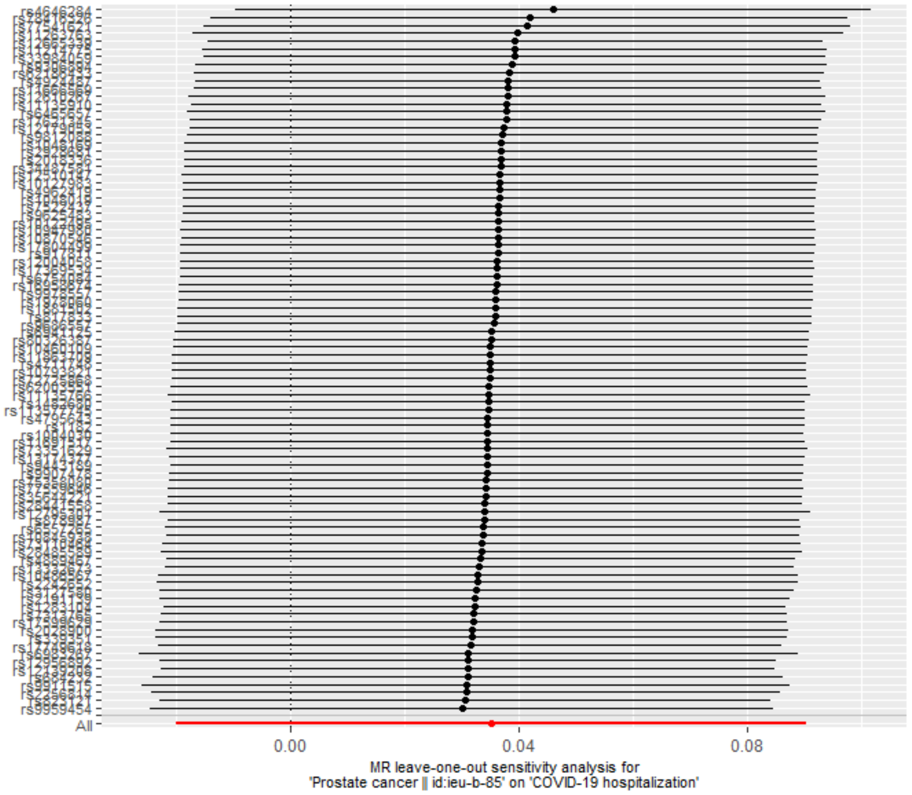

Figure S5H. The leave-one-out sensitivity analysis of the causal effect of prostate cancer on COVID-19 hospitalization.

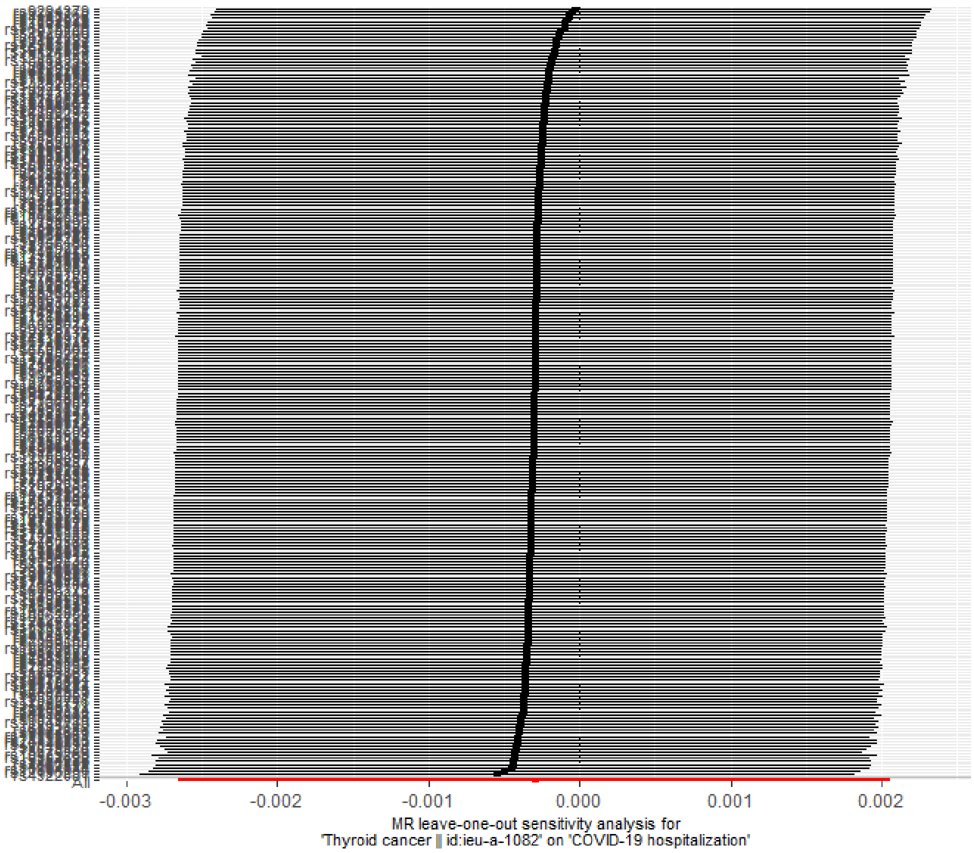

Figure S5I. The leave-one-out sensitivity analysis of the causal effect of thyroid cancer on COVID-19 hospitalization.

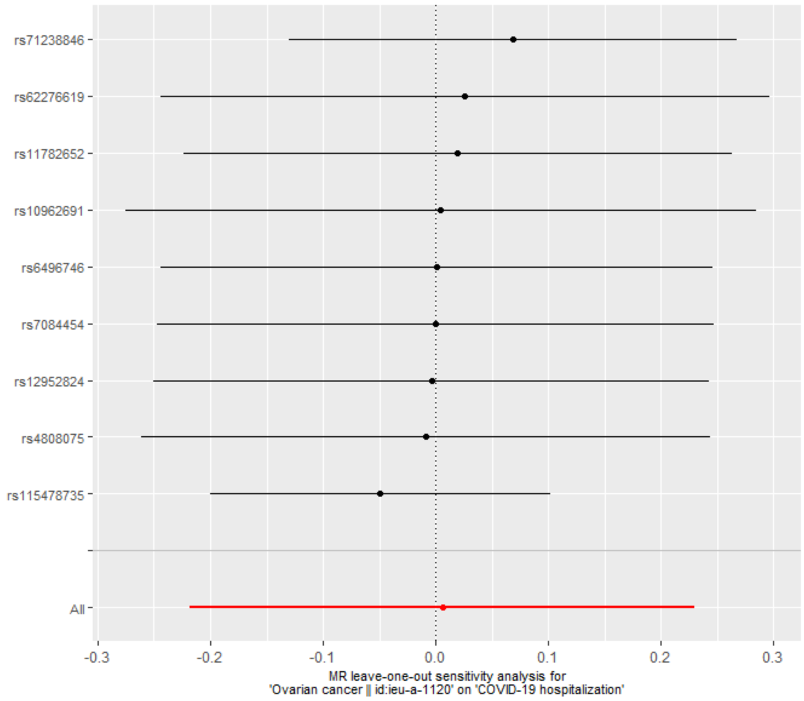

Figure S5J. The leave-one-out sensitivity analysis of the causal effect of ovarian cancer on COVID-19 hospitalization.

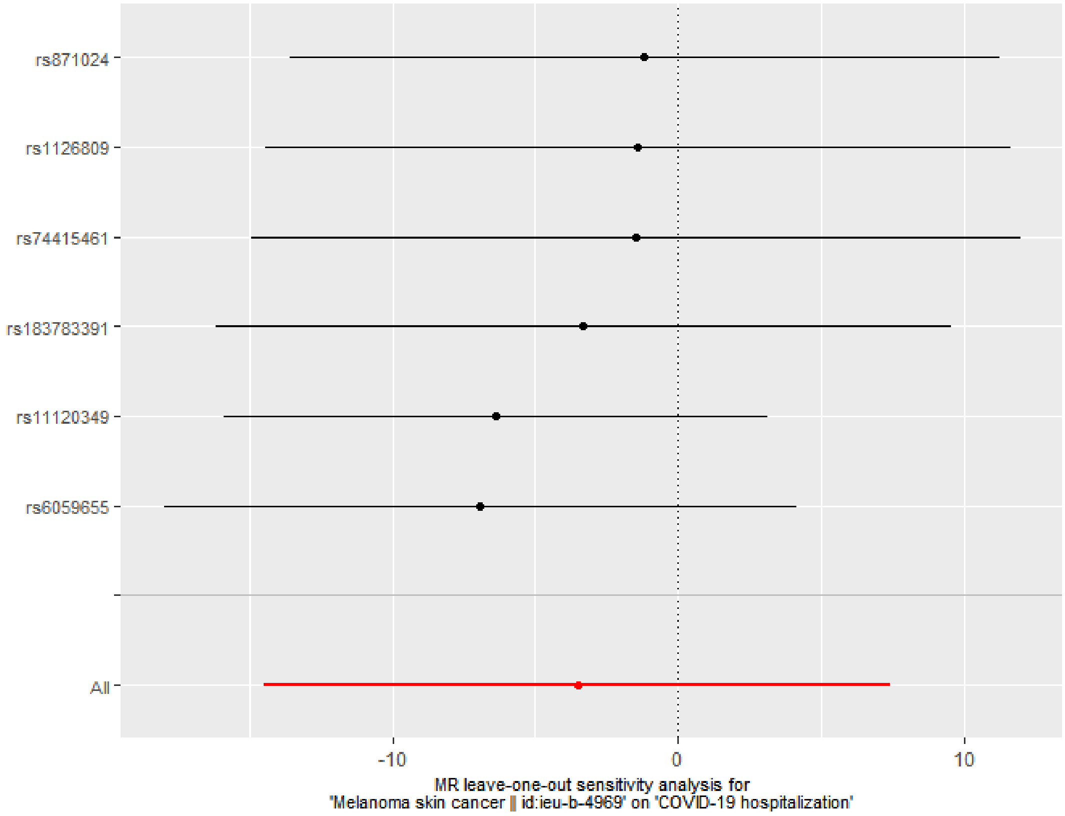

Figure S5K. The leave-one-out sensitivity analysis of the causal effect of melanoma on COVID-19 hospitalization.

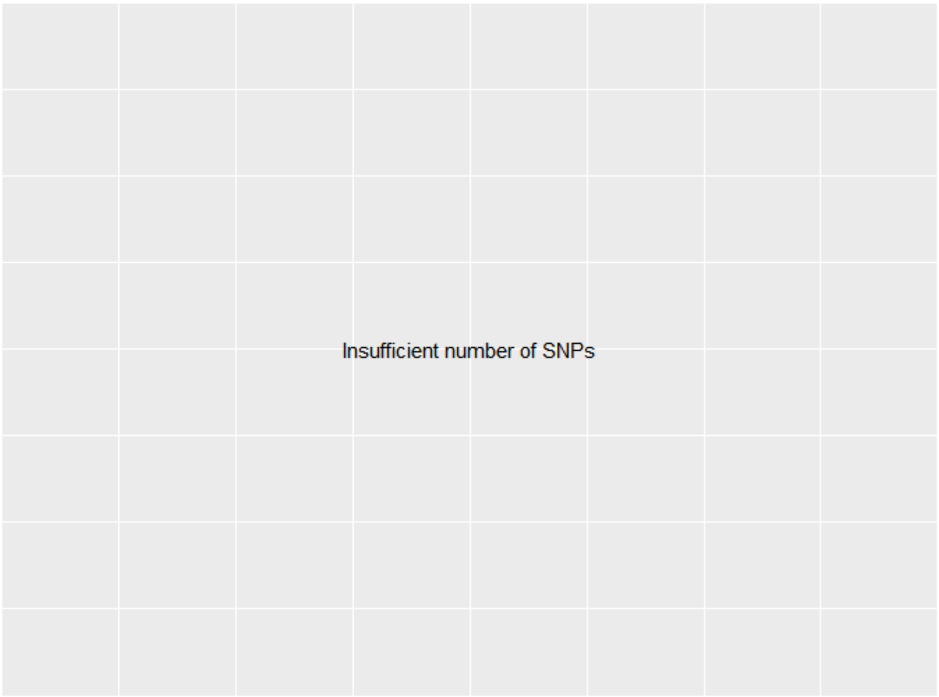

Figure S5L. The leave-one-out sensitivity analysis of the causal effect of small bowel cancer on COVID-19 hospitalization.

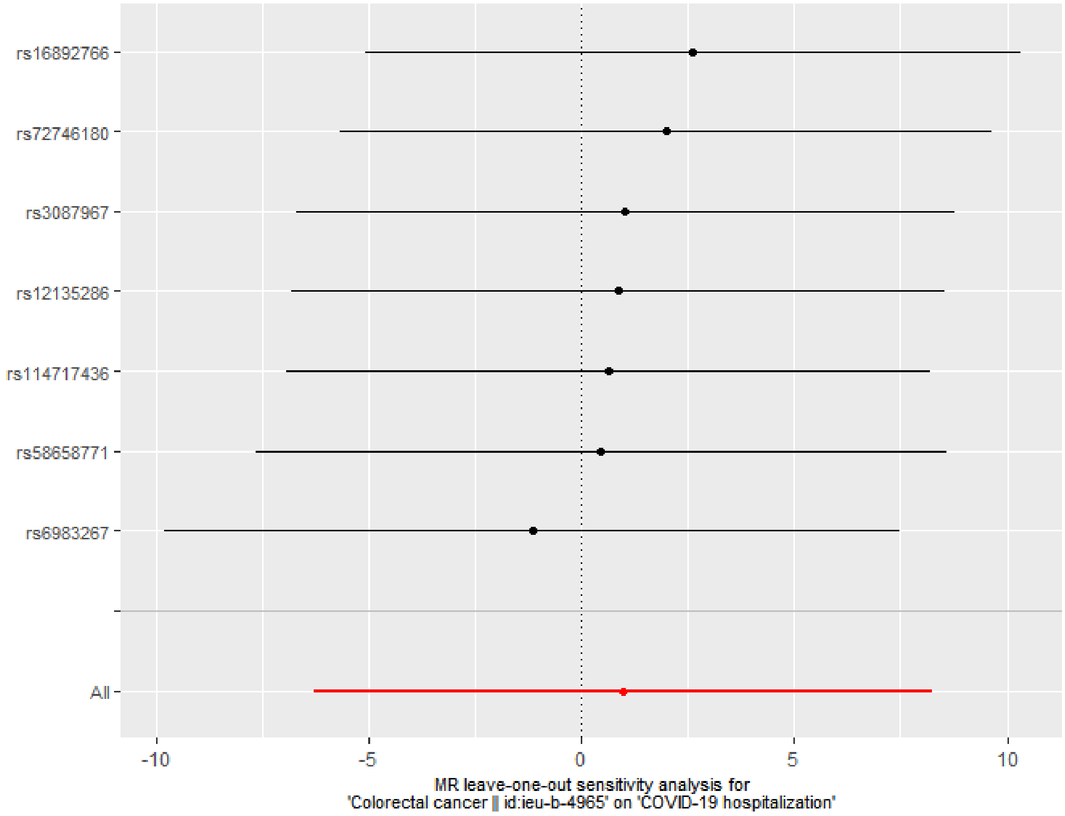

Figure S5L. The leave-one-out sensitivity analysis of the causal effect of colorectal cancer on COVID-19 hospitalization.

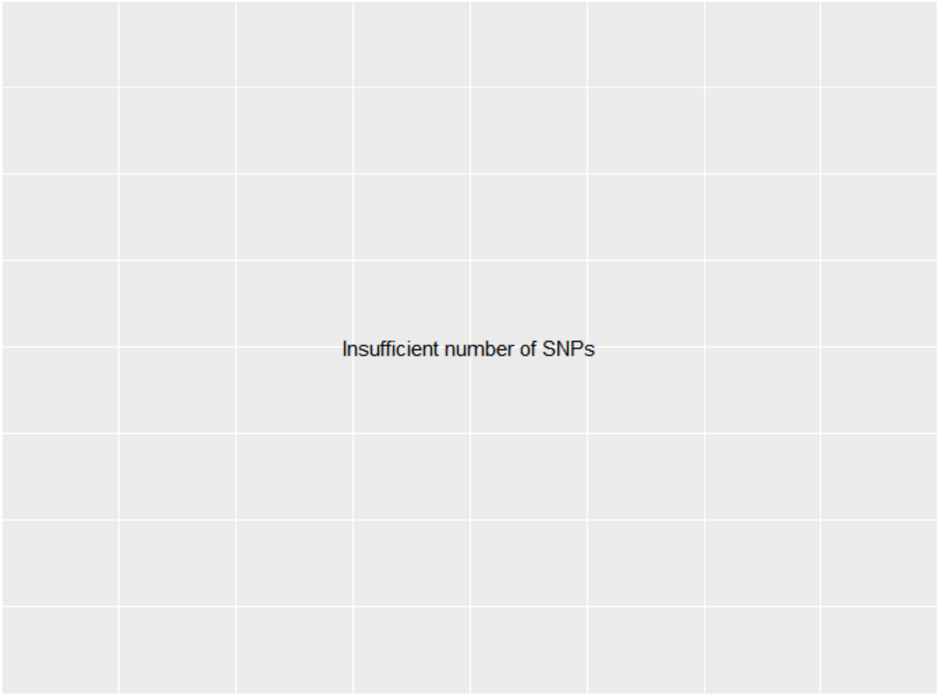

Figure S5N. The leave-one-out sensitivity analysis of the causal effect of oropharyngeal cancer on COVID-19 hospitalization.

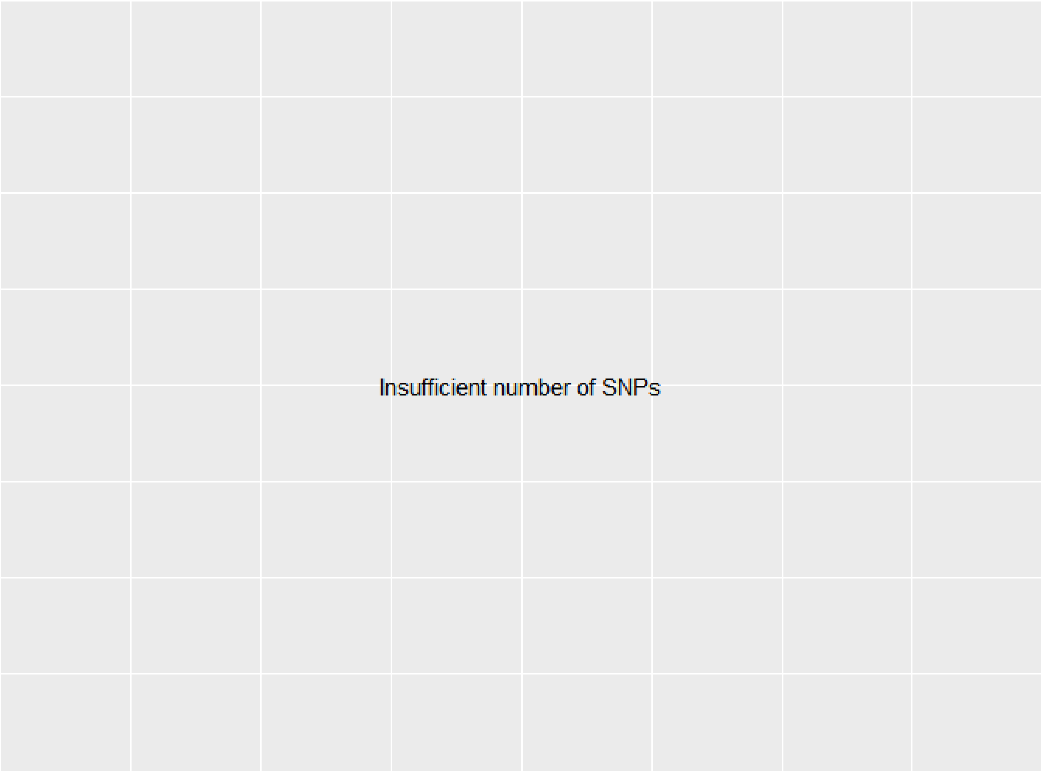

Figure S5O. The leave-one-out sensitivity analysis of the causal effect of lymphoma on COVID-19 hospitalization.

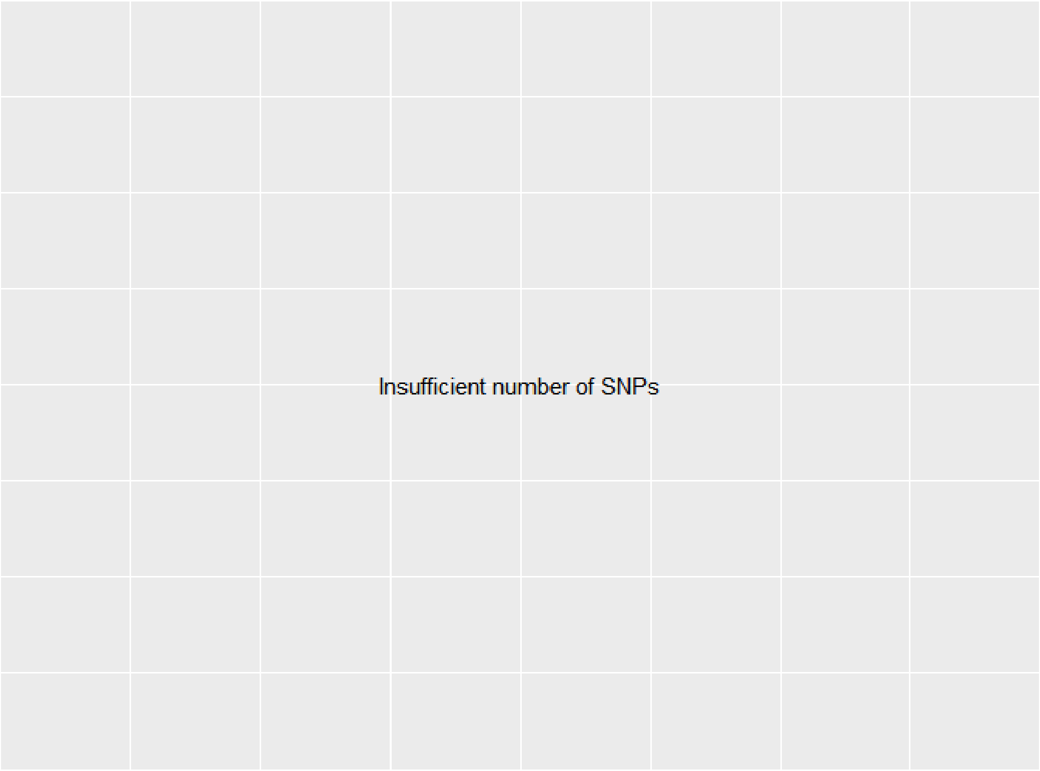

Figure S5P. The leave-one-out sensitivity analysis of the causal effect of cervix cancer on COVID-19 hospitalization.

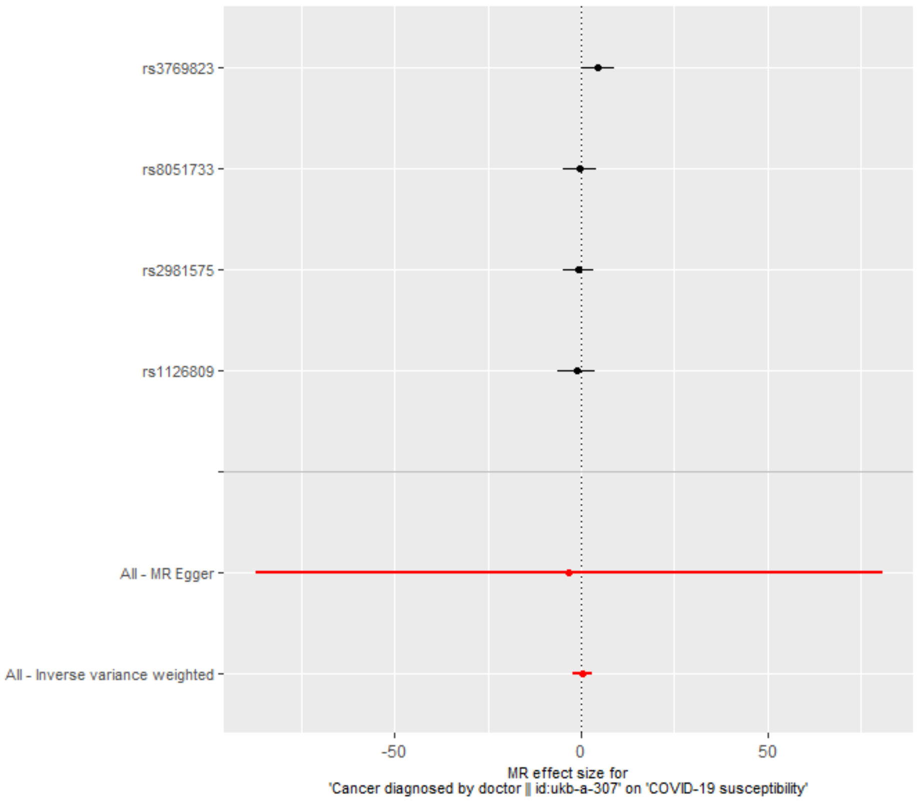

Figure S6A. The leave-one-out sensitivity analysis of the causal effect of overall cancer on COVID-19 susceptibility.

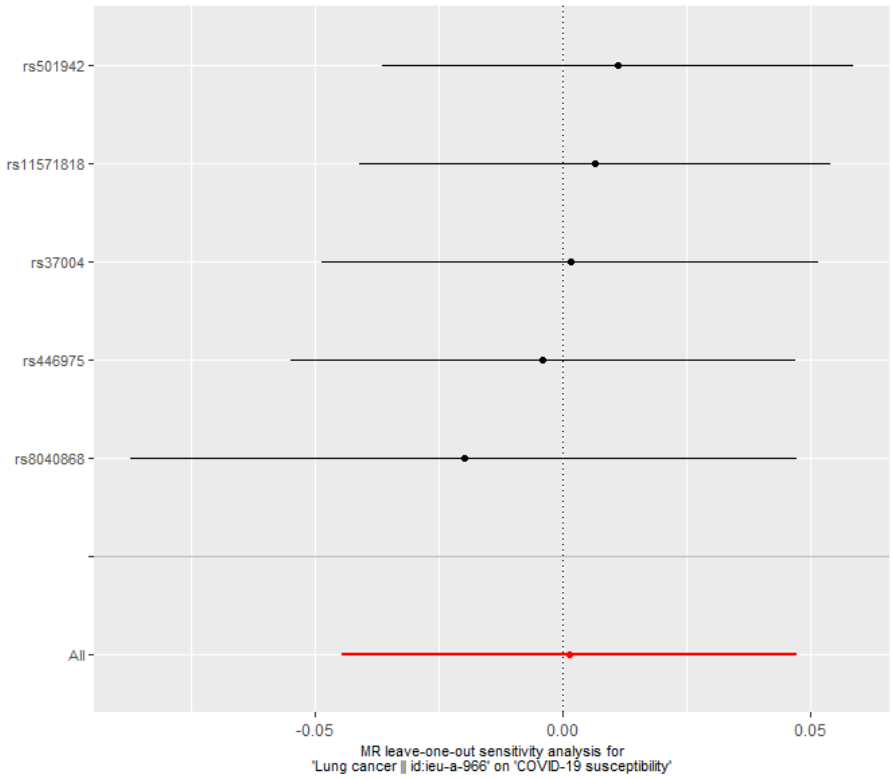

Figure S6B. The leave-one-out sensitivity analysis of the causal effect of lung cancer on COVID-19 susceptibility.

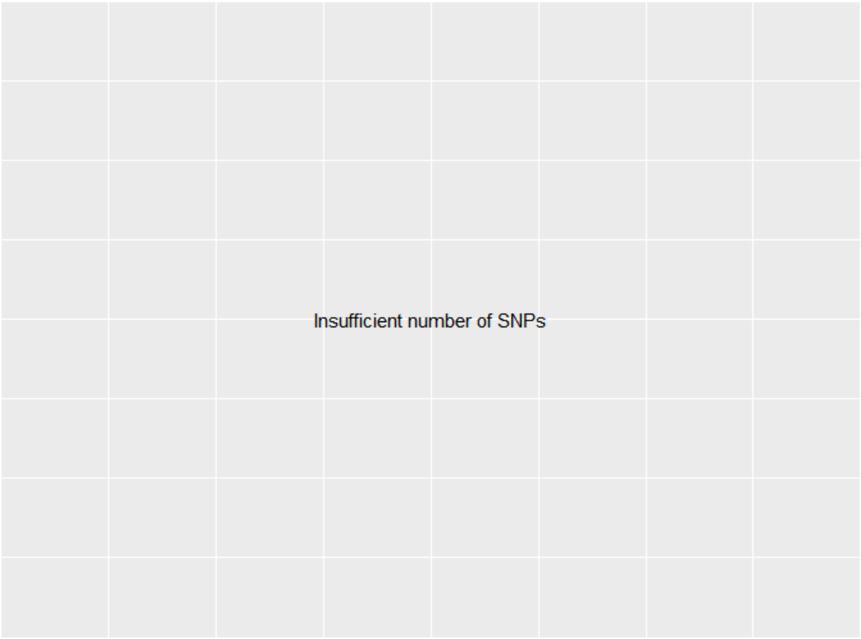

Figure S6C. The leave-one-out sensitivity analysis of the causal effect of squamous cell lung cancer on COVID-19 susceptibility.

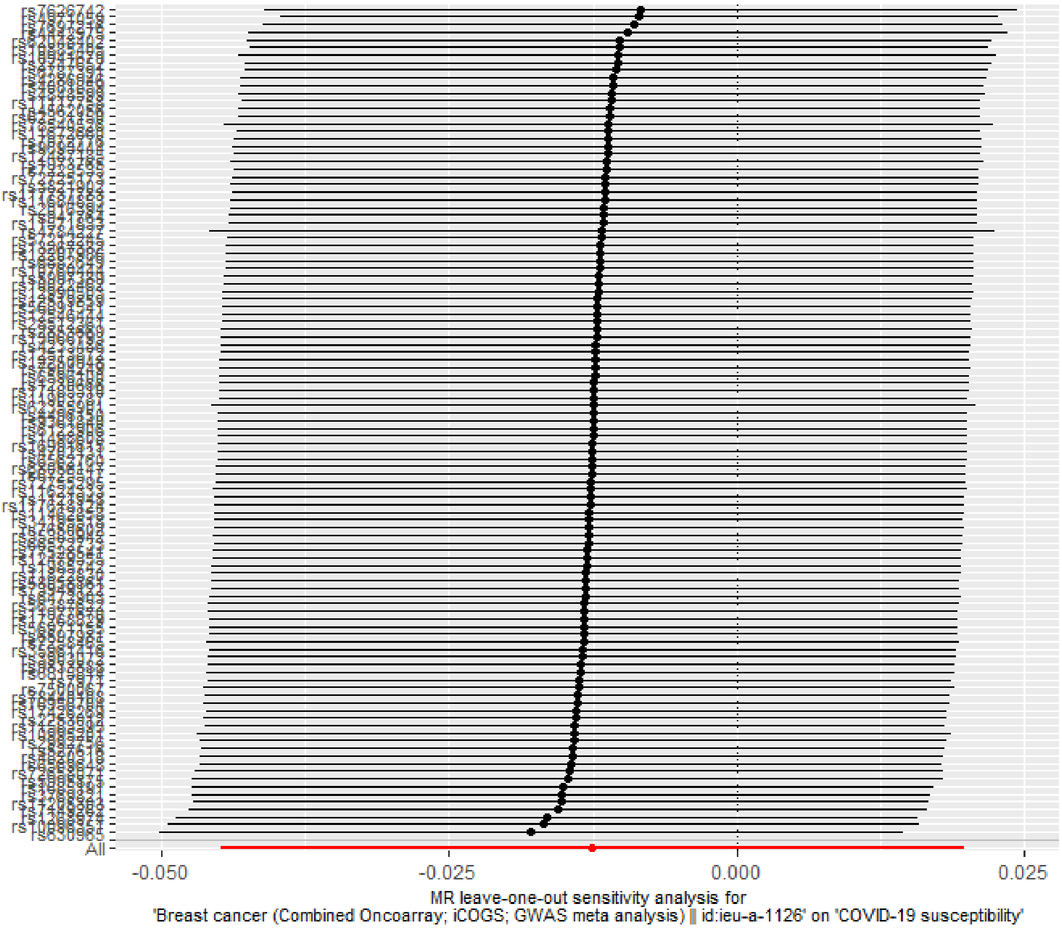

Figure S6D. The leave-one-out sensitivity analysis of the causal effect of breast cancer on COVID-19 susceptibility.

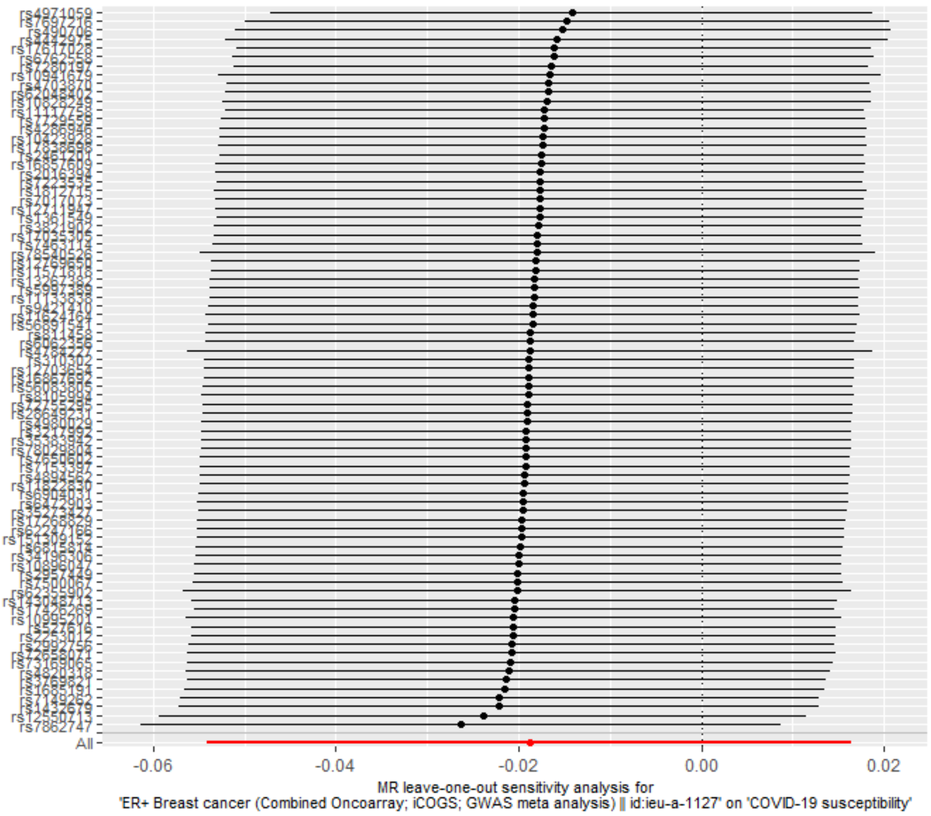

Figure S6E. The leave-one-out sensitivity analysis of the causal effect of ER+ breast cancer on COVID-19 susceptibility.

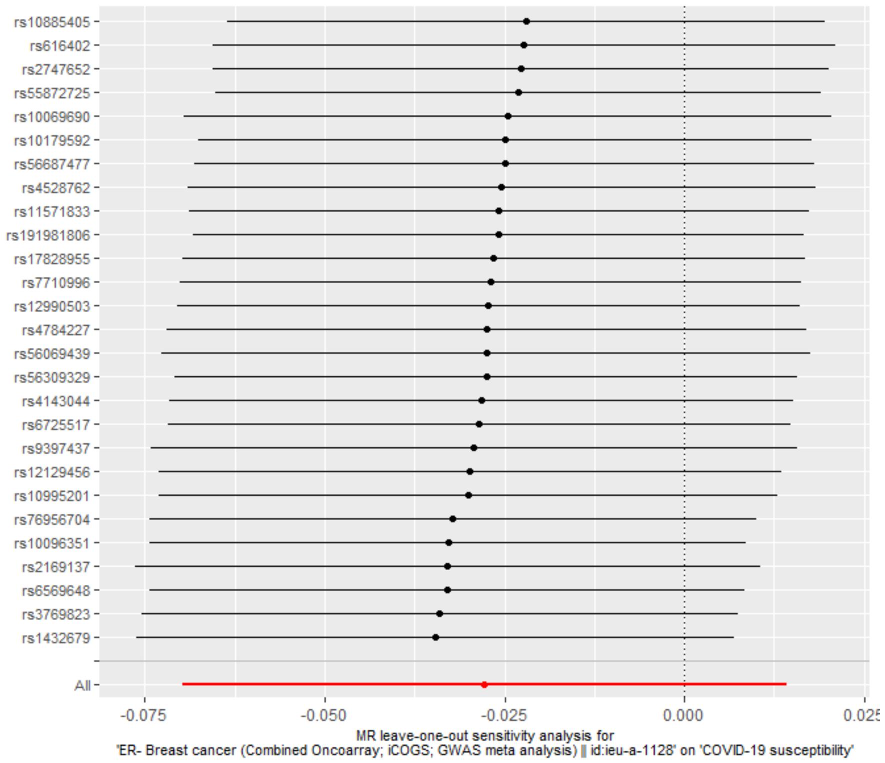

Figure S6F. The leave-one-out sensitivity analysis of the causal effect of ER- breast cancer on COVID-19 susceptibility.

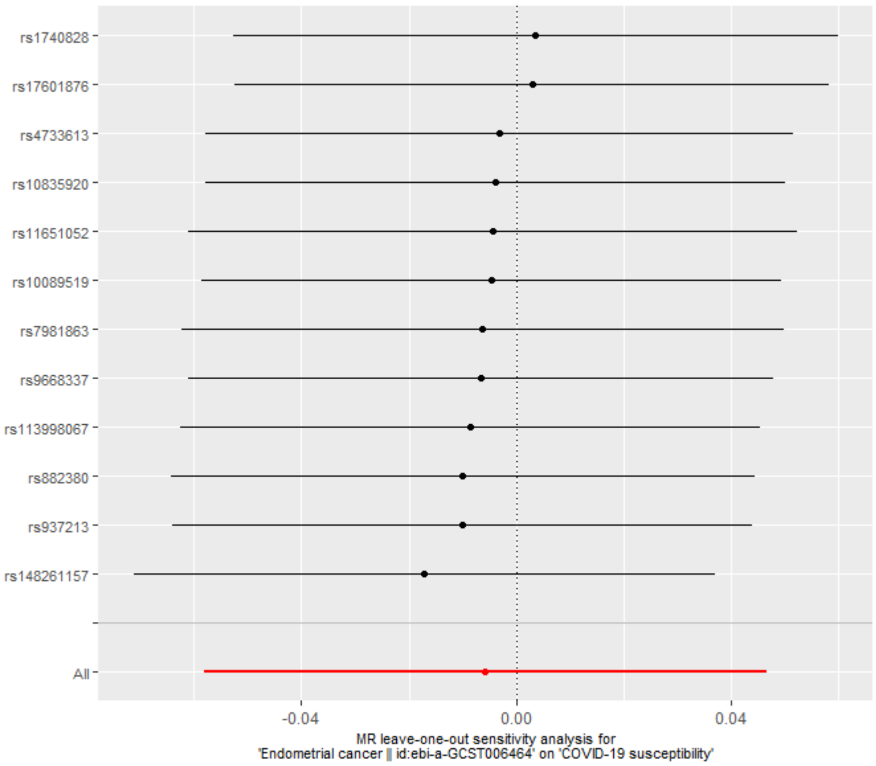

Figure S6G. The leave-one-out sensitivity analysis of the causal effect of endometrial cancer on COVID-19 susceptibility.

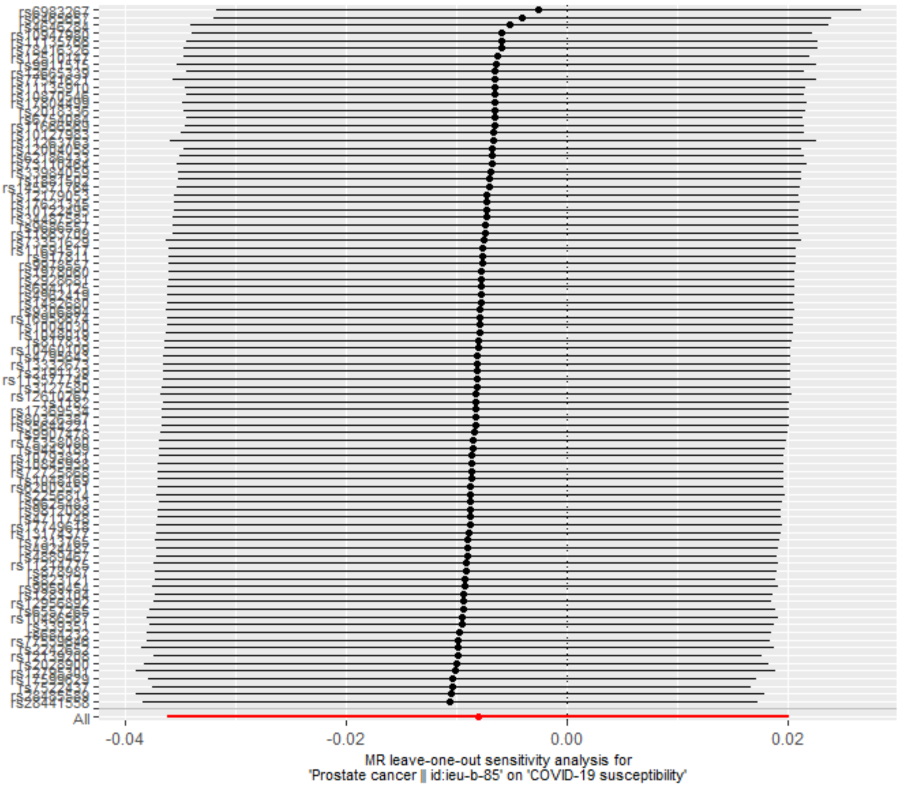

Figure S6H. The leave-one-out sensitivity analysis of the causal effect of prostate cancer on COVID-19 susceptibility.

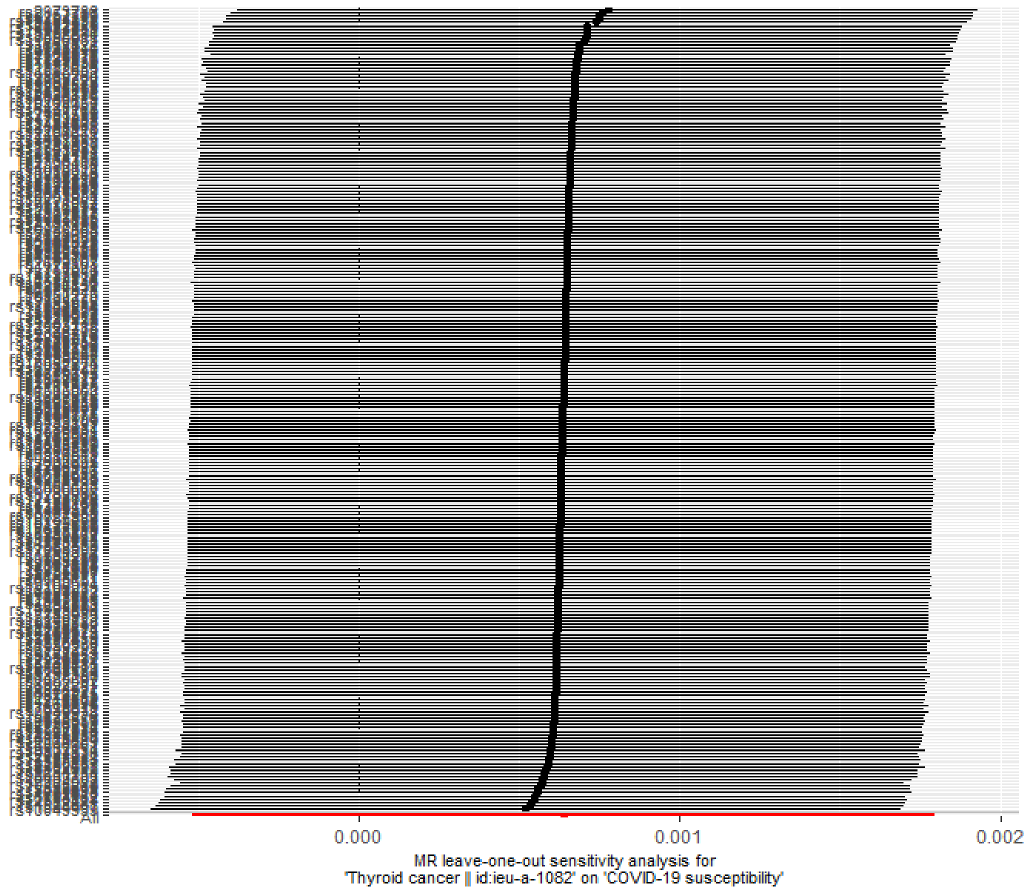

Figure S6I. The leave-one-out sensitivity analysis of the causal effect of thyroid cancer on COVID-19 susceptibility.

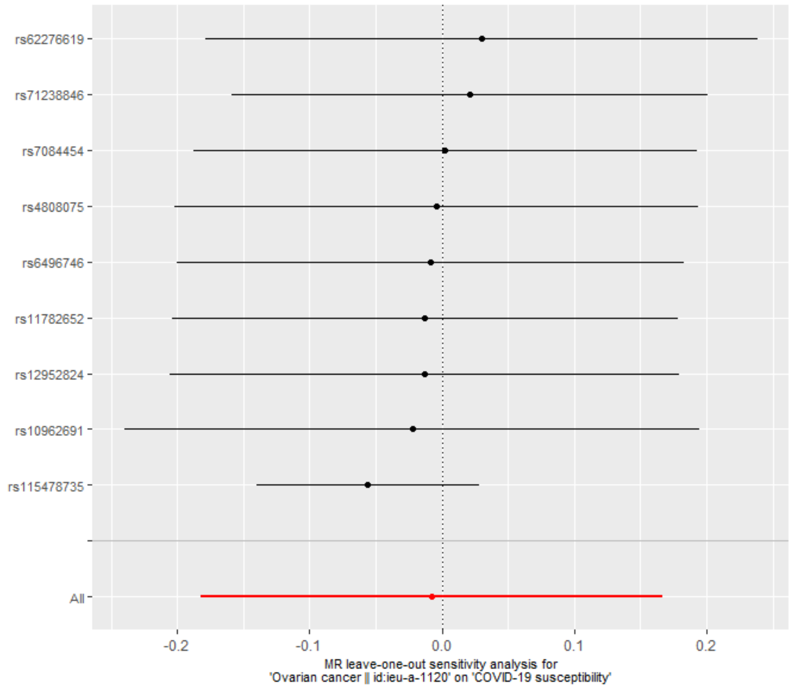

Figure S6J. The leave-one-out sensitivity analysis of the causal effect of ovarian cancer on COVID-19 susceptibility.

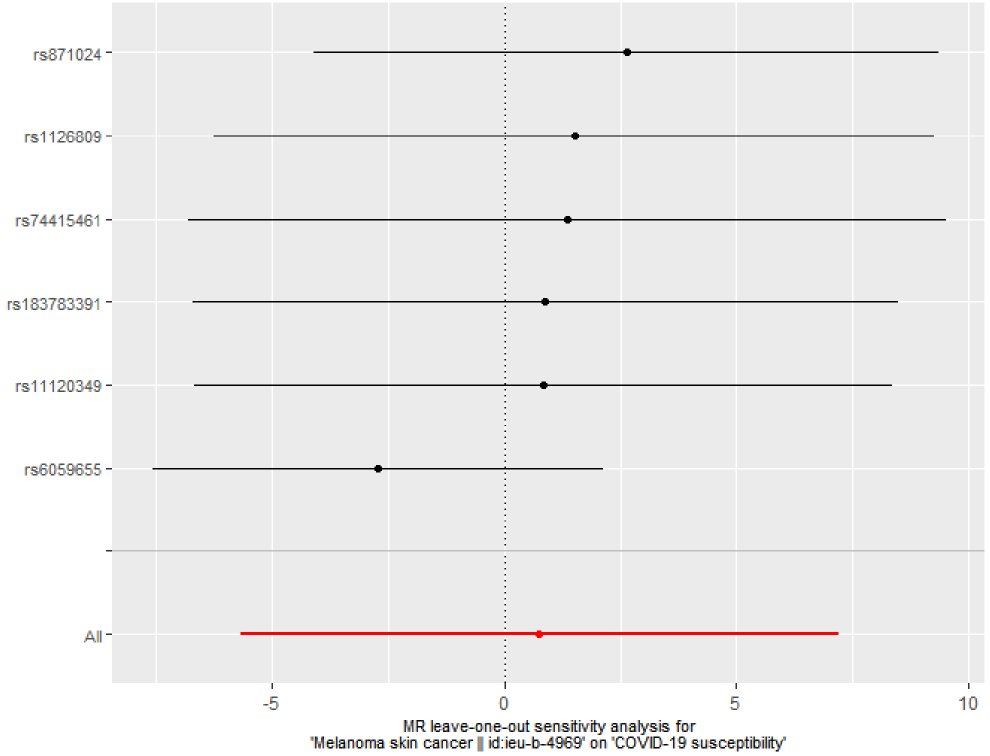

Figure S6K. The leave-one-out sensitivity analysis of the causal effect of melanoma on COVID-19 susceptibility.

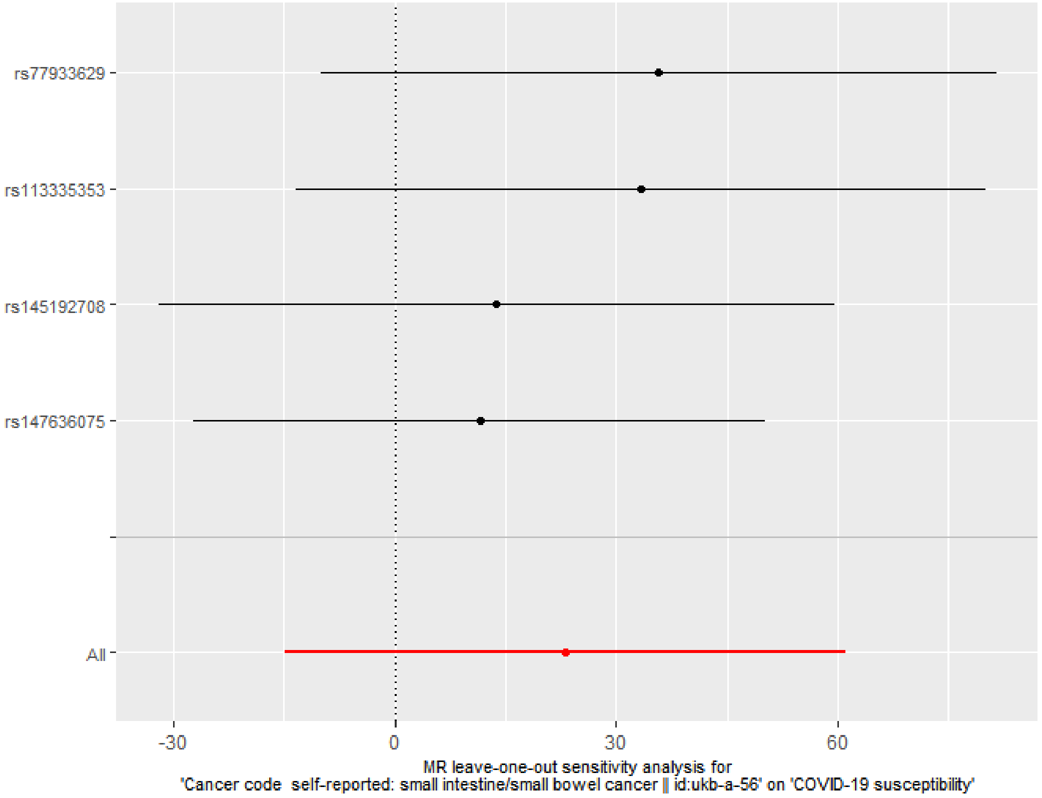

Figure S6L. The leave-one-out sensitivity analysis of the causal effect of small bowel cancer on COVID-19 susceptibility.

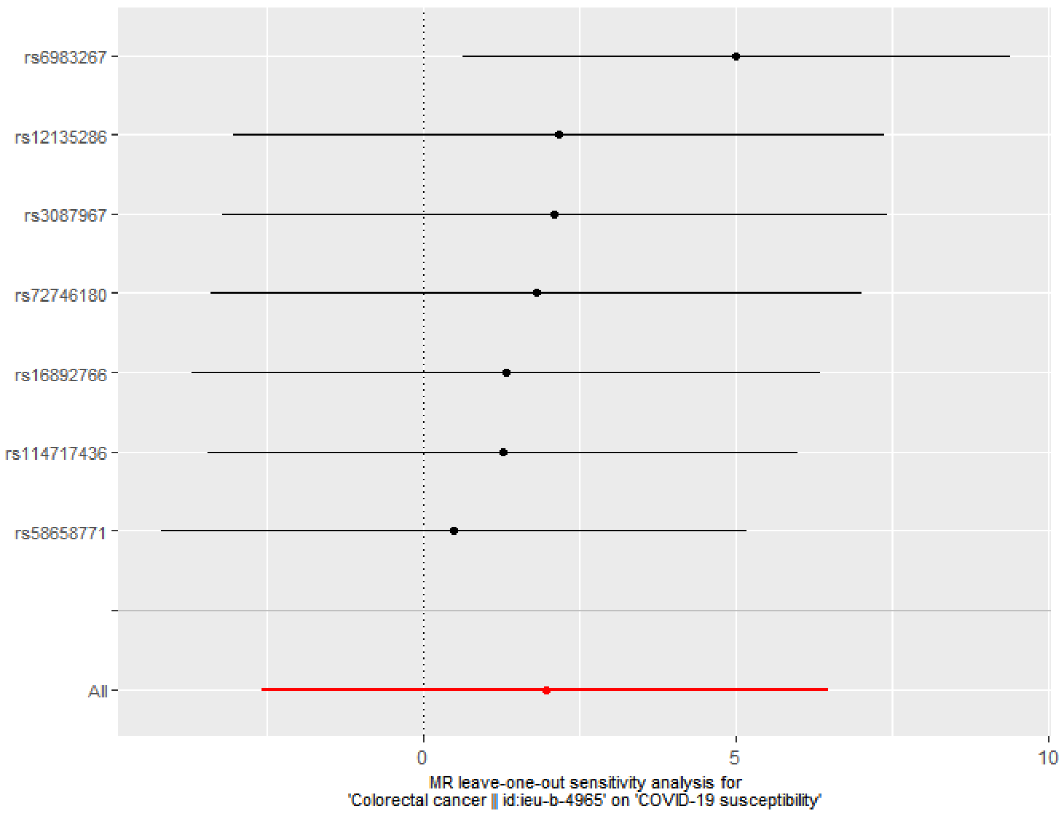

Figure S6M. The leave-one-out sensitivity analysis of the causal effect of colorectal cancer on COVID-19 susceptibility.

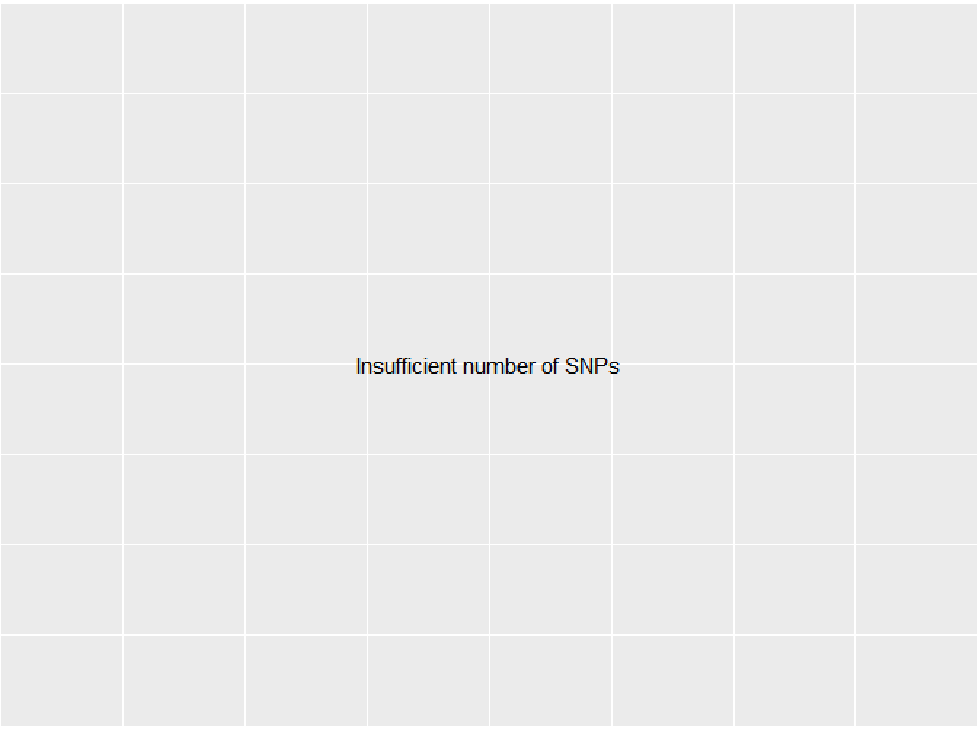

Figure S6N. The leave-one-out sensitivity analysis of the causal effect of oropharyngeal cancer on COVID-19 susceptibility.

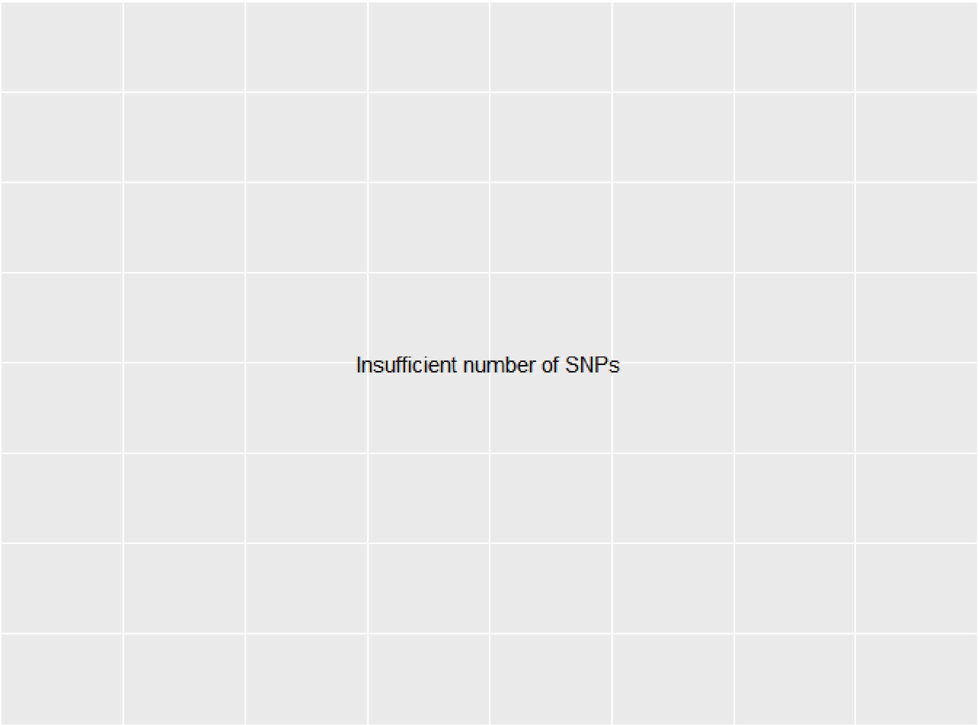

Figure S6O. The leave-one-out sensitivity analysis of the causal effect of lymphoma on COVID-19 susceptibility.

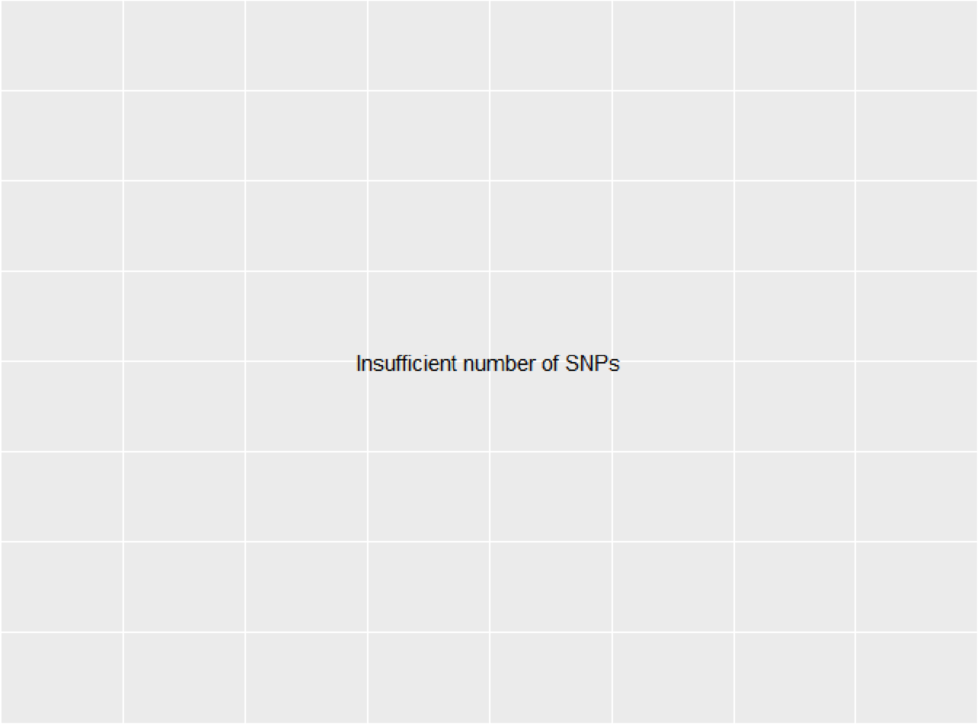

Figure S6P. The leave-one-out sensitivity analysis of the causal effect of cervix cancer on COVID-19 susceptibility.

Table S3. Causal effects of cancers on COVID-19 severity estimated by multivariable Mendelian randomization.

Variable adjustment	BMI				Education attainment				Intelligence				Income				Smoking				Alcohol consumption			
**Cancer**	**SNP**	**β**	**SE**	***p***	**SNP**	**β**	**SE**	***p***	**SNP**	**β**	**SE**	***p***	**SNP**	**β**	**SE**	***p***	**SNP**	**β**	**SE**	***p***	**SNP**	**β**	**SE**	***p***
**Overall cancer**	-	-	-	-	2	2.13	2.59	0.41	4	-2.91	3.03	0.34	4	0.71	3.30	0.83	4	-2.76	3.69	0.45	-	-	-	-
**Lung cancer**	2	0.03	0.07	0.70	4	0.08	0.06	0.18	5	0.03	0.07	0.61	5	0.04	0.06	0.51	5	-0.05	0.09	0.59	4	-0.03	0.11	0.77
**Squamous cell lung cancer**	1	0.01	0.06	0.80	1	0.01	0.07	0.87	1	0.03	0.10	0.75	1	0.06	0.12	0.62	3.0	-0.05	0.11	0.62	2.0	0.04	0.12	0.72
**Breast cancer**	37	0.11	0.07	0.12	73	0.02	0.06	0.67	87	0.05	0.05	0.38	100	0.04	0.05	0.43	97	0.05	0.05	0.38	96	0.05	0.05	0.34
**ER+ Breast cancer**	28	0.08	0.08	0.27	51	0.003	0.05	0.96	60	-0.01	0.05	0.79	70	-0.02	0.05	0.75	71	-0.01	0.05	0.83	69	-0.003	0.05	0.96
**ER- Breast cancer**	8	0.02	0.07	0.74	20	-0.03	0.07	0.69	23	0.02	0.07	0.82	24	0.01	0.06	0.85	24	0.03	0.06	0.67	25	0.05	0.07	0.41
**Endometrial cancer**	2	0.14	0.09	0.11	5	-0.06	0.10	0.54	9	0.11	0.10	0.26	10	0.06	0.08	0.46	10	0.01	0.10	0.88	9.0	0.10	0.10	0.35
**Prostate cancer**	30	0.03	0.06	0.58	64	0.001	0.04	0.98	70	0.001	0.05	0.98	82	-0.01	0.04	0.75	81	-0.01	0.05	0.82	82	-0.0001	0.05	1.00
**Thyroid cancer**	127	-0.001	0.002	0.55	196	-0.0008	0.002	0.69	221	-0.0004	0.002	0.82	239	-0.0008	0.002	0.66	237	-0.0005	0.002	0.76	234	-0.0008	0.002	0.67
**Ovarian cancer**	4	0.12	0.09	0.15	6	-0.12	0.10	0.21	7	-0.008	0.09	0.93	9	0.10	0.10	0.29	9	0.07	0.12	0.57	7.0	0.06	0.10	0.57
**Melanoma**	1	-1.45	6.61	0.83	5	-0.82	6.02	0.89	6	-0.34	7.46	0.96	6	1.41	8.29	0.87	6	-2.84	9.51	0.77	6	-3.14	9.16	0.73
**Small bowel cancer**	-	-	-	-	1	-7.99	39.57	0.84	4	66.15	45.54	0.15	4	35.28	49.53	0.48	5	86.50	45.10	0.06	4	82.11	53.35	0.12
**Colorectal cancer**	2	-2.47	5.08	0.63	6	-2.37	4.70	0.61	6	-5.71	5.51	0.30	7	-2.40	5.31	0.65	7	-0.25	4.91	0.96	6	-2.77	5.63	0.62
**Oropharyngeal cancer**	-	-	-	-	1	-20.85	22.75	0.36	2	-25.41	28.14	0.37	1	39.70	59.00	0.50	2	-43.71	44.89	0.33	2	-53.00	44.49	0.23
**Lymphoma**	-	-	-	-	-	-	-	-	1	4.78	16.51	0.77	1	18.00	20.95	0.39	1	-7.31	28.07	0.79	1	2.65	23.10	0.91
**Cervix cancer**	-	-	-	-	-	-	-	-	2	-20.89	12.39	0.09	2	-19.47	14.43	0.18	2	-17.55	15.79	0.27	2	-24.41	15.57	0.12

Table S4. Causal effects of cancers on COVID-19 hospitalization estimated by multivariable Mendelian randomization.

Variable adjustment	BMI				Education attainment				Intelligence				Income				Smoking				Alcohol consumption			
**Cancer**	**SNP**	**β**	**SE**	***p***	**SNP**	**β**	**SE**	***p***	**SNP**	**β**	**SE**	***p***	**SNP**	**β**	**SE**	***p***	**SNP**	**β**	**SE**	***p***	**SNP**	**β**	**SE**	***p***
**Overall cancer**	-	-	-	-	2	4.01	1.63	0.01[#]	4	-2.63	1.98	0.18	4	2.85	2.62	0.28	4	-1.93	2.32	0.41	4	1.19	2.12	0.58
**Lung cancer**	2	0.03	0.05	0.51	3	0.04	0.04	0.32	4	0.05	0.05	0.31	4	0.03	0.05	0.51	4	0.006	0.07	0.93	3	-0.0005	0.07	0.99
**Squamous cell lung cancer**	1	0.02	0.04	0.67	1	-0.05	0.05	0.29	1	0.03	0.07	0.67	1	-0.05	0.09	0.55	3	-0.01	0.07	0.87	2	0.04	0.07	0.52
**Breast cancer**	37	0.02	0.05	0.61	72	-0.0001	0.04	1.00	86	0.01	0.04	0.76	99	0.01	0.04	0.74	97	0.001	0.04	0.78	96	0.01	0.03	0.71
**ER+ Breast cancer**	28	0.02	0.05	0.66	51	0.007	0.03	0.85	59	-0.03	0.04	0.33	69	-0.02	0.04	0.63	70	-0.02	0.03	0.46	68	-0.02	0.03	0.50
**ER- Breast cancer**	8	0.02	0.05	0.70	19	-0.05	0.04	0.30	22	-0.01	0.05	0.75	23	0.001	0.05	0.98	24	-0.01	0.04	0.81	25	0.005	0.04	0.91
**Endometrial cancer**	2	0.02	0.06	0.77	5	-0.007	0.06	0.91	9	0.09	0.06	0.18	10	0.07	0.06	0.20	10	0.04	0.06	0.54	9	0.07	0.06	0.24
**Prostate cancer**	30	0.07	0.04	0.07	64	0.05	0.03	0.046[#]	70	0.05	0.03	0.10	82	0.05	0.03	0.06	81	0.04	0.03	0.15	82	0.05	0.03	0.11
**Thyroid cancer**	127	-0.0009	0.001	0.53	195	-0.0003	0.001	0.83	221	-0.0001	0.001	0.91	237	-0.0002	0.001	0.86	235	-0.0002	0.001	0.89	232	-0.0002	0.001	0.86
**Ovarian cancer**	4	0.07	0.06	0.24	6	-0.02	0.06	0.74	7	-0.06	0.06	0.34	9	0.03	0.07	0.69	9	0.01	0.09	0.90	7	-0.007	0.01	0.89
**Melanoma**	1	-0.22	4.55	0.96	5	-3.03	3.75	0.42	6	-3.01	4.52	0.51	6	-0.22	6.02	0.97	6	-2.77	5.26	0.60	6	-2.51	4.71	0.59
**Small bowel cancer**	-	-	-	-	-	-	-	-	1	45.31	37.55	0.23	2	56.63	52.13	0.28	2	89.14	44.82	0.047[#]	1	35.74	45.77	0.43
**Colorectal cancer**	2	-2.11	3.50	0.55	6	0.21	3.15	0.95	6	1.06	3.66	0.77	7	1.51	4.64	0.74	7	1.27	3.52	0.72	6	-0.18	3.45	0.96
**Oropharyngeal cancer**	-	-	-	-	-	-	-	-	-	-	-	-	-	-	-	-	-	-	-	-	-	-	-	-
**Lymphoma**	-	-	-	-	-	-	-	-	-	-	-	-	-	-	-	-	-	-	-	-	-	-	-	-
**Cervix cancer**	-	-	-	-	-	-	-	-	2	-12.42	8.17	0.13	2	-10.65	12.96	0.41	2	-16.78	11.00	0.13	2	-14.75	10.41	0.16

[#] Potential association ($p < 0.05$).

Table S5. Causal effects of cancers on COVID-19 susceptibility estimated by multivariable Mendelian randomization.

Variable adjustment	BMI				Education attainment				Intelligence				Income				Smoking				Alcohol consumption			
**Cancer**	**SNP**	**β**	**SE**	***p***	**SNP**	**β**	**SE**	***p***	**SNP**	**β**	**SE**	***p***	**SNP**	**β**	**SE**	***p***	**SNP**	**β**	**SE**	***p***	**SNP**	**β**	**SE**	***p***
**Overall cancer**	-	-	-	-	2	0.65	0.80	0.41	4	0.23	1.06	0.83	4	0.87	1.17	0.46	4	0.43	1.14	0.71	4	0.26	1.15	0.82
**Lung cancer**	2	-0.004	0.02	0.87	4	0.01	0.02	0.49	5	0.006	0.02	0.79	5	0.004	0.02	0.85	5	-0.003	0.03	0.92	4	-0.002	0.03	0.94
**Squamous cell lung cancer**	1	0.001	0.02	0.96	1	-0.007	0.02	0.75	1	0.03	0.10	0.75	1	-0.002	0.04	0.97	3	-0.03	0.04	0.41	2	-0.002	0.04	0.97
**Breast cancer**	37	0.001	0.02	0.96	73	-0.01	0.02	0.57	87	-0.01	0.02	0.51	100	-0.01	0.02	0.53	97	-0.01	0.02	0.48	96	-0.008	0.02	0.65
**ER+ Breast cancer**	28	-0.02	0.02	0.50	51	-0.02	0.02	0.25	60	-0.03	0.02	0.20	70	-0.02	0.02	0.23	71	-0.03	0.02	0.15	69	-0.02	0.02	0.29
**ER- Breast cancer**	8	0.003	0.02	0.91	20	-0.03	0.02	0.15	23	-0.04	0.03	0.15	24	-0.03	0.02	0.27	24	-0.02	0.02	0.31	25	-0.02	0.02	0.30
**Endometrial cancer**	2.0	0.008	0.03	0.78	5	-0.03	0.03	0.35	9.0	-0.01	0.03	0.76	10	0.01	0.03	0.61	10	0.001	0.02	0.97	9	-0.004	0.03	0.89
**Prostate cancer**	30	0.009	0.02	0.66	64	-0.008	0.01	0.56	70	-0.005	0.02	0.76	82	-0.004	0.01	0.76	81	-0.005	0.02	0.75	82	-0.002	0.02	0.88
**Thyroid cancer**	127	0.0002	0.0007	0.78	196	0.0008	0.0006	0.17	221	0.0007	0.0006	0.27	239	0.0007	0.0006	0.28	237	0.0006	0.0006	0.29	234	0.0006	0.0006	0.31
**Ovarian cancer**	4	0.03	0.03	0.31	6	-0.03	0.03	0.30	7	-0.06	0.03	0.07	9	0.002	0.05	0.97	9	-0.005	0.06	0.93	7	-0.03	0.03	0.32
**Melanoma**	1	-1.50	2.13	0.48	5	-0.60	1.83	0.74	6	-0.44	2.34	0.85	6	0.96	2.62	0.71	6	0.74	2.57	0.77	6	0.60	2.49	0.81
**Small bowel cancer**	-	-	-	-	-	-	-	-	3	28.72	17.06	0.09	3	10.12	20.33	0.62	4	19.39	17.35	0.26	3	19.35	18.90	0.31
**Colorectal cancer**	2	-1.89	1.65	0.25	6	0.48	1.62	0.77	6	-0.14	2.00	0.95	7	1.52	2.19	0.49	7	2.16	1.95	0.27	6	0.87	2.10	0.68
**Oropharyngeal cancer**	-	-	-	-	1	-5.96	7.23	0.41	2	2.19	9.46	0.82	1	-0.44	20.13	0.98	2	-16.91	11.45	0.14	2	-16.08	11.73	0.17
**Lymphoma**	-	-	-	-	-	-	-	-	1	-0.73	5.52	0.89	1	2.65	7.52	0.72	1	-1.74	7.66	0.82	1	-3.18	6.22	0.61
**Cervix cancer**	-	-	-	-	-	-	-	-	2	-7.01	4.47	0.12	2	-4.12	6.26	0.51	2	-5.18	6.35	0.41	2	-5.55	6.04	0.36

Table S6. Verification of the causal effects of cancers on COVID-19 outcomes.

Cancer	GWAS ID	Year	Cases	Controls	COVID-19 severity				COVID-19 hospitalization				COVID-19 susceptibility			
					**SNP**	**β**	**SE**	***p***	**SNP**	**β**	**SE**	***p***	**SNP**	**β**	**SE**	***p***
**Lung cancer**	ukb-a-54	2017	190	336,969	5	42.50	27.67	0.12	3	54.83	34.50	0.11	3	40.48	26.25	0.12
**Breast cancer**	ukb-b-16890	2018	10,303	452,630	15	1.09	3.64	0.76	15	0.21	1.72	0.90	15	-0.27	1.13	0.81
**Ovarian cancer**	ieu-b-4963	2021	1218	198,523	-	-	-	-	-	-	-	-	-	-	-	-
**Endometrial cancer**	ukb-b-13545	2018	1151	461,782	-	-	-	-	-			-	-	-	-	-
**Prostate cancer**	ieu-b-4809	2021	9132	173,493	27	-0.51	0.90	0.57	27	0.37	0.59	0.53	27	-0.20	0.31	0.52
**Thyroid malignant neoplasm**	finn-b-C3_THYROID_GLAND	2021	989	217,803	3	0.02	0.05	0.71	3	-0.03	0.03	0.34	3	0.01	0.02	0.45
**Situ melanoma**	finn-b-CD2_INSITU_MELANOMA	2021	393	218,399	-	-	-	-	-	-	-	-	-	-	-	-
**Colorectal cancer**	finn-b-C3_COLORECTAL	2021	3022	215,770	-	-	-	-	-			-	-	-	-	-
**Oropharyngeal cancer**	ieu-b-96	2016	1090	2928	-	-	-	-	-	-	-	-	-	-	-	-
**non-Hodgkin lymphoma**	finn-b-CD2_NONHODGKIN_NAS	2021	533	218,259	-	-	-	-	-	-	-	-	-	-	-	-
**Cervix situ carcinoma**	finn-b-CD2_INSITU_CERVIX_UTERI	2021	298	123,281	-	-	-	-	-			-	-	-	-	-
